# Supplementary material for: From Pioneer to Repressor: Bimodal foxd3 Activity Dynamically Remodels Neural Crest Regulatory Landscape In Vivo
Source: Dev Cell. 2018 Dec 3;47(5):608–628.e6. doi: 10.1016/j.devcel.2018.11.009 (PMC6286384; doi:10.1016/j.devcel.2018.11.009)
Supplement: Document S1. Figures S1–S6 [file mmc1.pdf]

Developmental Cell, Volume *47*

## Supplemental Information

# From Pioneer to Repressor: Bimodal foxd3 Activity Dynamically Remodels Neural Crest Regulatory Landscape *In Vivo*

**Martyna Lukoseviciute, Daria Gavriouchkina, Ruth M. Williams, Tatiana Hochgreb-Hagele, Upeka Senanayake, Vanessa Chong-Morrison, Supat Thongjuea, Emmanouela Repapi, Adam Mead, and Tatjana Sauka-Spengler**

*Figure S1*

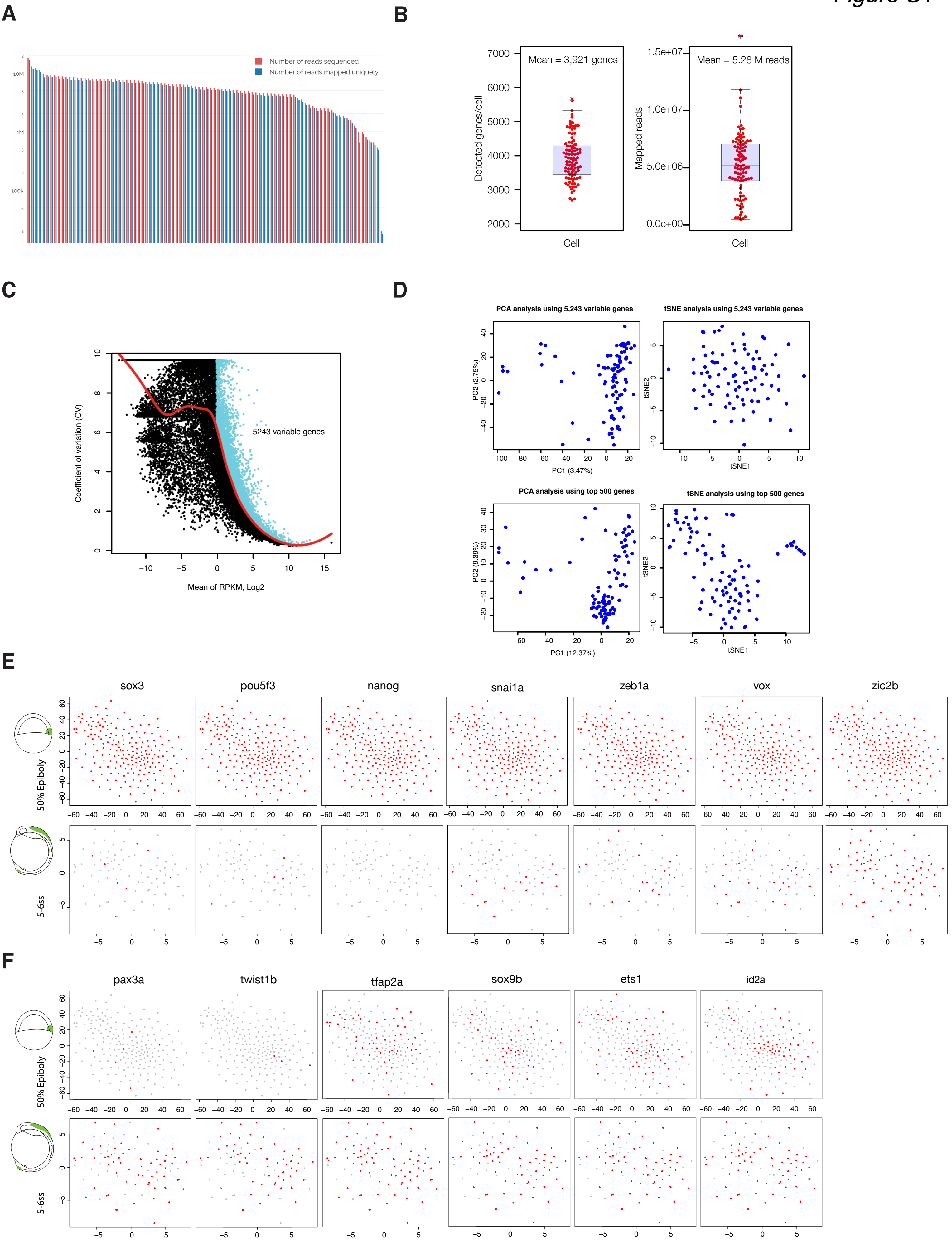

A
Number of reads sequenced
Number of reads mapped uniquely
10M
1M
100k
B
Mean = 3,921 genes
Detected genes/cell
Cell
Mean = 5.28 M reads
Mapped reads
Cell
C
Coefficient of variation (CV)
5243 variable genes
Mean of RPKM, Log2
D
PCA analysis using 5,243 variable genes
PC2 (2.75%)
PC1 (3.47%)
tSNE analysis using 5,243 variable genes
tSNE2
tSNE1
PCA analysis using top 500 genes
PC2 (9.39%)
PC1 (12.37%)
tSNE analysis using top 500 genes
tSNE2
tSNE1
E
sox3
pou5f3
nanog
snai1a
zeb1a
vox
zic2b
50% Epiboly
5-6ss
F
pax3a
twist1b
tfap2a
sox9b
ets1
id2a
50% Epiboly
5-6ss

**Figure S1. Single cell transcriptome and quality control (QC) analysis of foxd3-expressing cells from 5-6ss embryos. Related to Figure 1.**

**(A-B)** Grouped bar plot indicating number of sequenced and uniquely mapped reads shows 93/94 single cell transcriptomes are of excellent quality, with a mean of 5.24 M mapped reads and ~4,000 expressed genes mapped per cell.

**(C)** Identification of 5,243 genes with significantly highly variable expression across single premigratory NCs. Cyan dots indicate genes with a biological coefficient of variation (CV) of >0.50 at a 10% false discovery rate, classified as highly variable; black dots indicate all other genes; red line marks the CV threshold of 0.50 (i.e. the expected position of genes with 50% biological CV).

**(D)** Principal component analysis (PCA) and T-distributed stochastic neighbor embedding (tSNE) plots all 5,243 or top 500 most divergent genes identifies a small cluster of NC cells.

**(E)** tSNE plots for selected key pluripotency genes illustrate that while they are expressed at 50% *foxd3+* epiboly stage cells, they are not expressed or are expressed only in a few 5-6ss single *foxd3+* cells, except for *vox* and *zic2b* genes.

**(F)** tSNE plots for selected key NC specifier genes illustrate that while they are expressed in a majority of 5-6ss single *foxd3+* cells, they are absent or not abundant in 50% epiboly *foxd3+* cells.

*Figure S2*

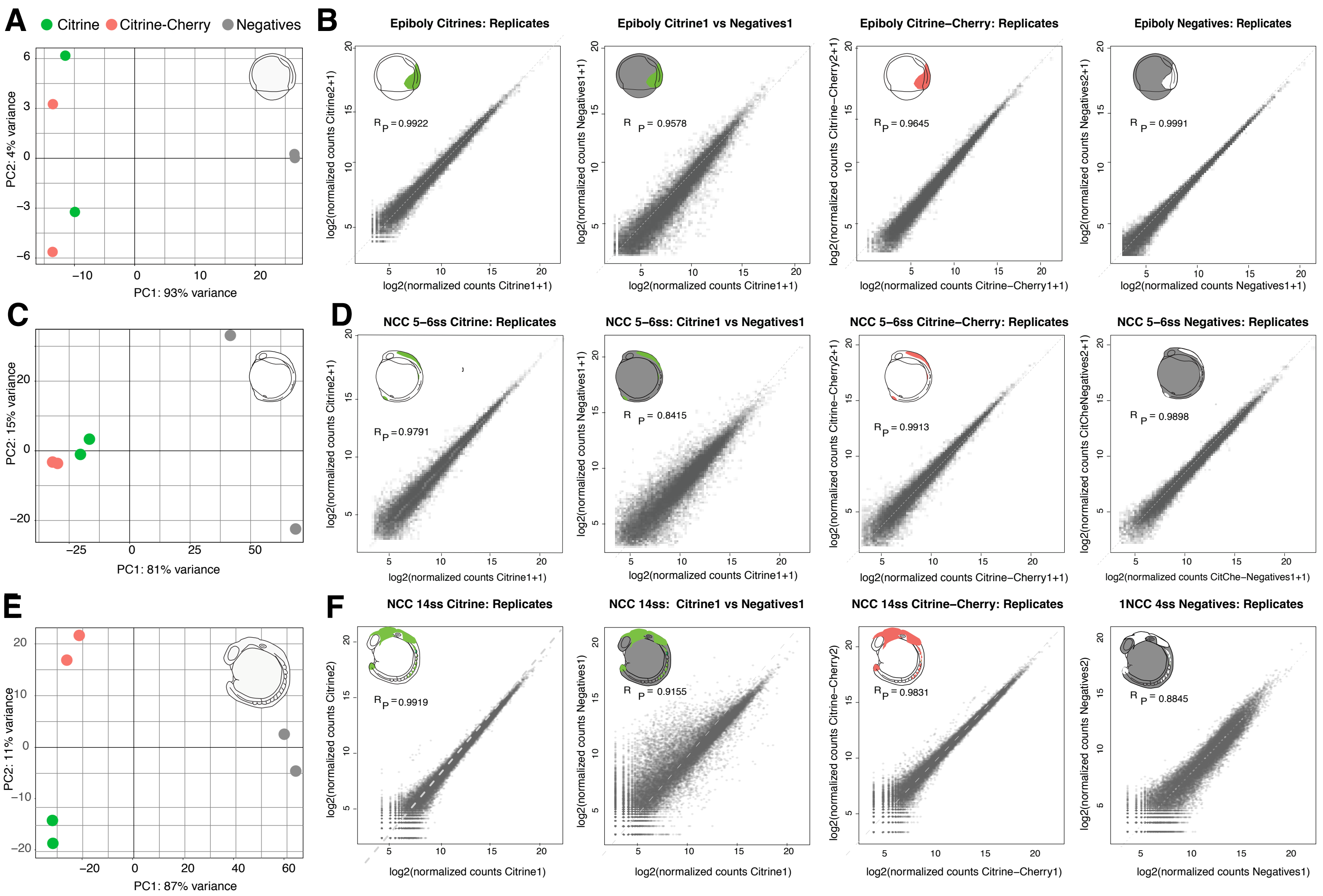


**G**

| | Epiboy Citrine1 | Epiboly Citrine2 | Epiboly Citrine-Cherry1 | Epiboly Citrine-Cherry2 | Epiboly Negatives1 | Epiboly Negatives2 | 5-6ss Citrine1 | 5-6ss Citrine2 | 5-6ss Citine-Cherry1 | 5-6ss Citine-Cherry2 | 5-6ss Negatives1 | 5-6ss Negatives2 | 14ss Citrine1 | 14ss Citrine2 | 14ss Citrine-Cherry1 | 14ss Citrine-Cherry2 | 14ss Negatives1 | 14ss Negatives2 |
|---|---|---|---|---|---|---|---|---|---|---|---|---|---|---|---|---|---|---|
| Epiboy Citrine1 | 1.00 | | | | | | | | | | | | | | | | | |
| Epiboly Citrine2 | 0.99 | 1.00 | | | | | | | | | | | | | | | | |
| Epiboly Citrine-Cherry1 | 0.99 | 0.99 | 1.00 | | | | | | | | | | | | | | | |
| Epiboly Citrine-Cherry2 | 0.98 | 0.99 | 0.96 | 1.00 | | | | | | | | | | | | | | |
| Epiboly Negatives1 | 0.96 | 0.97 | 0.94 | 0.97 | 1.00 | | | | | | | | | | | | | |
| Epiboly Negatives2 | 0.96 | 0.97 | 0.95 | 0.97 | 1.00 | 1.00 | | | | | | | | | | | | |
| 5-6ss Citrine1 | 0.84 | 0.81 | 0.85 | 0.78 | 0.79 | 0.80 | 1.00 | | | | | | | | | | | |
| 5-6ss Citrine2 | 0.82 | 0.81 | 0.82 | 0.79 | 0.80 | 0.80 | 0.98 | 1.00 | | | | | | | | | | |
| 5-6ss Citine-Cherry1 | 0.82 | 0.80 | 0.84 | 0.76 | 0.77 | 0.78 | 0.99 | 0.97 | 1.00 | | | | | | | | | |
| 5-6ss Citine-Cherry2 | 0.83 | 0.81 | 0.84 | 0.78 | 0.79 | 0.79 | 0.98 | 0.99 | 0.99 | 1.00 | | | | | | | | |
| 5-6ss Negatives1 | 0.89 | 0.89 | 0.87 | 0.87 | 0.93 | 0.93 | 0.84 | 0.86 | 0.83 | 0.85 | 1.00 | | | | | | | |
| 5-6ss Negatives2 | 0.89 | 0.89 | 0.88 | 0.87 | 0.92 | 0.92 | 0.85 | 0.87 | 0.85 | 0.86 | 0.99 | 1.00 | | | | | | |
| 14ss Citrine1 | 0.78 | 0.78 | 0.77 | 0.77 | 0.81 | 0.81 | 0.83 | 0.86 | 0.83 | 0.84 | 0.89 | 0.90 | 1.00 | | | | | |
| 14ss Citrine2 | 0.80 | 0.80 | 0.80 | 0.78 | 0.83 | 0.83 | 0.86 | 0.88 | 0.86 | 0.87 | 0.91 | 0.91 | 0.99 | 1.00 | | | | |
| 14ss Citrine-Cherry1 | 0.75 | 0.75 | 0.73 | 0.74 | 0.77 | 0.77 | 0.80 | 0.83 | 0.81 | 0.83 | 0.87 | 0.87 | 0.95 | 0.94 | 1.00 | | | |
| 14ss Citrine-Cherry2 | 0.78 | 0.77 | 0.77 | 0.75 | 0.79 | 0.79 | 0.85 | 0.85 | 0.85 | 0.86 | 0.88 | 0.89 | 0.97 | 0.96 | 0.98 | 1.00 | | |
| 14ss Negatives1 | 0.81 | 0.79 | 0.80 | 0.76 | 0.81 | 0.81 | 0.87 | 0.87 | 0.87 | 0.87 | 0.92 | 0.93 | 0.92 | 0.92 | 0.89 | 0.92 | 1.00 | |
| 14ss Negatives2 | 0.77 | 0.73 | 0.79 | 0.68 | 0.72 | 0.74 | 0.89 | 0.84 | 0.91 | 0.88 | 0.82 | 0.84 | 0.79 | 0.83 | 0.77 | 0.80 | 0.88 | 1.00 |

**Figure S2. Biological replicate transcriptome comparisons confirm experimental reproducibility. Related to Figures 1 and 2.**

**(A, C, E)** PCAs and Scatter plots **(B, D, F)** comparing *foxd3*-positive (Citrine), *foxd3*-negative and *foxd3*-mutant (Citrine_Cherry) RNA-seq samples at 3 stages of development (75% epiboly, 5-6ss, 14ss).

**(G)** Matrix presenting the correlation coefficients to all possible pairwise comparisons of replicates/samples.

*Figure S3*

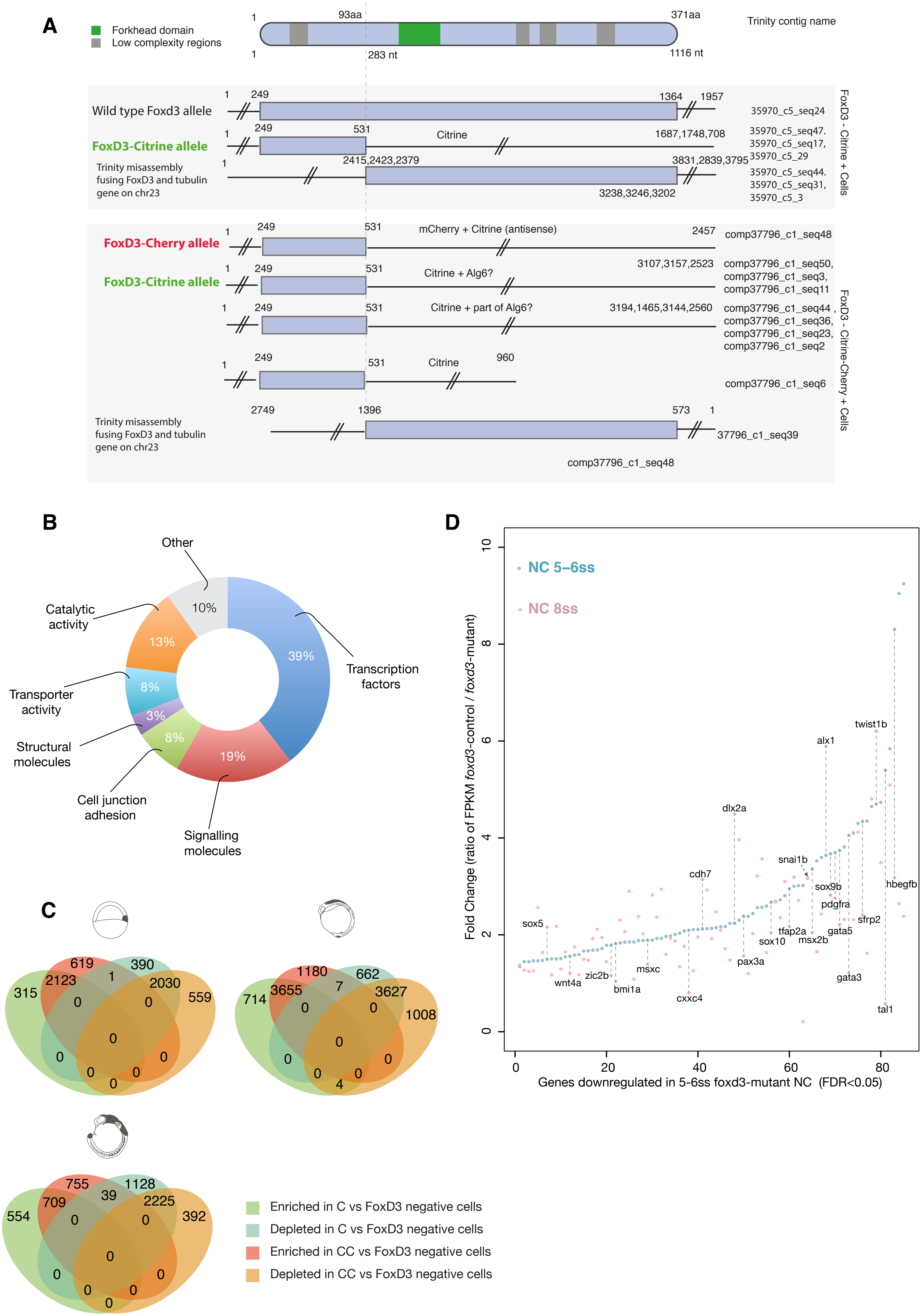
A
Forkhead domain
Low complexity regions
1
93aa
371aa
1
283 nt
1116 nt
Trinity contig name
Wild type Foxd3 allele
1
249
1364
1957
35970_c5_seq24
FoxD3-Citrine allele
1
249
531
Citrine
1687,1748,708
2415,2423,2379
35970_c5_seq47.
35970_c5_seq17,
35970_c5_29
Trinity misassembly
fusing FoxD3 and tubulin
gene on chr23
1
3831,2839,3795
3238,3246,3202
35970_c5_seq44.
35970_c5_seq31,
35970_c5_3
FoxD3 - Citrine + Cells
FoxD3-Cherry allele
1
249
531
mCherry + Citrine (antisense)
2457
comp37796_c1_seq48
FoxD3-Citrine allele
1
249
531
Citrine + Alg6?
3107,3157,2523
comp37796_c1_seq50,
comp37796_c1_seq3,
comp37796_c1_seq11
1
249
531
Citrine + part of Alg6?
3194,1465,3144,2560
comp37796_c1_seq44 ,
comp37796_c1_seq36,
comp37796_c1_seq23,
comp37796_c1_seq2
1
249
531
Citrine
960
comp37796_c1_seq6
Trinity misassembly
fusing FoxD3 and tubulin
gene on chr23
2749
1396
573
1
37796_c1_seq39
comp37796_c1_seq48
FoxD3 - Citrine-Cherry + Cells
B
Other
Catalytic activity
Transporter activity
Structural molecules
Cell junction adhesion
Signalling molecules
Transcription factors
10%
13%
8%
3%
8%
19%
39%
C
315
619
390
2123
1
2030
559
0
0
0
0
0
0
0
0
714
1180
662
3655
7
3627
1008
0
0
0
0
0
0
0
4
554
755
1128
709
39
2225
392
0
0
0
0
0
0
0
0
Enriched in C vs FoxD3 negative cells
Depleted in C vs FoxD3 negative cells
Enriched in CC vs FoxD3 negative cells
Depleted in CC vs FoxD3 negative cells
D
NC 5–6ss
NC 8ss
Fold Change (ratio of FPKM foxd3-control / foxd3-mutant)
Genes downregulated in 5-6ss foxd3-mutant NC (FDR<0.05)
0
2
4
6
8
10
0
20
40
60
80
twist1b
alx1
dlx2a
snai1b
cdh7
sox9b
hbegfb
pdgfra
sox5
sfrp2
tfap2a
gata5
sox10
msx2b
pax3a
msxc
zic2b
wnt4a
bmi1a
gata3
cxxc4
tal1

**Figure S3. Transcriptomic characterisation of foxd3-citrine and foxd3-cherry mutant cells. Related to Figures 1, 2 and 3.**

**(A)** An illustration of 5-6ss foxd3-mutant protein truncation derived from the *de novo* transcript Trinity assembly. This demonstrates that both foxd3-citrine and foxd3-cherry fusions generate a truncated foxd3 protein only coding for 93 out of 371 amino acids (truncated after 282 nucleotides of coding DNA sequence (CDS)) with no forkhead domain; and hence no protein dimerization or DNA binding is possible. All possible foxd3 transcript variations in the heterozygous foxd3-mutant fish carrying only a foxd3-citrine allele were: (1) foxd3 wt protein (2) truncated foxd3 after the 282nd nucleotide (3) misassembled trinity contigs that have incorrectly merged end of 93rd aa of foxd3 (chr6) with tubulin protein (chr23) due to the design of the FlipTrap cassette. All possible foxd3 transcript variations in the homozygous foxd3-mutant fish carrying foxd3-citrine and foxd3-cherry alleles were: (1) truncated foxd3 protein with Citrine & Alg6 (varying lengths) (2) truncated foxd3 with mCherry and Citrine (antisense) (3) the same misassembled trinity contigs as described before.

**(B)** The pie chart shows GO Molecular protein function distribution of the down-regulated genes in foxd3 mutants.

**(C)** Four-way Venn diagrams illustrating differentially expressed genes between both, genes enriched in foxd3-control (citrine - C) and homozygous foxd3-mutants (citrine, cherry - CC) over the negative cells, and depleted genes in foxd3-control (C) and homozygous foxd3-mutants (citrine, cherry - CC) over the negative cells at 75% epiboly, 5-6 somite stage (ss) and 14-16 ss embryos. Differential gene expression analysis was performed using DESeq2 v.1.18.1.

**(D)** Ranked scatter plot represents downregulated genes ranked from the lowest to the highest based on their differential expression fold change derived from the FPKM values in the 5-6ss foxd3-mutant vs control NC (labelled as blue dots). Pink dots represent the same set of genes (connected by dashed grey lines with blue dots) and their differential expression fold change in the 8ss foxd3-mutant vs control NC.
Since we found that the defect in expression of some, but not all, NC specification factors that were affected in pre-migratory foxd3-mutant NC is lessened, this suggests a possible partial compensation of the NC specification factors as the neural crest cells start delaminating at 8ss.

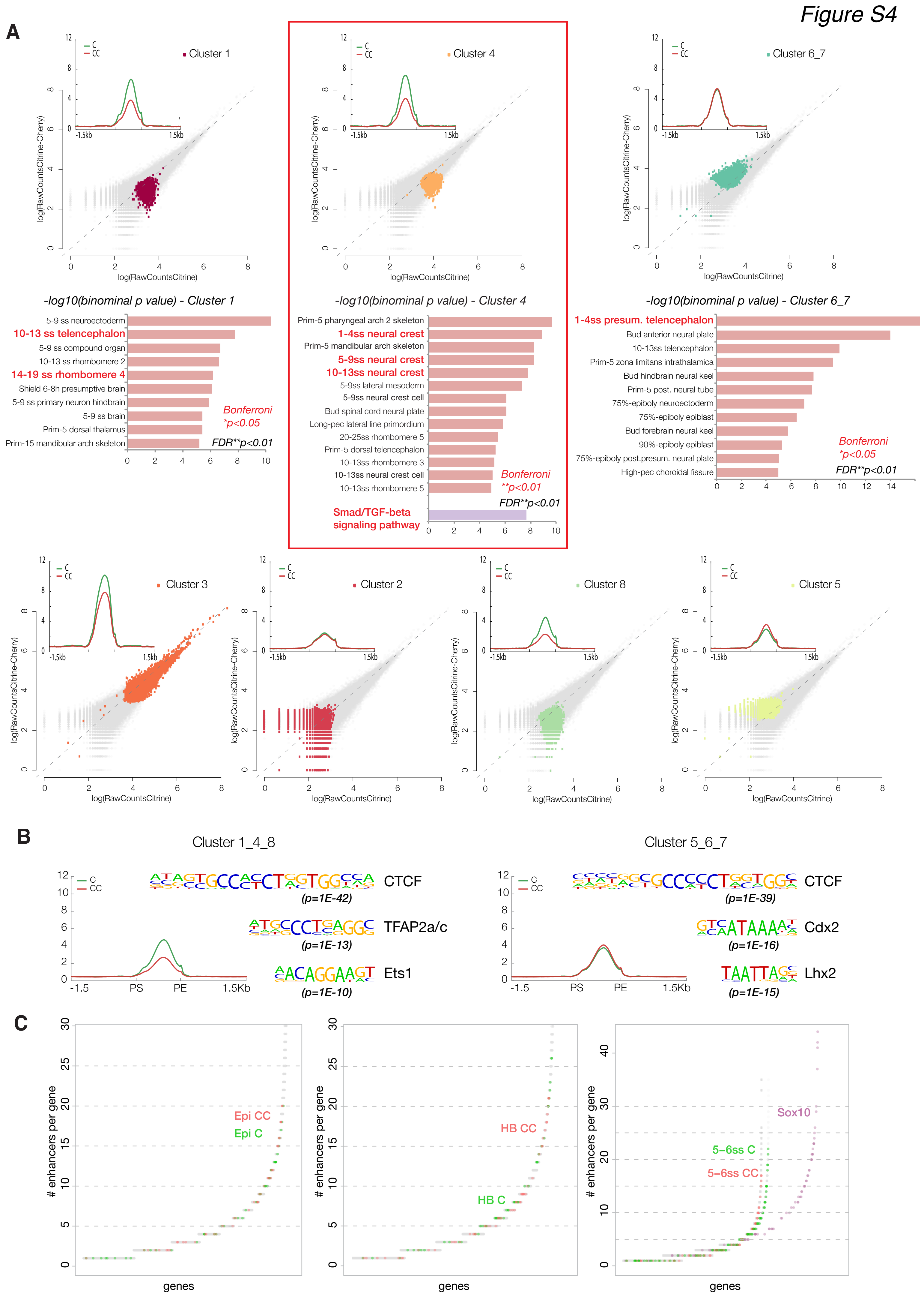
Figure S4
A
Cluster 1
Cluster 4
Cluster 6_7
Cluster 3
Cluster 2
Cluster 8
Cluster 5
C
CC
-1.5kb
1.5kb
log(RawCountsCitrine-Cherry)
log(RawCountsCitrine)
-log10(binominal p value) - Cluster 1
5-9 ss neuroectoderm
10-13 ss telencephalon
5-9 ss compound organ
10-13 ss rhombomere 2
14-19 ss rhombomere 4
Shield 6-8h presumptive brain
5-9 ss primary neuron hindbrain
5-9 ss brain
Prim-5 dorsal thalamus
Prim-15 mandibular arch skeleton
Bonferroni *p<0.05
FDR**p<0.01
-log10(binominal p value) - Cluster 4
Prim-5 pharyngeal arch 2 skeleton
1-4ss neural crest
Prim-5 mandibular arch skeleton
5-9ss neural crest
10-13ss neural crest
5-9ss lateral mesoderm
5-9ss neural crest cell
Bud spinal cord neural plate
Long-pec lateral line primordium
20-25ss rhombomere 5
Prim-5 dorsal telencephalon
10-13ss rhombomere 3
10-13ss neural crest cell
10-13ss rhombomere 5
Smad/TGF-beta signaling pathway
Bonferroni **p<0.01
FDR**p<0.01
-log10(binominal p value) - Cluster 6_7
1-4ss presum. telencephalon
Bud anterior neural plate
10-13ss telencephalon
Prim-5 zona limitans intrathalamica
Bud hindbrain neural keel
Prim-5 post. neural tube
75%-epiboly neuroectoderm
75%-epiboly epiblast
Bud forebrain neural keel
90%-epiboly epiblast
75%-epiboly post.presum. neural plate
High-pec choroidal fissure
Bonferroni *p<0.05
FDR**p<0.01
B
Cluster 1_4_8
Cluster 5_6_7
-1.5
PS
PE
1.5Kb
CTCF
(p=1E-42)
TFAP2a/c
(p=1E-13)
Ets1
(p=1E-10)
CTCF
(p=1E-39)
Cdx2
(p=1E-16)
Lhx2
(p=1E-15)
C
# enhancers per gene
genes
Epi CC
Epi C
HB CC
HB C
Sox10
5–6ss C
5–6ss CC

**Figure S4. Cluster analysis of differential chromatin accessibility from foxd3 mutant cells vs control. Related to Figure 4.**

**(A)** Mean density maps of merged profiles and corresponding scatterplots of raw counts for all *k*-means clusters featuring putative regulatory non-promoter elements with differential accessibility (ATAC-seq signal levels) in *foxd3*-mutant (CC, Citrine/Cherry) and controls (C, Citrine) at 5-6ss. The clusters were computed by applying *k*-means clustering algorithm from the seqMINER platform. Linear enrichment clustering of normalised ATAC-seq datasets from *foxd3*-mutant and *foxd3*-control NC cells was computed genome-wide over the region covering ± 1.5 kb from the centre of each ATAC-seq peak. Peak Calling the individual replicates and retaining only the peaks present in both replicates was used to generate sets of reference peaks. The datasets were normalised by pooling the replicates for each condition and down-sampling the files to the same number of mapped reads. Clusters 1-4-8 (6,777-4,493-8,245 el.) contain lower signal element with prominent differences between mutant and controls (C>>CC), Pearson corr. $R_{Cl1}$=0.11; $R_{Cl4}$=0.16; $R_{Cl8}$=0.17, clusters 5-67 (8,182-3,905-6,070 el.) comprise elements of equally low signal, but with comparable accessibility (C≈CC), $R_{Cl5}$=0.17; $R_{Cl67}$=0.5 and Cluster 3 (17,390 el.) features highly accessible regions with broad ATAC-seq peak distribution that showed intermediate signal decrease in mutant (C>CC), $R_{Cl3}$=0.96.

Bar plots depict functional annotation of *k*-clusters 1, 4 and 67 showing specific enrichment of zebrafish gene expression ontology terms linked to brain (Clusters 1 and 67) and NC development (Cluster 4). Clusters 2, 5 and 8 did not yield any significant terms. Statistical significance calculated by both binominal and hypergeometric tests (Bonferroni $**p < 0.01$). In particular, elements from *k*-cluster 4 elements that feature moderate accessibility and clear defect in the accessibility in the *foxd3*-mutants, are specifically enriched in the zebrafish gene expression ontology terms linked to all stages of NC development under study here (1-4ss, 5-9ss and 10-13ss), as well as to the brain development. Interestingly, cluster 4 elements were also highly significantly associated to the Smad/TGFbeta signalling pathway (FDR<0.01) and assigned to genes that are normally downregulated in migrating NC and de-repressed in *foxd3*-mutant (*acvr2aa, bmp2b, bmp4, bmpr1a/b, bmpr2b, dand5, foxh1, smad2, smad3a/3b, smad6b, smurf1, tgfb3*; Bonferroni $**p<0.01$). Finally, association to molecules involved in negative regulation of neurogenesis, that were also de-repressed in *foxd3*-mutants by 14ss (*epha4a*, *musk*, *robo2*, *slit3*, *her3*, *her9*, *neurog1*, *notch3*; FDR $**p<0.01$), suggests that Cluster 4 elements could be acting in repression.

**(B)** Top Transcription Factor Binding Site (TFBS) motifs enriched in other *k*-means clusters of lower accessibility (Clusters 1-4-8 and 5-67). CTCF was the top enriched binding motif (p=1E-39) in. Elements from clusters 1-4-8 that display defective chromatin accessibility in *foxd3*-mutant cells were also enriched in NC motifs, in particular TFAP2a and Ets1, but not Sox10, whereas cluster 5-67 elements (no differential accessibility in foxd3 mutants) harboured a non-crest neural regulatory code (Cdx, Lhx, Hox). Thus the enhancer elements whose accessibility is dependent on foxd3 appear to display unifying features of a NC enhancer.

**(C)** Plots representing genes assigned to all putative regulatory elements, identified in *foxd3*-mutant (CC) and control (C) cells at 4 stages of development (Epi, 75% epiboly; HB, head reagion of the bud stage embryo 1-2ss; 5-6ss NC and sox10-expressing cells at 16ss) ranked by the number of associated elements.

Putative regulatory elements (all identified non-promoter ATAC-seq elements) identified in *foxd3*-mutant (CC) and control (C) NC were associated to the genes expressed at each corresponding stage and the assigned genes were ranked by the number of elements associated with them. Key developmental factors are most often regulated by multiple *cis*-regulatory elements, acting in concert to yield strong gene expression and defined tissue-specific patterns. Ranking plots show that a large proportion of expressed genes, considered as highly regulated loci, laid beyond the inflexion point on the ranking plot and was assigned a minimum of 3 regulatory elements. Interestingly, while the number of genes and the number of elements assigned per gene at epiboly stage did not differ, by 5-6ss we detected clear increase in expressed genes, elements and number of elements assigned per gene in control NC cells, suggesting that the defect of accessibility at distal cis-regulatory elements was clearly linked to the gene expression differences in *foxd3*-mutant (CC) NC cells. Finally, by late NC differentiation stage, the number of regulated genes and associated elements increases overall.

# Figure S5

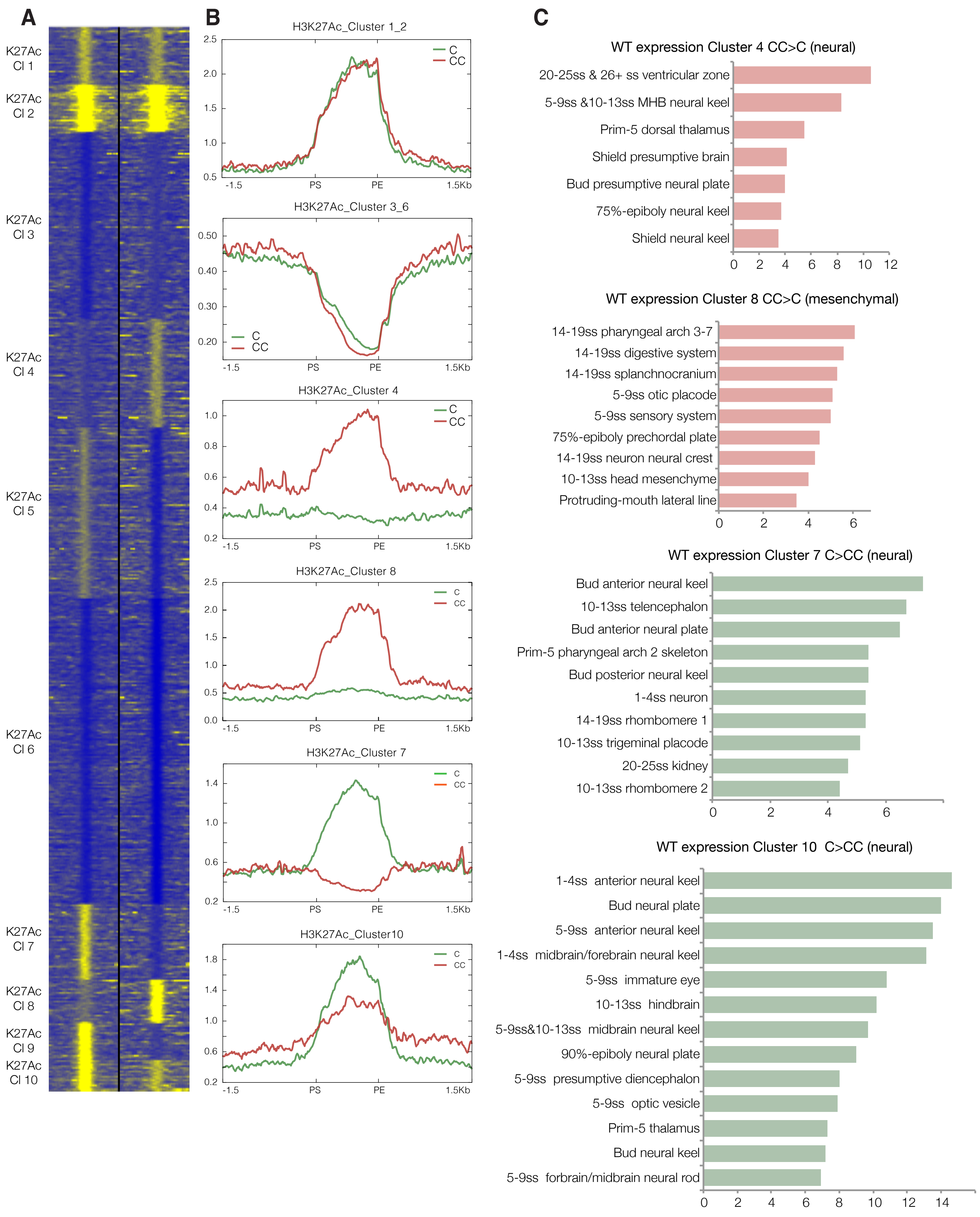

A
K27Ac Cl 1
K27Ac Cl 2
K27Ac Cl 3
K27Ac Cl 4
K27Ac Cl 5
K27Ac Cl 6
K27Ac Cl 7
K27Ac Cl 8
K27Ac Cl 9
K27Ac Cl 10
B
H3K27Ac_Cluster 1_2
H3K27Ac_Cluster 3_6
H3K27Ac_Cluster 4
H3K27Ac_Cluster 8
H3K27Ac_Cluster 7
H3K27Ac_Cluster10
C
CC
-1.5
PS
PE
1.5Kb
C
WT expression Cluster 4 CC>C (neural)
20-25ss & 26+ ss ventricular zone
5-9ss &10-13ss MHB neural keel
Prim-5 dorsal thalamus
Shield presumptive brain
Bud presumptive neural plate
75%-epiboly neural keel
Shield neural keel
WT expression Cluster 8 CC>C (mesenchymal)
14-19ss pharyngeal arch 3-7
14-19ss digestive system
14-19ss splanchnocranium
5-9ss otic placode
5-9ss sensory system
75%-epiboly prechordal plate
14-19ss neuron neural crest
10-13ss head mesenchyme
Protruding-mouth lateral line
WT expression Cluster 7 C>CC (neural)
Bud anterior neural keel
10-13ss telencephalon
Bud anterior neural plate
Prim-5 pharyngeal arch 2 skeleton
Bud posterior neural keel
1-4ss neuron
14-19ss rhombomere 1
10-13ss trigeminal placode
20-25ss kidney
10-13ss rhombomere 2
WT expression Cluster 10 C>CC (neural)
1-4ss anterior neural keel
Bud neural plate
5-9ss anterior neural keel
1-4ss midbrain/forebrain neural keel
5-9ss immature eye
10-13ss hindbrain
5-9ss&10-13ss midbrain neural keel
90%-epiboly neural plate
5-9ss presumptive diencephalon
5-9ss optic vesicle
Prim-5 thalamus
Bud neural keel
5-9ss forbrain/midbrain neural rod

**Figure S5. Cluster analysis of differential non-promoter H3K27 acetylation from foxd3 mutant cells vs control. Related to Figure 6.**

**(A,B)** Complete heatmap **(A)** depicting *k*-means linear enrichment clustering of H3K27Ac signal across non-promoter ATAC-seq peaks in foxd3-mutant and control NC at 5-6ss (*k*=10) and **(B)** associated mean density maps of merged profiles of all clusters are shown. Enrichment was computed genome-wide over the region covering ± 1.5 kb from the centre of each ATAC-seq peak, ATAC-seq reference set as described in Figure S4 was used. We identified clusters with increased H3K27Acetylation in the foxd3 mutant (C<CC, K27Ac_Clusters 4 and 8) and clusters with decreased K3K27Ac signal in foxd3 mutants (C>CC, K27AC-Clusters 5, 7, 9 and 10). **(C)** Bar plots depicting functional annotation of K27Ac_Clusters 4, 8, 7 and 10 depicting specific enrichment of zebrafish gene expression ontology terms obtained by GREAT tool are shown (Bonferroni **$p < 0.01$).

# Figure S6

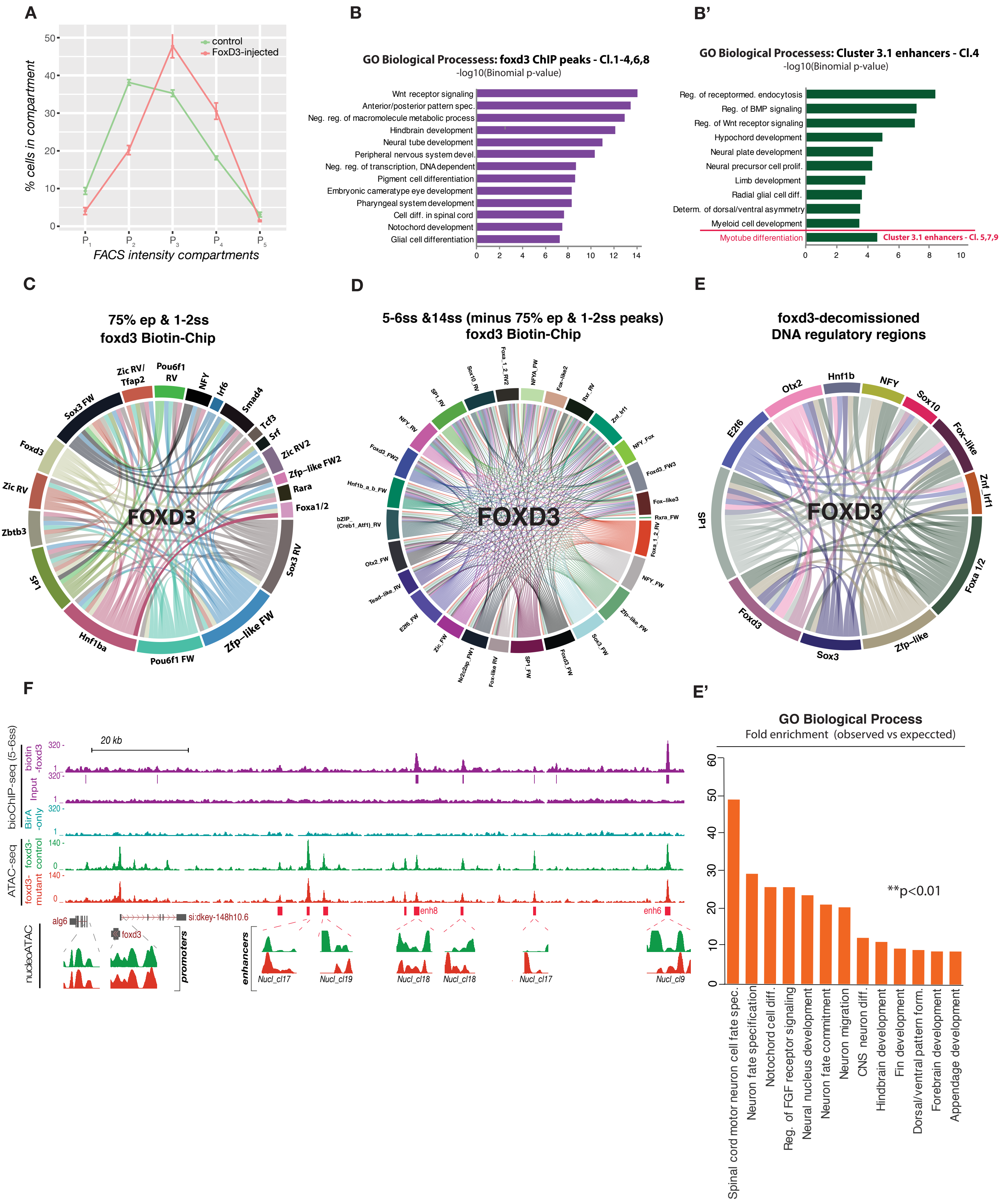
A
% cells in compartment
control
FoxD3-injected
FACS intensity compartments
B
GO Biological Processess: foxd3 ChIP peaks - Cl.1-4,6,8
-log10(Binomial p-value)
Wnt receptor signaling
Anterior/posterior pattern spec.
Neg. reg. of macromolecule metabolic process
Hindbrain development
Neural tube development
Peripheral nervous system devel.
Neg. reg. of transcription, DNA dependent
Pigment cell differentiation
Embryonic cameratype eye development
Pharyngeal system development
Cell diff. in spinal cord
Notochord development
Glial cell differentiation
B'
GO Biological Processess: Cluster 3.1 enhancers - Cl.4
-log10(Binomial p-value)
Reg. of receptormed. endocytosis
Reg. of BMP signaling
Reg. of Wnt receptor signaling
Hypochord development
Neural plate development
Neural precursor cell prolif.
Limb development
Radial glial cell diff.
Determ. of dorsal/ventral asymmetry
Myeloid cell development
Myotube differentiation
Cluster 3.1 enhancers - Cl. 5,7,9
C
75% ep & 1-2ss
foxd3 Biotin-Chip
FOXD3
D
5-6ss &14ss (minus 75% ep & 1-2ss peaks)
foxd3 Biotin-Chip
FOXD3
E
foxd3-decomissioned
DNA regulatory regions
FOXD3
F
20 kb
bioChIP-seq (5-6ss)
biotin-foxd3
Input
BirA-only
ATAC-seq
foxd3-control
foxd3-mutant
alg6
foxd3
si:dkey-148h10.6
enh8
enh6
nucleoATAC
promoters
enhancers
Nucl_cl17
Nucl_cl19
Nucl_cl18
Nucl_cl18
Nucl_cl17
Nucl_cl9
E'
GO Biological Process
Fold enrichment (observed vs expeccted)
**p<0.01
Spinal cord motor neuron cell fate spec.
Neuron fate specification
Notochord cell diff.
Reg. of FGF receptor signaling
Neural nucleus development
Neuron fate commitment
Neuron migration
CNS neuron diff.
Hindbrain development
Fin development
Dorsal/ventral pattern form.
Forebrain development
Appendage development

**Figure S6. Putative mechanisms of the foxd3 bimodality and potential co-interactors for NC gene regulation. Related to Figure 7.**

**(A)** FACS graph portraying a number of foxd3-mCherry expressing cells and underlying fluorescence intensities of control (green) and foxd3 mRNA injected (pink) embryos. P1-P5 – compartments of different fluorescence levels from the lowest to the highest. The graph illustrates an overall cellular fluorescence increase upon foxd3 overexpression in zebrafish embryos.

**(B,B′)** Bar plots showing gene ontology (GO) biological processes derived from GREAT analysis that are associated with the regulation of DNA-elements showing increased accessibility upon foxd3 overexpression. **(B)** These regulatory sequences were shown to be directly bound by foxd3 by our biotin ChIP-seq analysis (merged 1-4,6,8 k-mean subclusters (out of eight subclusters) showing similar enhanced accessibility signature that were clustered using all foxd3 possible foxd3 ChIP-seq peaks from our data sets). **(B′)** These regulatory sequences belong to the core NC enhancer cluster 3.1 (subcluster 3.1_4 elements and merged comparable signature subclusters 3.1_5, 7, 9 (single GO term under the red line) showing enhanced accessibility; they were obtained by k-mean analysis (out of ten 3.1 subclusters)).

**(C,D)** Circle plots showing different significant non-combined transcription factor (TF) motif enrichment underlying the **(C)** 'early NC' foxd3 biotin ChIP-seq peaks and statistically significant 2-way combinations between these factors that are hypothesised to act together with foxd3 during its activator stage and **(D)** 'late NC' foxd3 biotin ChIP-seq peaks and statistically significant 2-way combinations between these factors that are hypothesised to act together or in competition with foxd3 during its repressive stage.

**(E)** Circle plot showing significant 2-way TF co-interactions on the regulatory elements that get directly decommissioned by foxd3. **(E′)** Bar plot showing gene ontology (GO) biological terms significantly enriched (**$p<0.01$) to the nearest expressed genes of the same DNA regulatory elements that get directly compacted upon foxd3 binding by the 14ss.

**(F)** Nucleosomal occupancy tracks (NucleoATAC) showing nucleosome positions within the regulatory elements (promoters and enhancers) in foxd3 mutant (red) and control (green) cells.
