# Supplementary material for: From Pioneer to Repressor: Bimodal foxd3 Activity Dynamically Remodels Neural Crest Regulatory Landscape In Vivo
Source: Dev Cell. 2018 Dec 3;47(5):608–628.e6. doi: 10.1016/j.devcel.2018.11.009 (PMC6286384; doi:10.1016/j.devcel.2018.11.009)
Supplement: Document S2. Article plus Supplemental Information [file mmc2.pdf]

Developmental Cell

Article

From Pioneer to Repressor: Bimodal foxd3 Activity Dynamically Remodels Neural Crest Regulatory Landscape *In Vivo*

Graphical Abstract

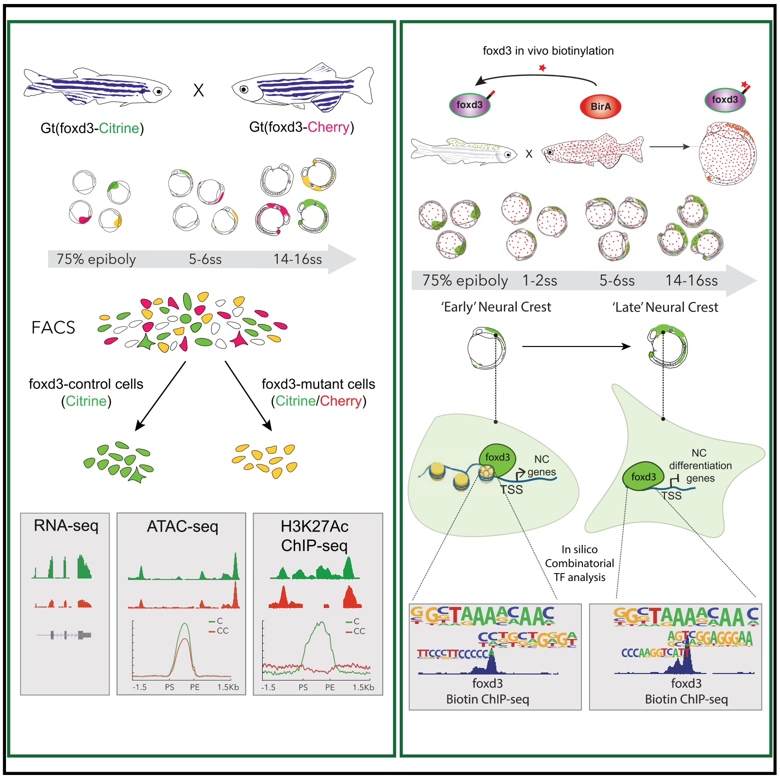

Highlights

- FoxD3 primes neural crest specification by modulating distal enhancers
- FoxD3 represses a number of neural crest migration and differentiation genes
- In neural crest, FoxD3 acts to switch chromatin from "permissive" to "repressive"
- Distinctive gene regulatory mechanisms underlie the bimodal action of FoxD3

Authors

Martyna Lukoseviciute,
Daria Gavriouchkina,
Ruth M. Williams, ...,
Emmanouela Repapi, Adam Mead,
Tatjana Sauka-Spengler

Correspondence

tatjana.sauka-spengler@imm.ox.ac.uk

In Brief

Through transcriptional and epigenomic profiling of foxd3-mutant zebrafish neural crest cells (NCCs) and whole-genome mapping of FoxD3 binding, Lukoseviciute et al. uncover bimodal FoxD3 action across NCC development. FoxD3 acts as a pioneer factor to prime genes for NCC specification before switching to being a repressor to control migration and differentiation.

Lukoseviciute et al., 2018, Developmental Cell *47*, 608–628
December 3, 2018 © 2018 The Authors. Published by Elsevier Inc.
https://doi.org/10.1016/j.devcel.2018.11.009

CellPress

Developmental Cell
Article

From Pioneer to Repressor: Bimodal foxd3 Activity Dynamically Remodels Neural Crest Regulatory Landscape *In Vivo*

Martyna Lukoseviciute,[1,4] Daria Gavriouchkina,[1,4,5] Ruth M. Williams,[1,4] Tatiana Hochgreb-Hagele,[1] Upeka Senanayake,[1] Vanessa Chong-Morrison,[1] Supat Thongjuea,[2] Emmanouela Repapi,[3] Adam Mead,[2] and Tatjana Sauka-Spengler[1,6,*]
[1]Radcliffe Department of Medicine, MRC Weatherall Institute of Molecular Medicine, University of Oxford, Oxford OX3 9DS, UK
[2]Molecular Haematology Unit, MRC Weatherall Institute of Molecular Medicine, University of Oxford, Oxford OX3 9DS, UK
[3]MRC WIMM Centre for Computational Biology Research Group, MRC Weatherall Institute of Molecular Medicine, University of Oxford, Oxford OX3 9DS, UK
[4]These authors contributed equally
[5]Present address: Okinawa Institute of Science and Technology, Molecular Genetics Unit, Onna 904-0495, Japan
[6]Lead Contact
*Correspondence: tatjana.sauka-spengler@imm.ox.ac.uk
https://doi.org/10.1016/j.devcel.2018.11.009

SUMMARY

The neural crest (NC) is a transient embryonic stem cell-like population characterized by its multipotency and broad developmental potential. Here, we perform NC-specific transcriptional and epigenomic profiling of *foxd3*-mutant cells *in vivo* to define the gene regulatory circuits controlling NC specification. Together with global binding analysis obtained by foxd3 biotin-ChIP and single cell profiles of *foxd3*-expressing premigratory NC, our analysis shows that, during early steps of NC formation, foxd3 acts globally as a pioneer factor to prime the onset of genes regulating NC specification and migration by re-arranging the chromatin landscape, opening *cis*-regulatory elements and reshuffling nucleosomes. Strikingly, foxd3 then gradually switches from an activator to its well-described role as a transcriptional repressor and potentially uses differential partners for each role. Taken together, these results demonstrate that foxd3 acts bimodally in the neural crest as a switch from "permissive" to "repressive" nucleosome and chromatin organization to maintain multipotency and define cell fates.

INTRODUCTION

The winged-helix, forkhead transcription factor (TF) FoxD3 is an important stem cell factor that functions reiteratively during development. At early stages of development, it is thought to maintain pluripotency of epiblast cells. In embryonic stem (ES) cells, its loss leads to premature differentiation into mesendodermal lineages while ectodermal lineage markers are reduced (Liu and Labosky, 2008). Later, FoxD3 plays a critical role in the specification and subsequent differentiation of the neural crest (NC), a remarkable transitory and multipotent embryonic cell population. NC cells are specified at the border of the forming central nervous system (neural plate border, NPB), but then undergo an epithelial to mesenchymal transition (EMT) to delaminate from the neural tube, migrating into the periphery where they give rise to diverse derivatives such as peripheral ganglia, craniofacial skeleton, and pigmentation of the skin (Sauka-Spengler and Bronner-Fraser, 2008). Although individual neural crest cells are multipotent (Baggiolini et al., 2015; Bronner-Fraser and Fraser, 1988), it has been a matter of debate whether the NC population as a whole is homogeneous or a heterogeneous mixture of cells specified toward a particular fate (Harris and Erickson, 2007; Krispin et al., 2010; Nitzan et al., 2013).

The molecular mechanisms by which FoxD3 influences ES cell development *in vitro* have been extensively studied. During the transition from naive to primed pluripotency cells, FoxD3 represses enhancers by recruiting H3K4 demethylase, Lsd1, resulting in a decrease of active enhancer marks and an increase in inactive enhancer marks (Respuela et al., 2016). During the subsequent pluripotent to epiblast cell transition, FoxD3 primes enhancers by co-recruiting nucleosome remodelling and deacetylase complex members Brg1 and histone deacetylases 1/2 (HDAC1/2). As a result, different subsets of enhancers get fully activated or are kept repressed during differentiation, depending on the effects mediated by HDAC1/2 removal or retention (Krishnakumar et al., 2016). These studies led to the realization that FoxD3-mediated gene regulation in ES cells may function via modulation of associated enhancers.

In contrast to ES cells, the molecular mechanisms through which neural crest cells transition from pluripotent cells to fate restricted cells in the embryo and the role of FoxD3 therein remain poorly understood. A neural crest gene regulatory network (GRN) that describes the genes expressed during NC ontogeny and their epistatic relationships has been proposed (Sauka-Spengler and Bronner-Fraser, 2008). Within this framework, FoxD3 is known to act downstream of NPB genes and upstream of factors mediating EMT (Betancur et al., 2010; Simões-Costa and Bronner, 2015). In the zebrafish embryo, *foxd3* is one of the earliest zygotically expressed genes (Lee et al., 2013), first detected during epiboly in the dorsal mesendoderm and ectoderm (Wang et al., 2011) and later in the NPB, tailbud mesoderm, and floor plate (Odenthal and Nüsslein-Volhard, 1998). A second phase

This is an open access article under the CC BY license (http://creativecommons.org/licenses/by/4.0/).

CellPress

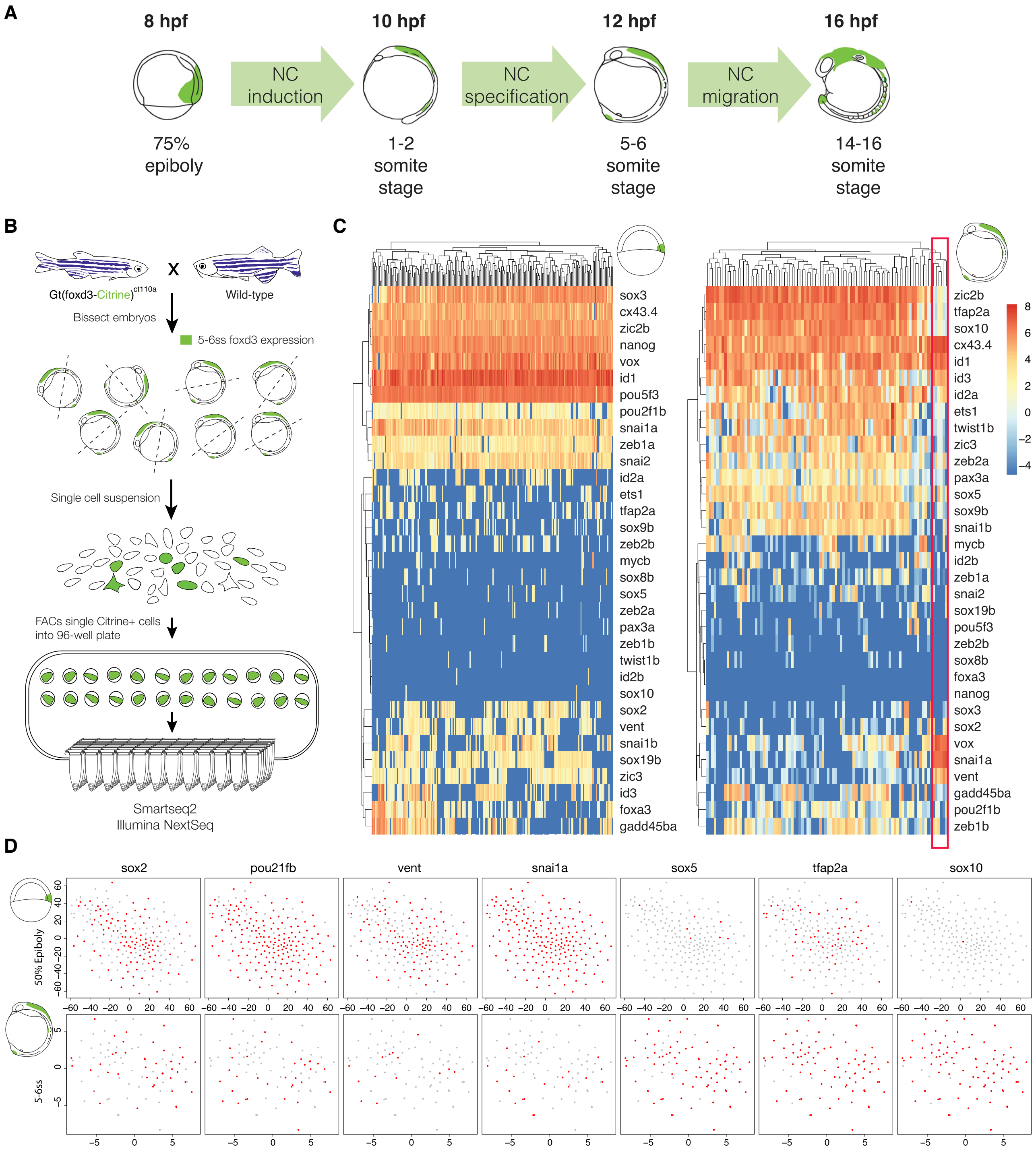

Figure 1. Transcriptome Characterization of *foxd3*-Expressing NC

(A) Zebrafish embryo stages examined in this study (hpf – hours post fertilization). 75% epiboly—a gastrulation stage during which embryonic shield and hypoblast are formed. 1–2 and 5–6 somite stages—induced and specified premigratory neural crest (NC), respectively. 14–16ss – migratory NC. Foxd3 expression is labeled in green.

(B) Experimental pipeline for obtaining *foxd3*-expressing cells and performing single-cell RNA-seq (scRNA-seq). The genetrap zebrafish line, *Gt(foxd3-citrine)^{ct110}*, expressing foxd3-Citrine fusion is outcrossed to wild-type resulting in fluorescent signal in endogenous *foxd3*+ cells, enabling their isolation by FACS. 5–6ss Citrine-positive NC cells are collected into individual wells of the 96-well plate and processed for smartseq2-based scRNA-seq. Total of 94 cells was sorted with two empty, External RNA Controls Consortium (ERCC)-only wells left as controls.

(C) Heatmaps illustrating the hierarchical clustering of *foxd3*+ single cells at 75% epiboly (200 cells) and 5–6ss (93 cells) and showing transcriptional levels (depicted in Log2 RPKM) of selected NC and stem cell genes. NC cells that express negligent levels of *bona fide* NC specifiers (*zic2b*, *tfap2a*, *sox10*, *twist1b*, *ets1*,

(legend continued on next page)

of *foxd3* expression occurs in premigratory neural crest cells within the neural folds at all axial levels. Even later, *foxd3* becomes restricted to a subset of migrating cranial neural crest cells and is downregulated in the trunk crest, reappearing in neural crest-derived peripheral glia and other tissues such as the somites (Gilmour et al., 2002; Kelsh et al., 2000).

Here, we tackle the molecular mechanisms by which *foxd3* influences neural crest development by taking advantage of wild-type and mutant zebrafish *foxd3* lines to characterize the transcriptional and epigenetic landscape of *foxd3*-expressing cells *in vivo*. First, using single-cell RNA sequencing, we demonstrate that *foxd3*-expressing cells display a distinct and homogeneous molecular signature in a stage-specific manner. Intriguingly, we observed a decoupling of the different strategies employed by foxd3 to regulate gene expression over the course of neural crest ontogeny. Contrasting with its previously defined role as a transcriptional repressor, early knockout *foxd3*, in the premigratory crest, resulted in global downregulation of neural crest genes, favoring the idea that foxd3 acts as a pioneer factor during early stages of neural crest development. This was shown by comprehensively analyzing the effects of foxd3 depletion on chromatin accessibility, histone modifications, and nucleosome positioning, as well as by generating in-depth stage-specific foxd3 binding maps using our newly developed biotin chromatin immunoprecipitation sequencing (ChIP-seq) method. At later stages, foxd3 assumes its known role as a transcriptional repressor. Biotin ChIP-seq confirms the direct association of foxd3 with a number of genes, both downregulated and upregulated in the foxd3 mutant, exemplifying its bimodal function in NC gene regulation. By exploring the underlying foxd3 DNA binding codes across different stages of NC development (early-activating and late-repressing stages), we show that these two contrasting foxd3 activities are likely to be achieved by engaging differential co-partners. This in turn possibly leads to the recruitment of different chromatin remodeling complexes, such as Brg1 or PRC1 members, that mediate chromatin priming and compaction, respectively. In summary, we demonstrate that foxd3 drives several independent chromatin-organizing mechanisms, switching from activator to repressor roles to orchestrate multiple regulatory programs during NC formation, starting with priming early NC specification to regulating essential signaling pathways, maintaining multipotency by controlling stem cell programs, and preventing premature migration and differentiation into neuronal NC derivatives.

RESULTS

Single-Cell RNA-Seq Identifies Distinctive Transcriptional Signatures between foxd3+ Stem Cells and foxd3+ NC Cells

In this study, we examined foxd3 roles throughout premigratory and migratory NC ontology in zebrafish embryos (Figure 1A). We first looked at 75% epiboly stage embryos during which gastrulation takes place, forming the embryonic shield and hypoblast. We then looked at premigratory NC stages, which occur during the zebrafish segmentation period and when NC gets induced and later specified at the NPB 1–2 and 5–6 somite stages (ss), respectively. Finally, we examined a migratory NC stage (14–16ss).

As a first step in characterizing the global developmental functions of foxd3, we examined *foxd3*-positive stem and NC cells at a single-cell level, to ascertain whether these seemingly different cell populations were non-heterogeneous. There have been debates in the literature regarding whether the premigratory neural crest is a homogeneous or heterogeneous cell population (Harris and Erickson, 2007; Krispin et al., 2010; Nitzan et al., 2013). We used a gene trap line, *Gt(foxd3-citrine)*^{ct110} (Hochgreb-Hägele and Bronner, 2013), which drives the expression of foxd3-Citrine fusion, yielding a fluorescent signal in endogenous *foxd3+* cells. This line enabled us to carry out RNA sequencing (RNA-seq) on single *foxd3*-expressing NC cells (single-cell RNA-seq [scRNA-seq]) isolated from the developing zebrafish embryos by fluorescence-activated cell sorting (FACS) (Figure 1B). Metrics show that our libraries are of excellent quality (high complexity, a high number of uniquely mapped sequencing reads, and up to ~5,500 transcripts detected per cell; Figures S1A and S1B). We performed t-distributed stochastic neighbor embedding (tSNE) and principal-component analyses (PCAs) of single-cell transcriptomes at 5–6ss, based on either all 5,243 or the top 500 most divergent genes (Figures S1C and S1D). Surprisingly, we failed to identify multiple NC-specific subpopulations but instead singled out a small population of NC cells which expressed extremely low levels of *bona fide* NC specifiers (*zic2b*, *tfap2a*, *sox10*, *twist1b*, *ets1*, or *pax3a*) and lower levels of *foxd3* itself. However, these cells expressed high levels of *lratb*, *cxcr4b*, and *ved*, as well as other markers of multipotent progenitors (*snai1a*, *vent*, *vox*, and *cx43.4*; Figure 1C, outlined in red), suggesting that they may represent pluripotent non-specified NC progenitors maintained in premigratory NC.

In addition, to identify potential differences between the *foxd3*-positive stem and NC cells, we compared the transcriptional *foxd3+* single-cell signatures at 50% epiboly (5.3 hours post fertilization [hpf]) (Satija et al., 2015) and 5–6ss (this study) (Figures 1C, 1D, S1E, and S1F). tSNE plots comparing expression of core NC and stem cell genes in single *foxd3+* cells show that, at both stages, nearly all *foxd3+* cells expressed the pluripotency factor *cx43.4* and NPB specifiers *id1* and *zic2b* at high levels, while more than 50% of cells expressed *pou2f1b*, *zic3*, and id3 (Figures 1C and 1D). Interestingly, however, the expression of core pluripotency factors was different at the two stages examined. The majority of *foxd3+* single cells at 50% epiboly expressed *Oct4* orthologs (*pou5f3*, *pou2f1b*), *SoxB* ortholog (*sox3*), *nanog*, and *vox* (reminiscent of *Xenopus XOct*, *Xsox2*, and *XVent*) (Buitrago-Delgado et al., 2015). In contrast, 5–6ss single *foxd3+* cells did not express *nanog*, and only a few cells expressed *sox3* or *pou5f3* at low levels (Figures 1C and S1E), while the greater portion of cells expressed paralogous factors *sox2*, *pou2f1b*, *vent*, and *vox* (Figures 1C, 1D, and S1E). Furthermore, *foxd3+*

or *pax3a*) but high levels of *lratb*, *cxcr4b*, and *ved*, as well as other markers of stemness (*snai1a*, *vent*, *vox*, and *cx43.4*), possibly representing pluripotent non-specified NC progenitors maintained in premigratory NC (boxed and labeled in red).

(D) tSNE plots for selected stem cell (*sox2*, *pou2f1*, *vent*) and NC genes (*snai1a*, *sox5*, *tfap2a*, *sox10*) indicate individual epiblast, and NC cells do not reveal cell subpopulations. Clustering of 5–6ss NC cells identifies a small group of cells that appear to be pluripotent non-specified NC progenitors. See Figure S1 for scRNA-seq quality control (QC) and more details.

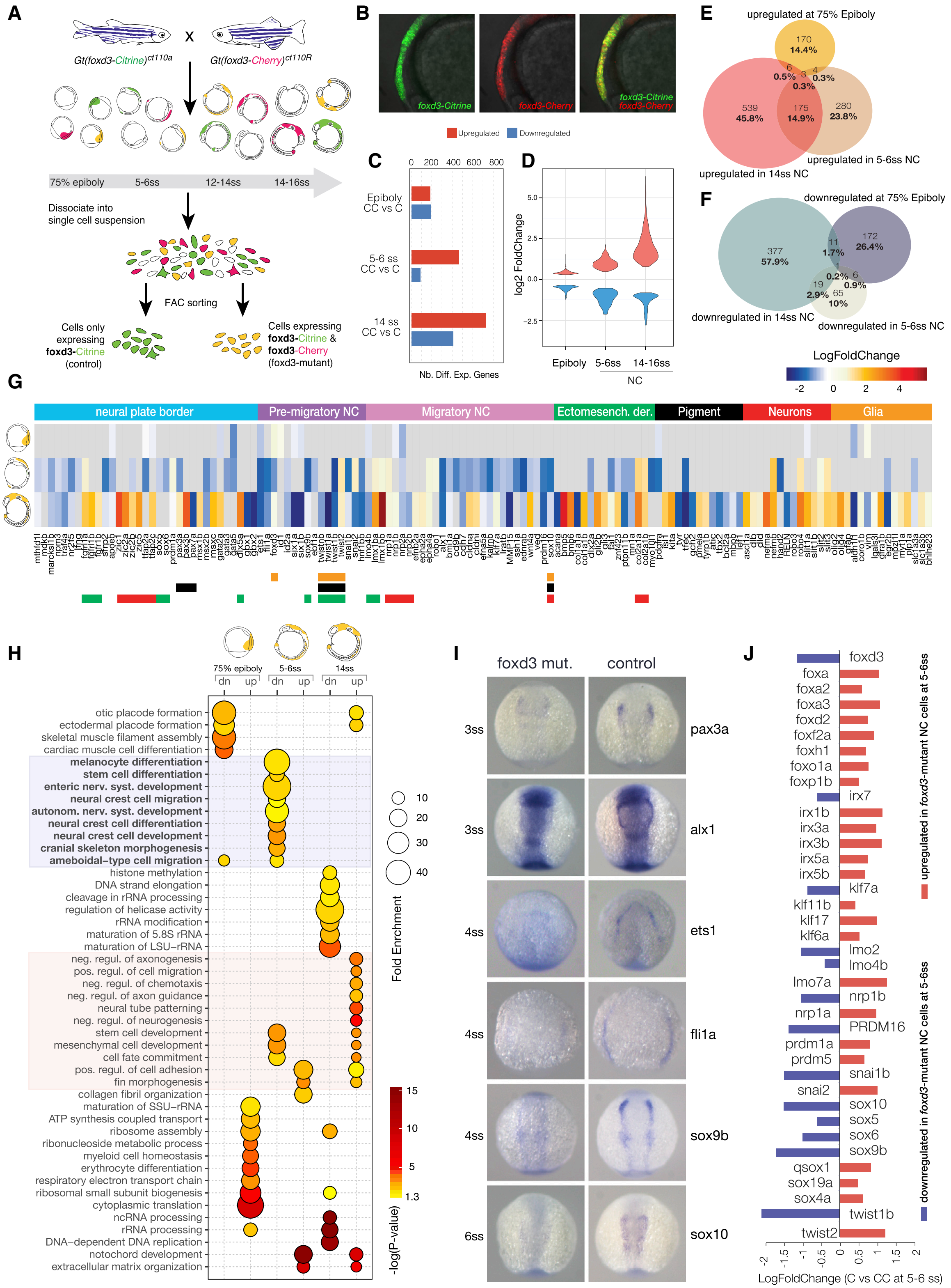

Figure 2. Transcriptional Profiling of *foxd3* Mutant NC

(A) Experimental strategy for obtaining *foxd3*-mutant (yellow) and *foxd3*-control (green) NC cells. Mutant (Citrine/Cherry; CC) and control (Citrine only; C) NC cells were isolated by FACS from crosses of heterozygote fluorescent *foxd3* transgenic fish, *foxd3-mCherry and foxd3-Citrine* at three stages—75% epiboly, 5–6ss, and 14ss.

(legend continued on next page)

gastrula progenitors expressed a different complement of orthologs of EMT factors compared to premigratory NC, with *zeb1a*, *snai1a*, and *snai2* present at 50% epiboly, but poorly expressed in most 5–6ss *foxd3*+ NC cells, which favored *zeb1b/2a* and *snai1b* (Figures 1C, 1D, and S1E). NC specifiers (*sox5*, *sox10*, *twist1b*, *pax3a*) were expressed at high levels in almost all 5–6ss *foxd3*+ NC cells but were absent from the majority of 50% epiboly *foxd3*+ cells, where early NC specifiers (*snai1b*, *sox9b*, *tfap2a*, *ets1*, *id2a*) were expressed more pervasively (Figures 1C, 1D, and S1F). In *Xenopus*, it has been suggested that neural crest cells may retain blastula-stage competence (Buitrago-Delgado et al., 2015). We found that orthologs of *Xenopus* genes were indeed expressed in the 50% epiboly *foxd3*+ cells in zebrafish (Figure 1C). However, as described above, our data revealed that 5–6ss *foxd3*+ cells do not express the same but rather paralogous pluripotency factors to those found in the epiblast. This suggests a possible redeployment of a paralogous GRN rather than maintenance of the epiblast GRN in the newly specified neural crest, in agreement with the recent single-cell-based analysis performed in both frog and fish (Briggs et al., 2018), and thus challenging the proposition that NC cells are residual blastula cells (Buitrago-Delgado et al., 2015).

Taken together, the results show that both *foxd3*+ epiblast and *foxd3*+ premigratory NC cell populations are non-heterogeneous, as well as distinctive from one another.

Knockout of foxd3 Leads to Downregulation of NC Specifier Genes at Premigratory NC Stages and Upregulation of NC Differentiation Genes at Migratory NC Stages

We next inquired how foxd3 depletion affects NC progenitor cells on a transcriptional level using two zebrafish transgenic lines *Gt(foxd3-mCherry)*^{ct110R} and *Gt(foxd3-Citrine)*^{ct110} (Figures 2A and 2B) in which the fluorescent reporter proteins interrupt the DNA binding domain, creating mutant fluorescent *foxd3* alleles (Hochgreb-Hägele and Bronner, 2013). These lines were crossed, and dissociated embryonic cells obtained from corresponding clutches were fluorescence activated cell (FAC)-sorted to isolate Citrine only expressing *foxd3*+ cells (C) as a control and *foxd3*-mutant cells expressing both fluorophores (Citrine and Cherry; CC) (Figures 2A and 2B). PCA and scatterplots of normalized read counts comparing RNA-seq biological replicates show a high level of reproducibility in our experiments (Figure S2). *De novo* assembly and analysis of the *foxd3*-mutant transcriptomes revealed the presence of truncated foxd3 fluorescent fusion transcripts (Figure S3A), encoding only 93 N-terminal amino acids, as shown previously (Hochgreb-Hägele and Bronner, 2013). The truncated N-terminal foxd3 variants are non-functional (Yaklichkin et al., 2007), whereas dominant negative activity is associated with the C terminus regions (Kubic et al., 2015; Zhu et al., 2014). Utilizing these lines, we investigated transcriptional changes in the absence of the functional foxd3 protein at three key stages of neural crest ontogeny (75% epiboly, 5–6ss, and 14–16ss) (Figure 2A).

At 75% epiboly, differential expression analysis between *foxd3*-mutant (CC) and control samples (C) yielded comparable numbers of upregulated and downregulated genes. In contrast at 5–6ss and 14–16ss, a larger number of putatively repressed foxd3 targets (or upregulated genes) was observed (Figures 2C and 2D), suggesting a possible change between activator and repressor roles of foxd3 during NC ontogeny. Sets of upregulated and downregulated genes were distinct at different stages, with some level of overlap between 5–6ss and 14–16ss, in particular among the genes de-repressed in *foxd3* mutants (Figures 2E and 2F).

FoxD3 is required for maintenance of the multipotent NC progenitor pool and, at later stages, for control of distinct NC lineage decisions, mostly by repressing mesenchymal and promoting neuronal derivatives (Dottori et al., 2001; Kos et al., 2001; Lister et al., 2006; Montero-Balaguer et al., 2006; Mundell and Labosky, 2011; Stewart et al., 2006; Teng et al., 2008). Examination of gene ontology (GO) terms overrepresented in differentially regulated genes indicated that at 75% epiboly, foxd3 appears to repress cell metabolism pathways, in particular ribosome biogenesis, RNA processing, and translation genes, as well as to negatively control genes involved in progenitor adhesion and migration (e.g., *nrp2a*, *nrp1b*, *slit1a*; $^*p < 0.05$; Figures 2G and 2H), while at the same time priming genes involved in tissue-specific programs (*gata2a*, *gata5*, *ets1*, *six1a/b*, *tfap2a/c*, etc.) (Figures 2G and 2H). Strikingly at 5–6ss, we found *foxd3*-mutant cells (CC) downregulated a large proportion of known NC genes distributed across all defined NC-GRN modules ($^{**}p < 0.01$; Figures 2G and 2H), many of which were *bona fide* NC transcription factors (~40%) and signaling or cell junction and adhesion molecules (~25%) (Figures 2G and S3B). Some factors previously reported to act upstream of *foxd3*, such as *prdm1* and *tfap2a/c* (Li and Cornell, 2007; Powell et al., 2013; Sauka-Spengler and Bronner-Fraser, 2008), were downregulated (Figure 2G), challenging proposed epistatic relationships within the NC-GRN. Statistical overrepresentation of the entire set of genes downregulated at 5–6ss yielded highly significant association with neural crest and stem cell development GO terms as well as terms linked to onset of EMT, cell adhesion changes, and NC cell migration (Bonferroni; $^{**}p < 0.01$; Figure 2H). Interestingly, enriched terms also linked to NC derivative fates (pigment cells, cranial skeletal development, and autonomic and enteric nervous system). However, this enrichment was correlated to the defect in expression of the core NC TFs

(B) Lateral view of a *foxd3*-mutant embryo expressing both Citrine and mCherry instead of foxd3 in premigratory NC.

(C and D) (C) Bar plot comparing numbers of differentially expressed genes in *foxd3*-mutant and control NC and (D) violin plots comparing fold-change differences.

(E and F) Venn diagrams comparing upregulated (E) and downregulated (F) genes in *foxd3*-mutant cells.

(G) Heatmap showing fold change in expression of known NC genes between *foxd3*-mutant and control cells at 75% epiboly, 5–6ss, and 14ss. Genes are grouped to reflect NC-GRN structure.

(H) Bubble plot summarizing enrichment and ps (Benjamini-Hochberg corrected) for the most significant biological process GO terms associated to differentially expressed genes.

(I) In situ hybridization of 3–6ss zebrafish embryos (dorsal view) showing decrease or loss in expression of NC specifier genes in *foxd3*-mutants.

(J) Bar plot representing fold change in expression of NC factors showing that paralogs are differentially regulated by foxd3.

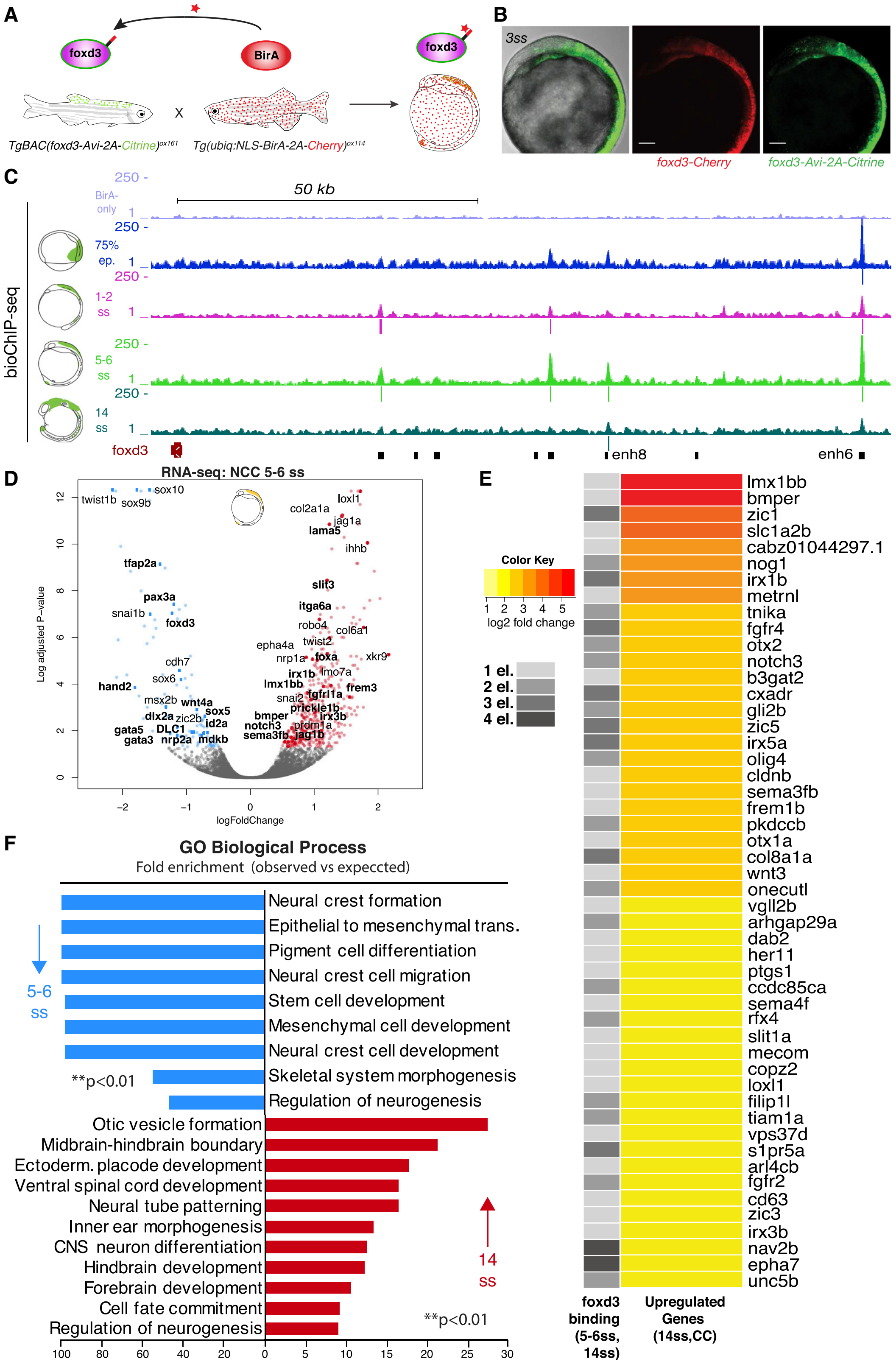

Figure 3. Biotin-ChIP Analysis Supports a Direct Bi-modal foxd3 Regulatory Action on NC Gene Expression

(A) Experimental strategy for biotagging foxd3 protein *in vivo*. Zebrafish transgenics expressing Avi-tagged *foxd3* and ubiquitous NLS-BirA are crossed to obtain embryos expressing biotinylated foxd3 for use in biotin ChIP-seq.

(legend continued on next page)

(*pax3a*, *sox9b*, *sox10*, *tfap2a*, etc.) that act both in NC specification and later in NC differentiation rather than to the loss of NC downstream differentiation markers proper, which were unaffected at premigratory stage (5–6ss) (Figure 2G). Downregulation of NC specifiers was confirmed by in situ hybridization (Figure 2I).

Analysis of *foxd3* mutant cells (CC) at migratory NC stages (14–16ss) showed dysregulation of NPB and derivative markers (Figure 2G). Surprisingly, in migrating *foxd3*-mutant NC, we observed an untimely upregulation of markers of ectomesenchymal derivatives (*lmx1ba/b*, *bmp5/6*, *col2a1b*) and neuronal lineages (*delta b/d*, *robo4*, *slit1a/b*, *slit2/3*), but only partially of melanophore, xanthophore (*isl1*, *kita1*, *pmela/b*, *tyrp1b*, *ascl1a*), and glial lineages (*gfap*, *olig2/4*, *gfra1b*, *myt1a/b*, *plp1*, *slc1a3b*, *bhlhe23*), which normally would be expressed much later or not expressed in *foxd3*+ NC derivatives (Figure 2G). Notably, two other characterized zebrafish *foxd3* mutants, mother superior (*mos*) (Montero-Balaguer et al., 2006) and sympathetic (*sym1*) (Stewart et al., 2006), showed cranio-facial defects at later stages of development (~3 dpf [days post fertilisation]) affecting branchial arches while *sym1* mutants were also lacking sympathetic neurons. Our observed mis-expression of differential markers at 14–16ss that were expected to be expressed at later stages (~20ss) suggests a likely dysregulation of differentiation of daughter cell types at stages prior to that at which the phenotype is observed.

Several derivative and ectomesenchymal markers (*col2a1a/b*, *lmx1bb/b*) and cell surface signaling molecules (*epha4a*, *slit2/3*, *robo4*), were already de-repressed at the premigratory stage (Figure 2G), in line with a role of foxd3 in preventing premature differentiation into NC derivatives. Statistical overrepresentation tests associated the upregulated gene sets to multiple GO terms reflecting biological processes essential for NC migration (cell migration and adhesion), suggesting a possible role of foxd3 in active repression mesenchymal and migrating programs at this stage (Figures 2G and 2H). Interestingly, a number of derivative markers associated with late NC differentiation (neurogenesis, axonogenesis), not expressed above background (> 1FPKM) in *foxd3*+ control cells (C) at this stage, were upregulated (de-repressed) in *foxd3*-mutant cells (CC), suggesting a continuous repressive role of foxd3, possibly ensuring commitment to specific NC lineages.

To assess whether the foxd3 mutant cells retain their NC identity, we performed comparative differential expression analysis of *foxd3*-control (C) and *foxd3*-mutant (CC) cells versus the corresponding foxd3-negative embryonic cells. Examination of their transcriptional signature shows that *foxd3*-mutant cells retain a mesenchymal NC-like phenotype and have distinct signatures from the other cells in their environment (Figure S3C). This is consistent with extensive phenotypic analysis of *foxd3*-mutants demonstrating that they exhibit defects in formation of the full complement on NC derivatives (Hochgreb-Hägele and Bronner, 2013).

Interestingly, several paralogs belonging to the same gene family were differentially regulated in the mutant cells. For instance, key NC factors (*snai1b*, *twist1b*, etc.) were downregulated, while *snai2* and *twist2* were upregulated (Figure 2J), offering a possible mechanism for rescue of *foxd3* transcriptional phenotype by paralogous genes (Marletaz et al., 2015). Additionally, several Fox transcription factors were upregulated in *foxd3* mutants, which suggests a different, upstream compensating mechanism by different Fox family members.

Altogether, these results show that foxd3 may play different regulatory roles depending on the temporal context. Importantly, in the absence of a functional foxd3 protein, much of the NC specification module is absent at 5–6ss. We also find unexpected other Fox proteins and alternative NC factor upregulation that suggests a potential compensation in the mutant and explains a partial rescue of NC specification by early delaminating NC stages (Figures 2J and S3D). While genes associated with NC and stem cell processes are downregulated in the mutant premigratory NC, genes governing migration and differentiation are upregulated at migratory stages (Figure 2H), suggesting that foxd3 switches from an activator to a repressor of NC programs.

Biotin-ChIP Confirms Direct Bimodal Action of foxd3 on the NC Gene Regulation

To further investigate the apparent bimodal function of foxd3 in gene regulation throughout NC ontogeny, we interrogated the genome-wide dynamics of direct foxd3 binding from early steps of NC induction (75% epiboly, 1–2ss) and specification (5–6ss) to migratory NC stages (14ss). To this end, we used our recently developed binary biotagging approach (Trinh et al., 2017), enabling specific biotinylation of target proteins *in vivo* for subsequent use in biochemical procedures (Figure 3A). The effector transgenic zebrafish line, *TgBAC(foxd3-Avi-2A-Citrine)*^{ox161}, expressing Avi-tagged foxd3 protein in an endogenous fashion (Figure 3B), was crossed to the ubiquitous BirA driver, *Tg(ubiq:NLS-BirA-2A-Cherry)*^{ox114}, expressing the biotin ligase, BirA, targeted to the nucleus (Figure 3A). The resulting progeny was collected for biotin ChIP-seq (Figure 3C), with BirA-only expressing embryos used as control.

Biotin ChIP-seq revealed 624 foxd3-bound regions at 75% epiboly, 531 at 1–2ss NC, 2,955 at 5–6ss NC and 658 at 14ss

(B) Lateral view of the embryo issued from crosses of *TgBAC(foxd3-Avi-2A-Citrine)*^{ox161} and *Gt(foxd3-mCherry)*^{ct110R} shows overlap of Citrine and Cherry reporters. Scale bars correspond to 100 μm.

(C) Genome browser screenshot showing mapped foxd3-biotin-ChIP-seq at 75% epiboly (in blue), 1–2ss (in pink), 5–6ss (in light green) and 14ss (in dark green) developmental zebrafish stages within the *foxd3* regulatory locus. BirA-only ChIP-seq control at 5–6ss is shown in purple (top track). Positions of called peaks are indicated as vertical lines underneath each biotin-foxd3 ChIP track. The bottom track black boxes display identified *cis*-regulatory elements of the *foxd3* gene.

(D) Volcano plot highlighting that most NC specifiers are downregulated in *foxd3*-mutant NC at 5–6ss. On the left side (downregulated genes), genes directly bound by foxd3 at 1–2ss are marked in bold. On the right side (upregulated genes), genes that are still upregulated at 14ss and are directly bound by foxd3 at 5–6ss and 14ss are marked in bold.

(E) Heatmap displaying top 50 most upregulated genes, based on $\log_2$-fold change of differential gene expression, out of total 223 genes, at 14ss in *foxd3* mutant (CC) NC that were found to be occupied by foxd3 at 1, 2, 3, or 4 associated *cis*-regulatory elements at 5–6ss and 14ss.

(F) Bar plot showing GO terms significantly enriched (**$p < 0.01$) to downregulated genes at 5–6ss in blue and upregulated genes at 14ss in red in *foxd3*-mutant embryos that were bound by foxd3 at 1–2ss and at 5–6ss/14ss, respectively.

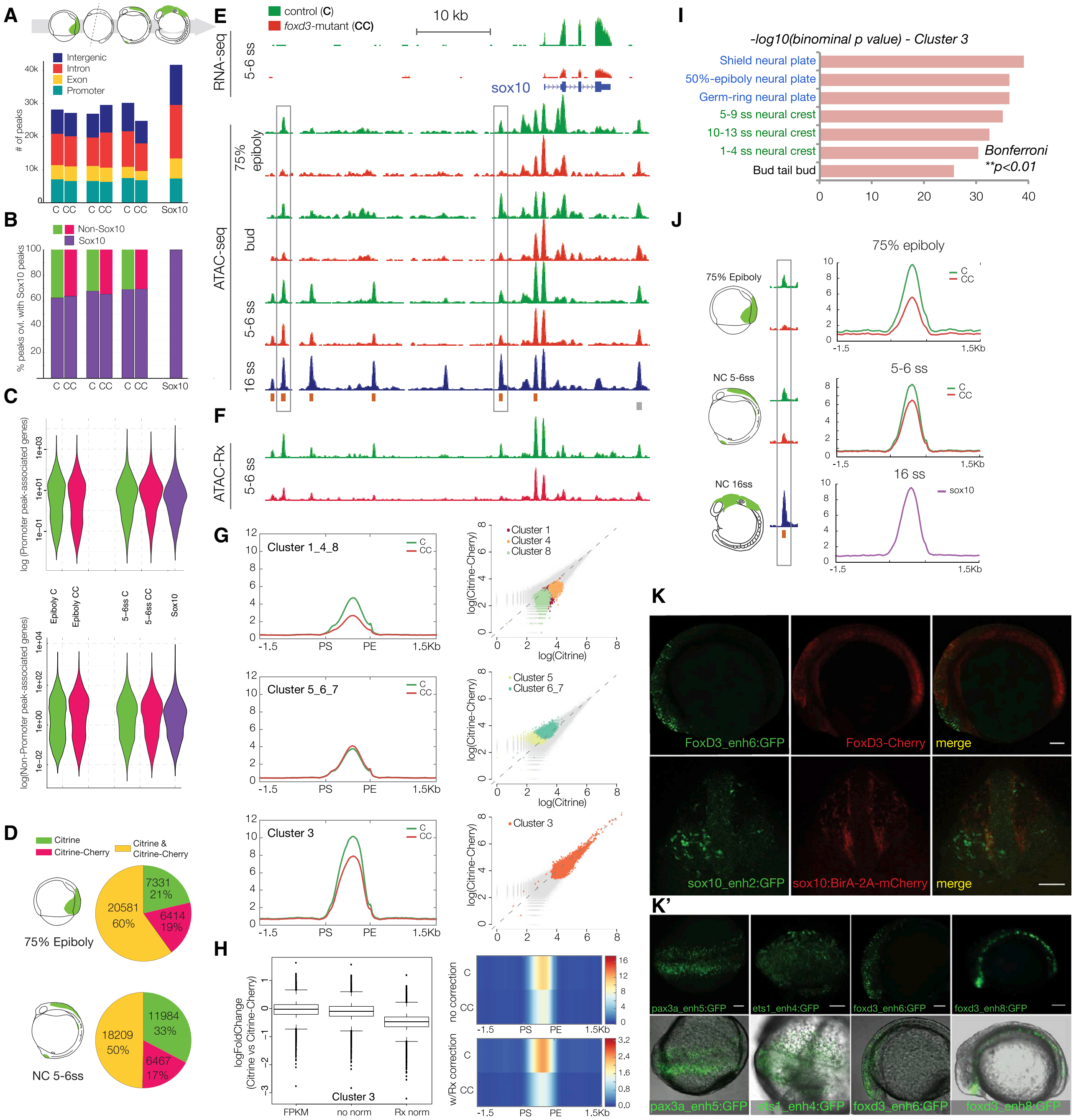

Figure 4. Epigenomic Profiling of Chromatin Accessibility in *foxd3* Mutant NC across Developmental Time

(A and B) (A) Stacked bar plots depicting genomic annotation of ATAC-seq peaks across stages analyzed (75% epiboly; bud stage; 5–6ss and 14–16ss) and (B) quantification of open elements at earlier stages as a proportion of accessible elements detected in migrating/differentiating NC.

(C) Violin plots correlating putative promoter and *cis*-regulatory elements with gene expression levels. Bimodal distribution of gene expression is associated with putative enhancers at all stages, but with promoters only at epiboly.

(D) Pie charts comparing Citrine-only, Cherry-only, and Citrine/Cherry peak number proportions of ATAC-peaks.

(E) Genome browser screenshot showing RNA-seq and ATAC-seq profiles in *foxd3* mutant (red) and *foxd3*-control cells (green) within *sox10* locus.

(F) Tracks showing normalized ATAC-Rx profiles obtained using reference exogenous *Drosophila* epigenome.

(G) Mean density maps of merged profiles and corresponding scatterplots of raw counts for *k*-means clusters featuring elements with differential accessibility and signal levels in *foxd3*-mutant and controls at 5–6ss.

(H) Boxplots and heatmap (raw read counts) showing fold change in accessibility and comparing ATAC signal levels between control (C) and mutants (CC) *k*-cluster 3 elements with and without Rx normalization.

(legend continued on next page)

NC, with only 89 non-specific peaks identified in the BirA-only controls. The substantial increase in foxd3-occupied genomic loci at 5–6ss followed by a drop in the peak number at 14ss suggests that the 5–6 somite stage represents a highly dynamic interface stage encompassing both activating and repressive modes of foxd3 action.

We next sought to distinguish genes that are either directly activated or repressed by foxd3. To this end, we annotated each NC foxd3-biotin ChIP-seq peak to the nearest expressed gene at the corresponding or later stage. We found that 14.3% of genes downregulated in the 5–6ss *foxd3*-mutant NC (Figure 3D) and 30.8% of genes upregulated in the 14ss *foxd3*-mutant NC were normally directly bound by foxd3. Notably, 61 out of 223 direct foxd3 target genes (at 5–6ss and 14ss) were found upregulated in *foxd3* mutants starting from 5–6ss (Figure 3D). This further supports our hypothesis that foxd3-mediated activation of later NC factors and foxd3 repression of those no longer used co-occur at the premigratory NC stage.

Statistical overrepresentation of foxd3-primed, directly controlled genes, downregulated in *foxd3*-mutant at 5–6ss (Figure 3D) revealed a significant association with neural crest, stem cell, and mesenchymal cell development, NC cell migration, and regulation of neurogenesis (**$p < 0.01$; Figure 3F). Important genes in NC development, such as *pax3a*, *tfap2a*, *nrp2a*, and *foxd3* itself, appeared to be positively regulated by the upstream action of foxd3 at early premigratory NC stages. Similarly, expression of transcription factors *id2a* and *gata3*, a signaling molecule *wnt4a* and a cytokine *mdkb* (all implicated in NC neuronal lineages), also appeared to be activated by foxd3 (Figure 3D). Conversely, by 14ss stage, foxd3-facilitated gene repression was directed at various genes involved in cell fate commitment, including *olig2*, *tfap2c*, and *hey2*, wnt signaling genes (e.g., *wnt3*), and neuronal differentiation (e.g., *slit2/3*, *neurod4*, *gli2b*, *otx2*, and *efna1b*; Figures 3E and 3F).

Cumulatively, our foxd3 biotin ChIP-seq data in premigratory and migratory NC argue for direct activation of a large portion of NC specification genes, followed by direct repression of cell differentiation genes, particularly to prevent premature differentiation into neuronal lineages.

foxd3 Affects Chromatin Accessibility of Distal *cis*-Regulatory Elements

The counter-intuitive finding that a large number of NC specification factors (Figures 2G and 3D) were downregulated in *foxd3*-mutant at 5–6ss raises the intriguing possibility that, much like FoxA1/A2 factors during endodermal specification (Iwafuchi-Doi et al., 2016), FoxD3 may act as a pioneer factor during NC specification. Therefore, in addition to its described role as a transcriptional repressor (Xu et al., 2007; Xu et al., 2009), FoxD3 may modulate the local epigenetic state of multiple *cis*-regulatory elements and thus positively control NC genes. To assess chromatin accessibility status in *foxd3*-mutant NC cells, we carried out cell-type specific assay for transposase-accessible chromatin using sequencing (ATAC-seq) at different stages of NC formation on either FAC-sorted *foxd3*-expressing (C) and *foxd3*-mutant NC cells (CC) (75% epiboly and 5–6ss) or on dissected *foxd3*-mutant and control anterior embryonic cells at 1–2ss. In addition, we used our previously published 16ss *sox10*-specific ATAC-seq (Trinh et al., 2017), containing an extensive cohort of open *cis*-regulatory elements in migratory NC.

We recovered a constant number of open chromatin regions (ATAC-seq peaks) at all early stages with a similar genomic distribution as distal (intronic, intergenic) or proximal (promoter). The dramatic increase in the total number of open elements in late migratory and differentiating NC cells was entirely accounted for by novel distal non-promoter elements (Figure 4A). The foxd3 depletion did not affect the distribution of peaks according to genomic annotation ($p = 0.8743$ and 0.614 for epiboly and 5–6ss, respectively), and over 60% of total peaks observed in *sox10*-specific differentiated cells were already opened at earlier stages (Figures 4B and 4E). To verify whether the open chromatin state of promoters and distal *cis*-regulatory elements correlates with gene expression, we analyzed the transcription levels of the closest associated genes. We noted a bimodal distribution of gene expression levels associated with putative enhancer elements at all stages but with putative promoters only at epiboly. Unimodal distributions after epiboly for genes associated with putative promoters indicated an onset of the *cis*-regulatory role for foxd3 at 5–6ss (Figure 4C). Moreover, while at 75% epiboly, the difference in number of unique peaks in control (C) and cells is negligible (21% versus 19%), the number of peaks in control cells at 5–6ss is almost 2-fold of that in mutants (33% versus 17%) (Figure 4D).

To investigate whether the accessibility dynamics of distal regulatory elements could account for the drastic depletion of NC specification genes at 5–6ss, we compared the ATAC-seq profiles in *foxd3*-mutant (CC) and *foxd3*-control cells (C) (Figure 4E). *K*-means clustering identified 8 cohesive groups of elements with 3 general trends: (1) *k*-clusters 1, 4, and 8 contained lower signal elements with prominent accessibility differences between mutant and controls (C >> CC), (2) *k*-clusters 5, 6, and 7 comprised elements of equally low comparable accessibility (C ≈ CC), and (3) *k*-cluster 3 contained highly accessible regions with broad ATAC-seq peak distribution that showed intermediate signal decrease in mutants (C > CC) (Figure 4G). Functional annotation of *k*-clusters using GREAT Tool (McLean et al., 2010) singled out two clusters reflecting NC regulatory mechanisms—*k*-clusters 3 and 4 showed specific enrichment of zebrafish gene expression ontology terms linked to NC and neural plate development (Bonferroni; **$p < 0.01$; Figures 4I and S4A).

To quantify the observed difference in ATAC-seq signal, we adapted a ChIP-Rx method (Orlando et al., 2014) that enables

(I) Bar chart depicting functional annotation of *k*-cluster 3 shows enrichment in zebrafish gene expression ontology terms linked to NC and neural plate development (Bonferroni; **$p < 0.01$). For further analysis of *k*-cluster, see Figure S4.

(J) Merged profiles for 3,565 elements open at 75% epiboly showed more prominent accessibility defect than at 5–6ss (C >> CC, > 50%), suggesting biological compensation over time.

(K and K′) *Cis*-regulatory elements from *k*-cluster 3 show NC-specific reporter activity. (K) Lateral and frontal view of embryos injected with *foxd3*-enh6 and *sox10*-enh2 GFP reporter constructs into the genetic background of *foxd3-Cherry* and *sox10:BirA-2A-Cherry*, respectively. Scale bars correspond to 100 μm. (K′) Fluorescent and bright-field overlay images of *pax3a* and *ets1* (dorsal view) and foxd3 (lateral view) enhancers. Scale bars correspond to 100 μm.

genome-wide quantitative comparative analysis of histone modification ChIP signal (ATAC-Rx). To this end, ATAC was performed on mutant (CC) and control (C) *foxd3*-expressing NC cells at 5–6ss, spiked with *Drosophila melanogaster* S2 cells as a reference exogenous epigenome (Figure 4F). Quantification after Rx normalization demonstrated a discernible fold-change difference in accessibility between control (C) and mutant (CC) elements (Figure 4H), thus further confirming the defect in opening of specific distal *cis*-regulatory elements in the *foxd3*-mutant, previously identified by *k*-means clustering.

To investigate dynamics of chromatin opening over developmental time, we performed *k*-means clustering of the 75% epiboly and bud stage ATAC data. We found a subset of *k*-cluster 3 elements was open at 75% epiboly (~20%; 3,565 el. [elements]), with a more prominent change in enhancer accessibility in *foxd3* mutants at this stage (C >> CC; > 50%) as compared to 5–6ss (Figure 4J), suggesting the defect in foxd3 mutants is compensated over time.

Using an efficient reporter assay in zebrafish, we tested the activity of ~30 putative regulatory elements from *k*-clusters 3 and 4. *k*-cluster 4 regions were not active at 5–6ss but perhaps are used at later stages, to maintain NC specifiers that remained downregulated in 14–16ss *foxd3* mutants. *k*-cluster 3 elements drove reporter expression at 5–6ss with striking NC-specific activity, recapitulating endogenous expression of their cognate genes (Figures 4K and 4K′), thus strongly suggesting they act as their *cis*-regulatory elements.

Hotspot Enhancers Associated with Downregulated NC Specification Genes Harbor Specific NC Regulatory Code

k-cluster 3 included elements involved in both neural and NC development (Figure 4I). However, *foxd3*-mutants presented defects only in NC formation, suggesting that neural *cis*-regulatory modules may not require *foxd3* activity for proper function. Further *k*-means clustering of *k*-cluster 3 revealed two pooled subgroups that were generated by assembling subclusters that exhibited similar accessibility characteristics (Figures 5A–5C): (1) *k*-cluster 3.1 containing *cis*-regulatory elements that displayed lower accessibility in *foxd3* mutants and (2) *k*-cluster 3.2 containing elements with no accessibility change. GREAT analysis further functionally segregated these subclusters: *k*-cluster 3.2 was associated with ontology terms linked only to neural plate and tube development while *k*-cluster 3.1 contained enhancers implicated in NC specification or neuronal differentiation (Bonferroni; $^{**}p < 0.01$; Figures 5D and 5F). From henceforth, we refer to putative elements in *k*-cluster 3.1 as ‘‘hotspot enhancers.’’

To link the putative regulatory elements identified in *foxd3*-mutant (CC) and control (C) NC cells to their transcriptional programs, we first assigned all identified non-promoter ATAC-seq elements to the genes expressed at each corresponding stage (Figure S4C). To connect the transcriptional and regulatory *foxd3* phenotypes at 5–6ss, we assigned hotspot enhancers (*k*-cluster 3.1) and elements from *k*-cluster 3.2 to the corresponding genes expressed at this stage and ranked those genes by the number of elements associated (Figures 5B and 5C). Hotspot enhancers correlated to the ensemble of NC specification genes downregulated at 5–6ss with high statistical significance ($^{****}p = 1.12E-60$). Moreover, no other *k*-cluster, including 3.2, showed significant association to genes either up- or downregulated in the *foxd3*-mutant at 5–6ss.

A number of NC specifiers that were downregulated in *foxd3*-mutants at 5–6ss recovered their expression by 14ss. We inquired whether *k*-cluster 3.2 regulatory elements (unaffected by loss of foxd3) could act instead of hotspot *k*-cluster 3.1 enhancers to rescue cognate gene expression. However, the genes controlled by both hotspot enhancers and *k*-cluster 3.2 elements (Figure 5C′) compared to those controlled solely by hotspot elements (Figure 5B′) did not recover more efficiently (50% versus 40% of genes, respectively, were still depleted in *foxd3*-mutants at 14ss). Instead, an important fraction (~25%) of downregulated NC specifiers harboring 3.2 elements, were, in fact, upregulated in 14ss *foxd3*-mutant NC, and such upregulation was not observed for genes solely controlled by hotspot activating enhancers. Moreover, genes differentially upregulated at 14ss associated to *k*-cluster 3.2 with high statistical significance ($p = 4.73E-74$), suggesting that *k*-cluster 3.2 elements, were in fact linked to foxd3-mediated repression.

In line with their predicted assigned functions, transcription factor binding site (TFBS) analysis using Homer suite (Heinz et al., 2010) revealed that *k*-cluster 3.1 (hotspot enhancers) and *k*-cluster 3.2 elements harbored distinct regulatory codes. Hotspot enhancers presented a canonical neural crest signature featuring *bona fide* NC master regulators Sox10 (Sauka-Spengler and Bronner-Fraser, 2008), TFAP2a, and nuclear receptor NR2 (Rada-Iglesias et al., 2012) as top enriched binding motifs (Figure 5E), while *k*-cluster 3.2 top enriched motifs were Lhx2/3, a transcription factor involved in neural development and cortical neurogenesis (Bery et al., 2016), Oct4-Sox2, and multiple FoxA motifs (Figure 5G). Interestingly, the only other *k*-clusters that were enriched in NC motifs (TFAP2a and Ets1, but not Sox10) were *k*-clusters 1, 4, and 8 (Figure S4B), suggesting that regulatory elements whose opening is dependent on foxd3 display unifying features of an NC enhancer. Furthermore, we also found a number (~10%) of hotspot and *k*-cluster 3.2 elements were directly bound by foxd3 at premigratory stages (Figure 5H). Given the paucity of available zebrafish TFBSs, we also formulated a new approach to build comprehensive TF binding motif maps for each enhancer *k*-cluster to be used in statistical enrichment analyses. Similarly, the majority of NC TF motifs were present in the hotspot *k*-cluster 3.1 neural crest enhancers (Figure 5I), while *k*-cluster 3.2 elements clearly lacked sox10, nr2f, and most pax motifs, except for a single pax cluster, comprising human TF binding motifs for Pax3 and Pax7, previously shown to control both NC, neuronal and mesenchymal derivatives (Manderfield et al., 2014; Murdoch et al., 2012). Moreover, *k*-cluster 3.2 enhancers harbored the majority of hnf, tcf, klf, zic, and pou motifs, suggesting these elements could both drive NC derivative as well as stem cell maintenance programs at later stages of NC development and mediate repressive activity.

This analysis singled out *k*-cluster 3.1 as the *bona fide* NC enhancer cluster that contained hotspot *cis*-regulatory modules driving NC specification genes at premigratory stages. Defects in the chromatin accessibility of these hotspot enhancers resulted in the decrease of NC specifiers' expression in *foxd3* mutants.

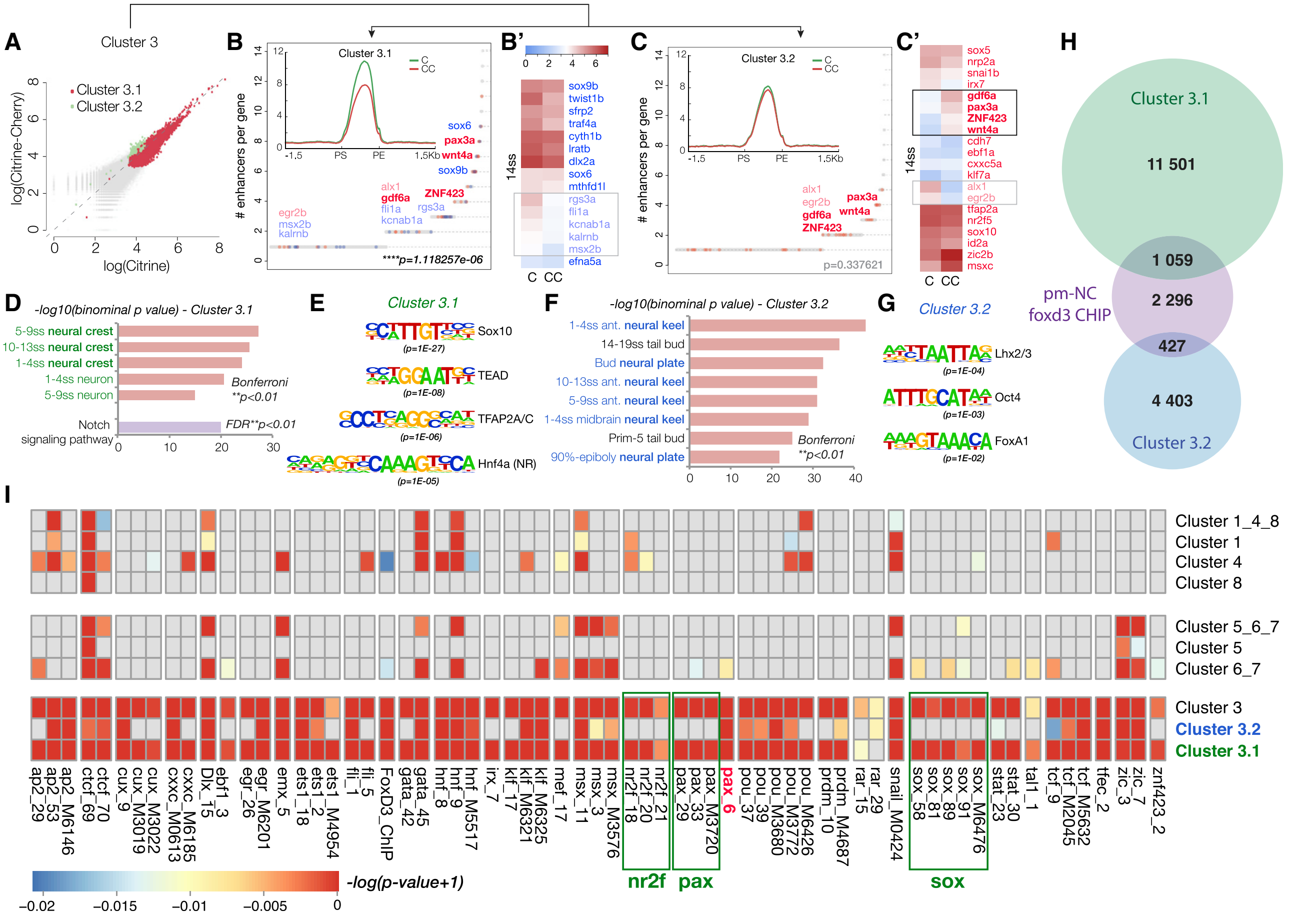

Figure 5. Characterization of Hotspot Enhancers

(A) Scatterplot showing subclustering of *k*-cluster 3, one containing elements of lower accessibility in *foxd3* mutants (*k*-cluster 3.1; 12,366 el.; $R_{Cl3.1} = 0.77$) (B) and the other elements with no change in chromatin accessibility (*k*-cluster 3.2; 4,754 el.; $R_{Cl3.2} = 0.97$) (C).

(B and C) Plots representing genes assigned to *k*-cluster 3.1 (B) and *k*-cluster 3.2 (C) ranked by the number of associated elements. (B′ and C′) Heatmaps showing later expression (14ss) of NC genes depleted in 5–6ss mutant NC. Genes controlled solely by 3.1 elements (in blue) are shown in (B′) and those harboring both 3.1 and 3.2 elements (in red) are depicted in (C′). Genes that remain downregulated at 14ss are labeled in light color print, and those overexpressed are shown in bold.

(D and F) Functional annotation by GREAT associates *k*-cluster 3.1 with neural crest specification or neuronal differentiation (D) and *k*-cluster 3.2 with neural plate/tube development (F) (Bonferroni; **p < 0.01).

(E and G) Top transcription factor binding site (TFBS) motifs enriched in 3.1 (E) and 3.2 (G) elements.

(H) Venn diagrams showing a number of elements from *k*-clusters 3.1 (in green) and 3.2 (in blue) that are directly bound by foxd3 at premigratory NC (pm-NC) stages (in purple: 75% epiboly, 1–2ss, and 5–6ss ChIP-seq peaks).

(I) Comprehensive TF binding motif map representing significantly enriched TFBS for TF expressed at 5–6ss across different *k*-clusters.

foxd3 Primes Late Regulatory Elements Used in Migratory NC

To quantitatively evaluate events of chromatin opening at 5–6ss, we performed differential accessibility analysis using the DiffBind package (Stark and Brown, 2011). We identified 900 peaks that were differentially accessible in *foxd3*-control (C) versus *foxd3*-mutant (CC) neural crest (Figures 6A–6C); these elements exhibited low signal at 75% epiboly, only starting to open at 5–6ss, but were clearly accessible in the NC at 16ss (Figure 6D). Functional annotation of identified elements revealed significant enrichment of GO terms for stem cell development and differentiation, neural crest differentiation and migration, and mesenchymal cell differentiation (**p < 0.01), as well as gliogenesis (*p < 0.05), further suggesting these regions may act as *cis*-regulatory elements at later stages of NC ontogeny (Figure 6E). Interestingly, assigned genes included cell adhesion and migration factors that were de-repressed in *foxd3* mutant NC at later stages. Conversely, other associated NC regulatory factors that drive specific NC lineages and are normally highly expressed at later stages were depleted in the *foxd3* mutant at 14ss (Figure 6A). These results clearly suggest that, in addition to the NC specification program at premigratory NC stages, foxd3 continues to aid the opening of the *cis*-regulatory elements associated with NC differentiation, while, at the same time, negatively controlling gene expression of cell surface and migration machinery that ultimately has to be deactivated in order for cells to settle and differentiate. Importantly, association of stem cell development/differentiation genes to late NC enhancers further supports a role for foxd3 in controlling stem cell identity in the migrating and differentiating NC.

CellPress

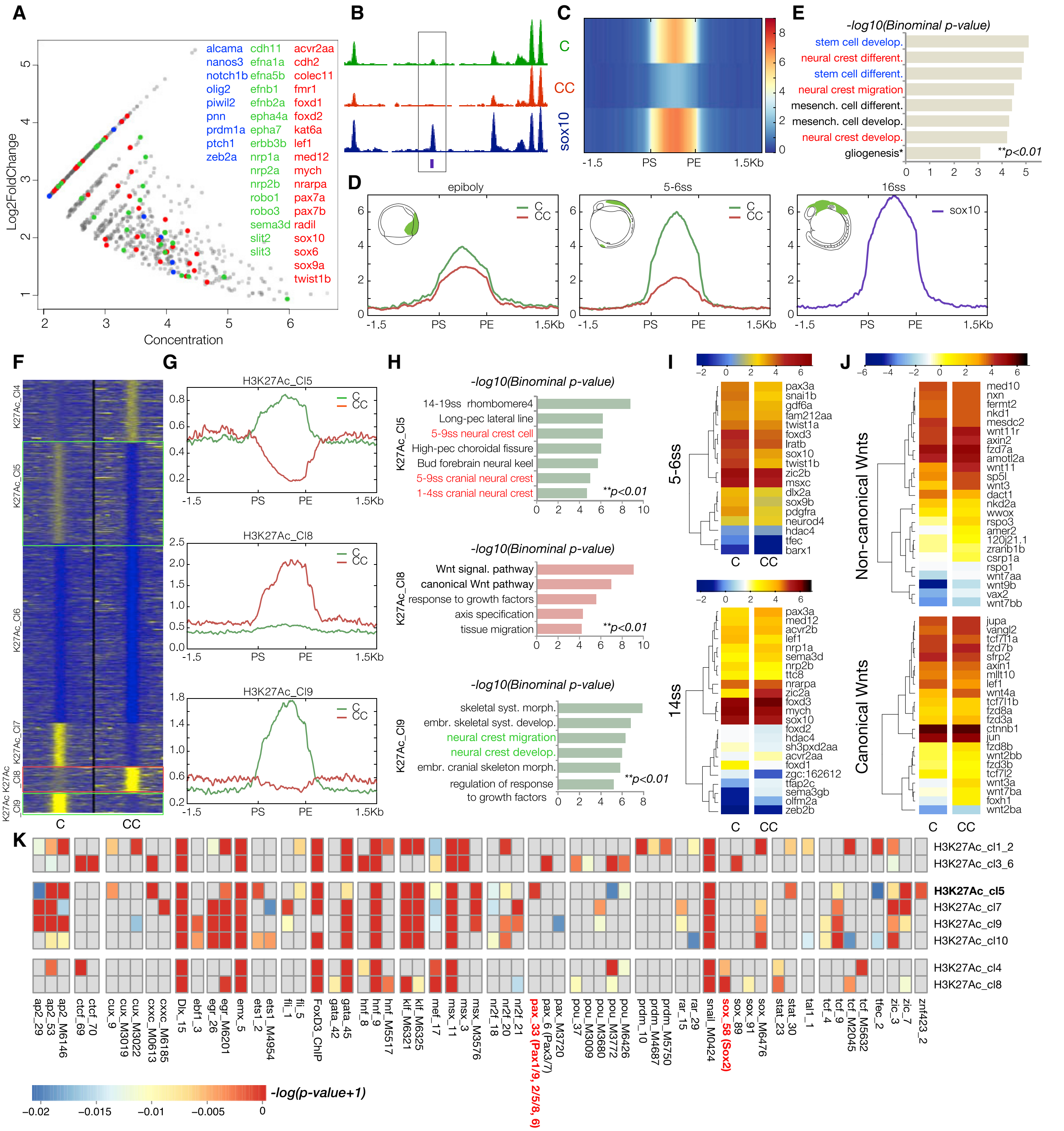

Figure 6. Differential ATAC-Seq Analysis and Clustering of Enhancers Based on H3K27Ac Profiles

(A) Annotated MA plot depicting late opening enhancers significant by DiffBind analysis ($p < 0.05$, FDR<0.1) of the ATAC-seq signal at 5–6ss with annotated associated genes (stem cell genes, blue; cell adhesion/migration cues, green; NC specification and differentiation, red).

(B) Genome browser screenshot exemplifying the type of element isolated by DiffBind (boxed).

(C and D) (C) Heatmap (raw read counts) of all elements and (D) collapsed merged profiles indicating that identified elements are closed at epiboly and only start to open at 5–6ss.

(E) Functional annotation of DiffBind-identified enhancers shows association with later roles in NC (**$p < 0.01$).

(F–H) (F) Heatmap depicting *k*-means linear enrichment clustering of H3K27Ac signal across non-promoter ATAC-seq peaks in *foxd3*-mutant (CC, Citrine/Cherry) and control (C, Citrine) at 5–6ss, (G) associated mean merged profiles for selected clusters, and (H) corresponding ontology enrichment bar plots indicating functional role of selected clusters.

(legend continued on next page)

Taken together, our findings demonstrate that foxd3 controls NC gene activation by acting at a *cis*-regulatory level both during early NC specification and at later migratory NC stages. This realization contributes to converging evidence that foxd3 plays multiple, sometimes opposing, roles, particularly during the transition from NC specification to migration/differentiation stages.

H3K27Ac on NC Enhancers Is Altered in foxd3 Mutants

To examine whether H3K27 acetylation, a hallmark of active enhancers, was affected in *foxd3*-mutant NC at 5–6ss, we carried out H3K27Ac ChIP using FAC-sorted *foxd3*-mutant (CC) and control *foxd3*-expressing NC cells (C). *k*-means clustering of H3K27Ac signal identified 10 clusters with differential patterns of H3K27 acetylation on putative *cis*-regulatory elements (Figures 6F, 6G, and S5). Four clusters (K27Ac_clusters 1, 2, 3, and 6) contained elements with no change in H3K27 acetylation, whereas four clusters showed a decrease (K27Ac_Cl5, 7, 9, and 10) and two an increase (K27Ac_Cl4 and Cl8) in H3K27Ac signal in *foxd3* mutant NC. In K27Ac_Cl5, acetylation in *foxd3*-mutants was abrogated below background levels, possibly indicating active removal of the H3K27Ac mark from the enhancers when they were not primed or bound by NC-specific TFs. Functional annotation of this cluster yielded specific enrichment of zebrafish GO terms linked to early (premigratory) NC, as well as nervous system development (Bonferroni; $^{**}p < 0.01$; Figure 6H). The majority of NC genes downregulated in *foxd3* mutants at 5–6ss were associated with one or more K27AC_Cl5 elements ($p = 1.59E{-}05$; Figure 6I), suggesting that some of the enhancers initially opened by foxd3 and used during early NC specification also depended on this factor for appropriate acetylation. Similarly, H3K27Ac_Cl9 elements, characterized by strong K27Ac signal in controls and defect in *foxd3*-mutant cells (Figure 6G), were mainly associated with factors regulating late NC events such as migration and differentiation into derivatives such as cranial skeletal elements (Figure 6H). Interestingly, a number of these genes were upregulated in *foxd3*-mutants by 14ss, indicating a supplementary *foxd3*-linked gene and enhancer regulatory mechanism (Figure 6I). The putative role of foxd3 in repression of these NC genes until post-migratory stages is reminiscent of the observations made in studies of Foxd3 function in germ and pluripotent stem cells (Krishnakumar et al., 2016; Respuela et al., 2016).

In contrast, the increased H3K27 acetylation in *foxd3*-mutants suggests foxd3 involvement in active removal of this histone modification from cluster K27Ac_Cl8 enhancers that control of Wnt signaling pathway components (Figures 6G and 6H). Correspondingly, both canonical Wnt signaling ligands (*Wnt1*,*3*,*3a*,*8a/b*, *10a/b*), receptors (*fzd3*,*8b*,*10*, *fzdb*, *sfrp1a*), signal transduction effectors (*apc*, *axin2*, *wntless*, *tcf3a/b*, *tcf15*), as well as non-canonical Wnt signaling ligands (*wnt4a*,*5b*, *7b*,*11*,*11r*,*16*) and signal transduction effectors (*daam1a/b*, rho, *plc*, *nfat3b*), were differentially upregulated in *foxd3*-mutants at 14ss (Figure 6J).

Interestingly, DNA motif enrichment patterns identified in individual K27Ac clusters differed from binding maps of hotspot *k*-cluster 3.1 enhancers. For instance, elements from K27Ac_Cl5 cluster, featuring complete repression in H3K27Ac signal in *foxd3*-mutants, lacked enrichment in sox, prdm, or pax3/7 motifs but harbored motifs for other pax TFs (pax1/9, pax2/5/8, pax6) and ets (erythroblast transformation specific). In general, K27Ac clusters containing elements acetylated in a *foxd3*-dependent manner (K27Ac_Cl5, 7, 9, and 10; Figures 6F and 6G) showed enrichment in tfap2, nr2f, and zic motifs, while elements from clusters K27Ac_Cl4 and 8, which may normally require foxd3 binding for maintenance of repressive state (Figures 6F and 6G), are enriched in binding motifs for neural and stem cell TF sox2. Regions of low acetylation across ATAC peaks in K27Ac_Cl3_6 are enriched in CTCF binding motifs (Figure 6K).

These results show that foxd3's effects on H3K27 acetylation of enhancers are context dependent. While correlating positively with H3K27ac deposition on enhancers of early specification and late fate commitment genes, foxd3-dependent H3K27Ac is negatively associated with expression of Wnt signaling genes.

Ectopic Expression of foxd3 Modifies the Chromatin Landscape in Early Embryos

Under some conditions, FoxD3 has been shown to auto-regulate itself (Hromas et al., 1999; Lister et al., 2006; Pohl and Knochel, 2001). Indeed, here we reveal that in *foxd3*-mutants, the transcription of truncated *foxd3* form was increased at 75% epiboly, depleted at 5–6ss and again upregulated at 14–16ss, indicating different feedback loops controlling *foxd3* expression at different stages of development. To investigate the direct action of foxd3-mediated chromatin priming and subsequent gene activation, we performed *foxd3* overexpression experiments by injecting *foxd3* mRNA into heterozygous *Gt(foxd3-mCherry)*^{ct110} embryos (Figure 7A). To assess the degree of auto-regulation upon ectopic *foxd3* expression versus control, we first quantified fluorescence intensity and the number of endogenous foxd3-mCherry cells at 50% epiboly by FACS (Figures 7B and S6A). While we did not observe an increase in fluorescent cell number, we noticed an overall increase in the fluorescence intensity when compared to control non-injected embryos, consistent with supplemental gene activation at the *foxd3-mCherry* locus. Remarkably, cells from *foxd3* mRNA-injected embryos failed to exhibit the highest mCherry fluorescence found in the control cells (Figure 7B; P5 compartment – black arrow), suggesting also a potential repression at the *foxd3-mCherry* locus. Our findings suggest that foxd3 both activates and represses itself and that its activity may be dependent on the concentration and spatial position of the cells within an embryo. Thus, under overexpression conditions, the bimodal action of foxd3 may occur at an even earlier stage than normal.

(I) Heatmaps showing expression of NC specification genes (log FPKM) associated with K27Ac_Cl5 at 5–6ss and NC migration/differentiation genes associated with K27Ac_Cl9 at 14ss in *foxd3*-mutant (CC) and control cells (C).

(J) Heatmap depicting expression at 14ss of canonical and non-canonical Wnt pathway molecules (in log FPKM) associated with K27Ac_Cl8 that displays an increase in enhancer K27 acetylation in mutants.

(K) TF binding motif map representing significantly enriched TFBS for TF expressed at 5–6ss across different K27Ac-clusters. See Figure S5 for other K27Ac clusters and corresponding ontology enrichment bar plots.

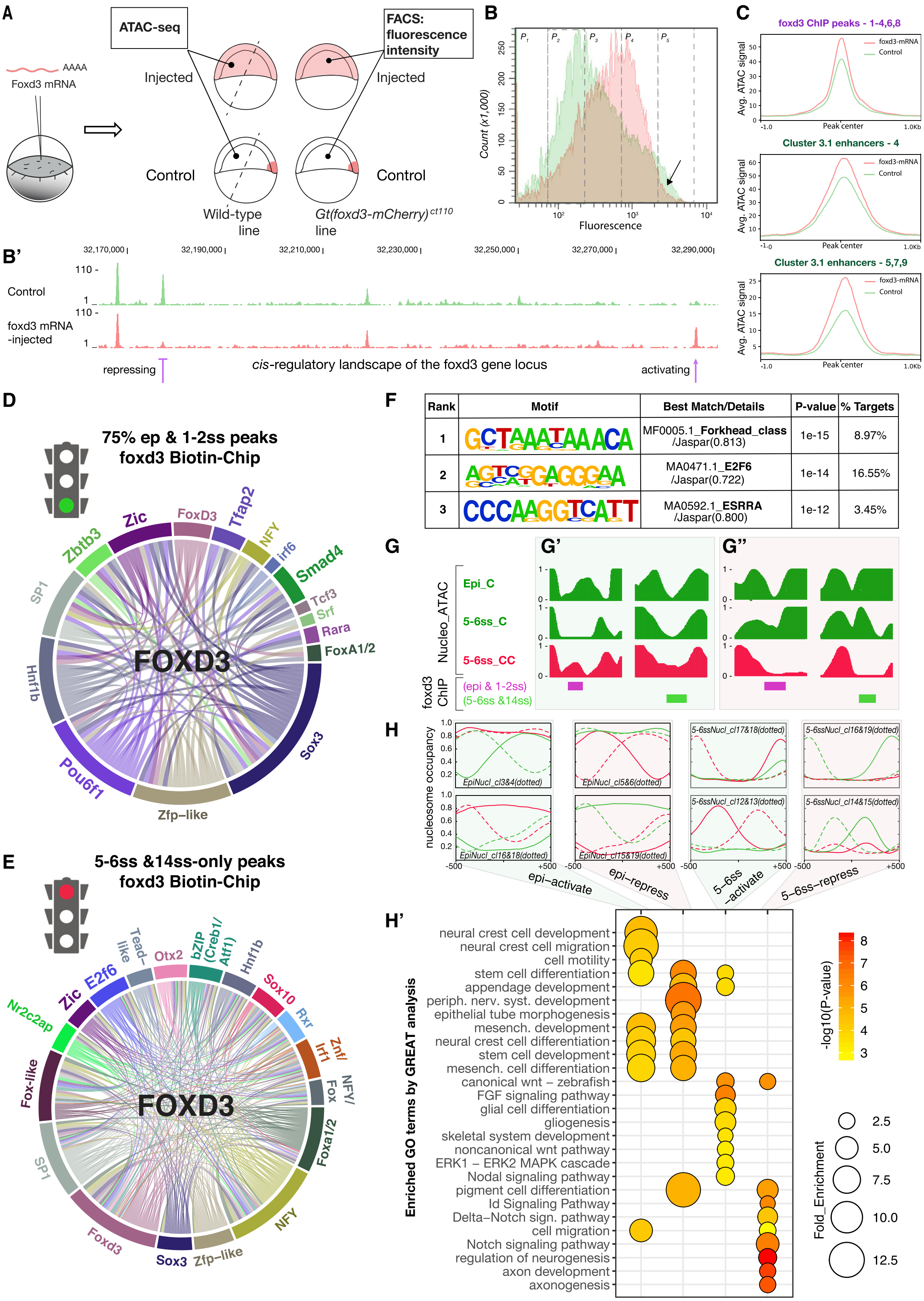

(legend on next page)

To probe foxd3's capability to prematurely prime *cis*-regulatory elements, we again overexpressed it but, this time, dissected the embryos to analyze chromatin accessibility (ATAC-seq) in cells that do not normally express foxd3 endogenously at this stage ("foxd3-naive" cells) (Figure 7A). When comparing datasets from *foxd3*-injected embryos versus controls, we failed to detect the opening of *de novo* genomic regions that were not normally accessible in the *foxd3*-expressing cells. This suggests that foxd3 activity remains specific to a defined set of putative downstream targets. Interestingly, we observed that ectopic foxd3 activity increased accessibility of a large number of putative elements directly bound by foxd3 (as uncovered in our ChIP experiments), including a number of hotspot enhancer elements (Figure 7C). Elements that showed enhanced accessibility upon ectopic *foxd3* expression associated with genes involved in early neural plate and neural crest development and in particular with those controlling Wnt and BMP signaling (Figures S6B and S6B′). Furthermore, within the *foxd3* genomic locus itself, we identified *cis*-regulatory elements showing changes in accessibility in both directions upon *foxd3* expression (Figure 7B′, purple arrows). Bimodal auto-regulation of *foxd3* (Figures 7B and 7B′) offers an excellent example of foxd3's capacity to both activate and repress a gene by priming or decommissioning different *cis*-regulatory elements.

In silico Combinatorial Transcription Factor Analysis Suggests Distinct Gene Regulatory Mechanisms Underlie the Bimodal Action of foxd3

Because late *cis*-regulatory regions were not fully opened by ectopic *foxd3* expression, we speculated that foxd3 likely requires *in vivo* interacting partners to exert its bimodal activity. To identify putative foxd3 partners, we analyzed the underlying foxd3 biotin ChIP sequences during either gene activation or gene repression. The classification of foxd3-bound regulatory elements solely using one of the bimodal actions was based on the assumption that these roles are temporally distinct, with foxd3 mainly acting as activator during early stages (75% epiboly; 1–2ss; "early" peaks), and as a repressor at later stages of NC ontogeny (5–6ss; 14ss). Given that at the 5–6ss foxd3 may mediate both activities, the set of "late" peaks was obtained by removing peaks already bound at earlier stages, thus focusing primarily on the foxd3-repressed genomic loci. After *de novo* TF binding motif enrichment analysis on early and late foxd3-bound NC genomic regions, we selected 20 out of 20 identified (early) and 27 out of 32 identified (late) significantly enriched sequence motifs to be used for *in silico* 2-way TF combinatorial analysis. For visualization purposes, different motifs annotated to the same TF were pooled (Figures 7D, 7E, S6C, and S6D). Combinatorial analysis yielded a signature of putative foxd3 co-activators at early stages (Sox3, SP1, Zic, Hnf1ba, Pou6f1, zinc-finger protein (ZFP), Zbtb3, and Smad4) (Figure 7D). Conversely, late stage peaks were enriched for NFY, SP1, Otx2, Sox10, E2F6, Rxr, bZIP, Nr2c2ap, Foxd3, and other Fox-like TF motif complexes (Figure 7E). Given that some of the lineage specification factors enriched at later stages overlap foxd3 binding genomic coordinates, we cannot unequivocally discriminate whether these factors cooperate or compete with foxd3. Surprisingly, we did not observe an extensive enrichment of fox/foxd3 motifs from our foxd3 biotin ChIP-seq sets at early NC stages (when foxd3 is mostly playing a priming role), while fox/foxd3 were the most highly enriched out of all motifs at foxd3-bound DNA sequences at later NC stages (when foxd3 primarily acts as a repressor) (Figures 7D, 7E, S6C, and S6D). This is suggestive of differences between foxd3 binding affinities during its priming versus repressive modes and is consistent with previous studies showing that some co-binding pioneer transcription factors exhibit only a partial DNA sequence motif recognition when binding to the nucleosome, but recognize canonical motifs when binding "naked" DNA (Soufi et al., 2015). Thus, our findings support the hypothesis that foxd3 is a bimodal NC regulator, which progressively changes from a co-pioneering factor toward a repressor during NC ontogeny.

Foxd3 Mediates Gene Repression via Chromatin Modulation together with Other Factors

To focus on potential factors that may directly co-repress NC genes at later stages, we scanned foxd3-occupied genomic

Figure 7. Putative Mechanisms of the Bimodal foxd3-Mediated NC Gene Regulation

(A) Experimental strategy for *foxd3* overexpression *in vivo*. *Gt(foxd3-mCherry)^{ct110}* heterozygous embryos were used for FACS and wild-type embryos for ATAC-seq experiments at 50% epiboly stages. For ATAC-seq, embryos were dissected (dashed lines) to only collect "foxd3-naive" cells that do not normally express foxd3. Native and ectopic foxd3 expression is illustrated in dark pink and lighter pink, respectively.

(B) FACS graph portraying a number of *foxd3-mCherry* expressing cells and underlying fluorescence intensities from control (green) and *foxd3* mRNA injected (pink) embryos. P1–P5 – compartments of different fluorescence levels from the lowest to the highest. Black arrow indicates a loss of highest intensity fluorescence in *foxd3* mRNA injected embryos versus control. (B′) Genome browser screenshot depicting region ~60 kb upstream from the *foxd3* transcription start site (TSS). Green and pink ATAC-seq tracks represent genome accessibility from control and *foxd3* overexpressing embryonic cells. Purple arrows indicate either relative loss or acquisition of chromatin accessibility upon *foxd3* overexpression.

(C) Mean density maps of merged profiles for *k*-means clusters featuring elements with differential accessibility between the *foxd3* mRNA injected (in pink) and control (in green) 50% epiboly-staged embryos using either foxd3 binding maps or *k*-cluster 3.1 elements as a reference.

(D) Circle plot showing statistically significant TF motif co-occurrences on the "early NC" foxd3-bound activating elements.

(E) Circle plot showing different statistically significant TF motif co-occurrences on the "late NC" foxd3-bound elements, underlying repressive activity.

(F) *De novo* TF binding motifs enriched within foxd3-bound elements associated with NC genes negatively regulated by foxd3 at 14ss.

(G) Nucleosomal occupancy profiles expressed as relative NucleoATAC normalized cross-correlation signals. Profiles show changes in nucleosome positioning within the regulatory elements in control (C; green) and *foxd3*-mutant (CC; red) cells. Direct foxd3 binding at either early (epiboly, 1–2ss in magenta) or late stage (5–6ss, 14ss in green) results in either nucleosome clearing (G′; permissive role) or nucleosome compaction (G″; repressive role). Both processes are altered and nucleosomal patterns inverted in *foxd3*-mutant NC (G′ and G″).

(H) Mean density maps of merged profiles of nucleosomal clusters obtained by *k*-means analysis showing differential nucleosomal patterns between *foxd3*-mutants (CC; red) and controls (C; green). Both nucleosome-loose clusters of elements with activating patterns (epi-activate and 5–6ss-activate) and nucleosome-compact clusters with repressive patterns (epi-repress and 5–6ss-repress) are identified. (H′) Bubble chart depicting functional annotation of different nucleosomal clusters by GREAT (Bonferroni; $p < 0.01$). Only elements directly bound by foxd3 are analyzed.

loci associated with genes significantly upregulated in the 14ss *foxd3*-mutant NC for *de novo* TF binding motifs. Interestingly, only three significantly enriched motifs were identified: foxd3 (MF0005.1_Forkhead_class/Jaspar(0.813); $p = 1E-15$), e2f6 (MA0471.1_E2F6/Jaspar(0.722), $p = 1E-14$), and Esrra (MA0592.1_ESRRA/Jaspar(0.800), $p = 1E-12$) (Figure 7F). E2F6 was found to co-occupy the highest proportion of foxd3-bound repressive peaks (16.55%) (Figure 7F).

Next, we identified a set of genomic regions accessible early (75% epiboly; 1–2ss), then bound by foxd3 from 5–6ss and ultimately closed by 14–16ss, suggesting foxd3-facilitated compaction. Such regulatory elements (314) were associated to 293 gene loci, 44% of which were upregulated in *foxd3*-mutants at 14ss, indicating these were directly regulated by foxd3 via modulation of chromatin accessibility at enhancers. Moreover, GREAT analysis revealed a significant enrichment for neuronal fate specification terms (Figure S6E′; $^{**}p < 0.01$), including genes such as *olig2/4*, *mnx1*, *fgf8a*, *gli1*, *neurog1*, and *robo1*, confirming our previous observations that foxd3 prevents premature activation of neuronal pathways (Figure 2H). TF motif enrichment analysis of these 314 elements using the same initial set of 32 motifs (Figure 7E) yielded 19 significant co-occurring motifs (12 different TFs) (Figure S6E). We again noted promiscuous foxd3 binding, as the top five different fox/foxd3 motifs were enriched on 85% of repressed loci. Interestingly, 98.1% of all repressed loci exhibited the Hnf1b a/b motif sequence, while other predominantly enriched motifs underlying foxd3-mediated co-repression were otx2 (92.7%), sox10 (79.9%), two NFY motifs (78.7% and 79.9%), and e2f6 (70.7%). Combinatorial TF analysis showed that fox motif factors had the highest number of statistically significant co-occurrences, followed by foxd3 co-occupation with sp1, zfp-like, e2f6, otx2, sox3, NFY, znf-irf1, hnf1b a/b, and sox10 motifs (Figure S6E). Otx2, sox3, and sox10 factors, involved in NC differentiation and neural development, may not co-operate with foxd3 to mediate repression but rather compete for underlying binding sequences to promote multipotent NC cell differentiation (Beby and Lamonerie, 2013; Carney et al., 2006; Dee et al., 2008). Conversely, e2f6 factor is known to function as a transcriptional repressor that associates with Polycomb repressive complexes (PRC1 and PRC2) (Gaubatz et al., 1998; Leseva et al., 2013; Trimarchi et al., 2001). We explored e2f6/foxd3 co-operation in transcriptional repression by scanning four different e2f6 motifs across all foxd3-bound regions associated with the genes upregulated in 14ss *foxd3*-mutant embryos (Figure 3E) and found that 82.3% of them were significantly enriched for e2f6 binding (chi-square test; $^{***}p < 0.0004$). This indicates that e2f6 likely plays an important role in NC development by co-operating with foxd3 to repress target genes in order to maintain NC multipotency.

Foxd3 Affects Nucleosomal Positioning on NC Enhancers

Our analysis suggests that foxd3-mediated chromatin compaction at regulatory elements is one mechanism employed for specific gene repression. However, the role of foxd3 in gene activation and maintenance may involve other mechanisms including nucleosomal rearrangements at NC enhancers. FoxD3 forkhead DNA binding domain, like that of FoxA proteins, is composed of three helices and two large loops ("wings"), remarkably similar to the winged-helix structures of linker histone H1 that avidly binds nucleosomes (Clark et al., 1993). Such pioneer factors have been suggested to induce nucleosome repositioning, possibly by recruiting hyperdynamic histone variants, such as H2A.Z and H3.3 and other chromatin and DNA modifying proteins, to allow binding of *cis*-regulatory elements by transcriptional complexes (Chen and Dent, 2014; Spitz and Furlong, 2012; Zaret and Carroll, 2011). To assess whether foxd3 affects nucleosomal positioning on NC enhancers, we analyzed nucleosome profiles in *foxd3*-mutant and control neural crest cells. To this end, we generated nucleosomal occupancy tracks using the NucleoATAC algorithm that enables calling nucleosome positions using Tn5 footprints embedded in ATAC-Seq data (Schep et al., 2015). *k*-means clustering identified cohesive groups of elements that presented significant differences in nucleosomal patterns between *foxd3*-mutant (CC) and control (C) NC. Interestingly, while no changes in chromatin architecture at promoters were observed, nucleosomal clustering at 5–6ss singled out groups with differential nucleosomal density in *foxd3*-mutants (Figure S6F). Overall, we found that foxd3 influences the nucleosome positioning at NC regulatory elements in a context-dependent manner, resulting in both "permissive" and "repressive" chromatin organizations (Figure 7G). We find clear evidence of permissive foxd3 occupancy resulting either in the removal of the nucleosomes from the core enhancer region (early foxd3 binding; 75% epiboly to 1–2ss) or maintenance of nucleosome-free conformation (later foxd3 binding, from 5–6ss). Both processes were altered in *foxd3*-mutants, resulting in compaction of enhancer cores that are habitually nucleosome-free (Figure 7G′). Conversely, at other elements, repressive foxd3 binding was associated with the nucleosomal maintenance and compaction, as absence of functional foxd3 protein in mutant NC resulted in clearing of nucleosomes from enhancer cores (Figure 7G″).

To analyze genome-wide changes in nucleosomal positioning at gene regulatory regions upon *foxd3* gene perturbation, we performed *k*-means clustering and identified cohesive groups of elements showing differential nucleosomal patterns between *foxd3*-mutants and controls. At both stages of development analyzed (75% epiboly and 5–6ss), we identified clusters of nucleosome-loose elements with activating patterns and nucleosome-compact clusters with repressive patterns but displaying opposite nucleosomal positioning in *foxd3*-mutants (Figure 7H). Functional annotation of foxd3-bound regulatory elements belonging to the identified nucleosomal clusters at early stage suggests foxd3-directed activation of NC and stem cell development programs, as well as preparation for neural crest migration (Figure 7H′; epi-activate). Concurrently foxd3 appears to directly negatively control premature NC and stem cell differentiation, and formation of derivatives (peripheral nervous system, melanocytes) (Figure 7H′; epi-repress). Similarly, in the *bona fide* premigratory NC cells at 5–6ss, foxd3-mediated rearrangements of nucleosomes directly control activation of relevant signaling pathways (FGF, ERK1-ERK2 MAPK, as well as non-canonical Wnt signaling), as well as the onset of gliogenesis (Figure 7H′; 5–6ss-activate), while, at the same time, directly repressing late differentiation

events (pigment cells, axonogenesis) and components of signaling pathways no longer active in migrating crest (Id, Notch/Delta) (Figure 7H′; 5–6ss-repress).

DISCUSSION

Gene expression is the product of interplay between proximal and distal *cis*-regulatory elements, controlling competence at the chromatin level (Ong and Corces, 2012; Wang et al., 2015). Moreover, broad epigenetic changes to the *cis*-regulatory landscape, including histone and DNA demethylation, histone acetylation, and loss of heterochromatin characterize different stages of transition from naive to primed pluripotency (Krishnakumar and Blelloch, 2013). Several mechanisms explaining how Foxd3 promotes pluripotency *in vitro* have been proposed. FoxD3 can recruit Tle4 to repress differentiation-associated genes induced by NFAT signaling through regulation of histone de-acetylation (Zhu et al., 2014). In two recent studies investigating the transition from ESCs to EpiCs, EpiLCs, and PGCKs, mouse FoxD3 was implicated in the regulation of stem cell pluripotency by associating to different enhancer marks and subsequently manipulating transcriptional competency of downstream genes (Liber et al., 2010). The first report showed that FoxD3-bound enhancers associated with genes primed for expression upon exit from naive pluripotency, with FoD3 promoting nucleosome depletion by recruiting SWI/SNF complex chromatin remodeler Brg1, while simultaneously acting as a repressor and preventing enhancer acetylation by recruiting HDACs (Krishnakumar et al., 2016). The other study showed that FoxD3-bound active enhancers associated with highly expressed genes that become silenced upon exit from naive pluripotency, where corresponding enhancers were decommissioned through recruitment of Lsd1, and a reduction in p300 activity (Respuela et al., 2016). Surprisingly, the two studies found a minimal overlap (only ~12%) in FoxD3 bound peaks (Plank et al., 2014; Sweet, 2016; Yong et al., 2016). The discrepancies between the different putative mechanisms of FoxD3 re-enforced the need for *in vivo* studies that would characterize the regulatory context within which FoxD3 mediates different activator and repressor roles across developmental time.

Foxd3 Is a Pioneering Factor for NC Specification

The studies described above suggest FoxD3 plays an array of complex independent roles during NC ontogeny, but its role during NC specification has remained elusive. Although Foxd3 was thought to act mostly as a transcriptional repressor, previous reports failed to recover more differentially upregulated versus downregulated genes in *foxd3* mutant cells (Respuela et al., 2016; Yaklichkin et al., 2007). Strikingly, our analysis showed that foxd3 plays a central activating role in NC specification, both directly and indirectly controlling the expression of an entire NC specification module. We present evidence that foxd3 acts at a global level to prime NC factors by modulating the accessibility of their *cis*-regulatory elements. Thus, much like its relatives, FoxA1 and FoxA2, shown to regulate enhancer dynamics for specific gene expression controlling pluripotent stem cell potential, cell fate transitions, lineage choice, and differentiation (Adam et al., 2015; Sérandour et al., 2011; Zaret and Carroll, 2011), foxd3 acts as a pioneer factor in the NC. By studying dynamics of chromatin opening across several stages, we identified a set of hotspot enhancers, a substantial portion of whose accessibility was dependent on a direct foxd3 binding. Quantification of accessibility levels using normalized ATAC assay and statistical differential binding analysis indicated that defects in *foxd3*-mutant cells are most striking at the onset of enhancer opening and affect early genes at the onset of NC specification, late genes at the onset of migration and genes involved in the multipotent progenitor potential maintenance.

Foxd3 Affects H3K27 Acetylation on NC Enhancers

Previous studies suggested that one of the *modi operandi* of pioneer factors was the recruitment of H3K27 acetyltransferase activity, a hallmark histone modification of active enhancers (Choi et al., 2016; Kerschner et al., 2014). In contrast, a recent report found that, following FoxA1/A2 activity, accessible nucleosomes in liver-specific enhancers had reduced H3K27Ac, suggesting that the initial role of pioneer factors in opening and controlling nucleosome occupancy at enhancers was temporally uncoupled from the acetylation role (Iwafuchi-Doi et al., 2016). We found that lack of foxd3 during NC specification resulted in differential K27 acetylation, with some NC regulatory elements showing depletion and others an increase in H3K27Ac mark in mutant embryos. We show that early NC specifiers, downregulated in *foxd3*-mutants, are controlled positively via this mechanism, as they associated to the K27Ac-depleted elements with a high statistical significance. At the same time, we demonstrate that those *cis*-regulatory elements, which show significant areas of hyperacetylation in mutants, negatively control essential components of Wnt signaling pathway. Therefore, in NC cells foxd3 activity both enables and inhibits H3K27 Acetylation of NC regulatory elements, thus promoting both the activation of NC specification genes and the repression of factors that need to be downregulated for the NC migration/differentiation to proceed.

Bimodal Action of foxd3

Here, we present strong evidence that during NC formation *in vivo*, in addition to its conventional role as a repressor (Yaklichkin et al., 2007), foxd3 acts as a pioneer factor to prime NC gene expression. In line with recent *in vitro* studies (Krishnakumar et al., 2016; Respuela et al., 2016), we demonstrate that foxd3 functions primarily by changing the chromatin landscape of *cis*-regulatory elements and sets up a number of hotspot NC gene enhancers (*k*-cluster 3.1), as well as later migratory NC regulatory elements required for the specification of distinct NC lineages. The foxd3 binding to the NC enhancers that were associated with the downregulated genes in the absence of foxd3 is strongly indicative of its direct central role in NC gene activation via enhancer priming during early steps of NC ontogeny. On the other hand, later in NC development, foxd3 represses or decommissions a considerable number of active enhancers associated with mesenchymal or neuronal genes found upregulated in *foxd3*-mutants. This indicates that, in the developing embryos, foxd3 is capable of modulating the NC chromatin regulatory landscape in a bimodal fashion, facilitating both permissive and repressive states. These mechanisms do not exhibit sharp temporal boundaries but instead occur

concomitantly, with a gradual shift toward the repressive activity after NC specification. Whether such bimodal activity of foxd3 could enable early NC fate transitions and maintenance of multipotency remains to be investigated in future.

Distinct Regulatory Co-factors Likely Underpin foxd3's Dual Mechanisms of Action

Regulation of gene expression is largely determined by co-operative interactions between different transcription factors that are dependent on underlying DNA binding motifs (Kato et al., 2004). For instance, another Fox pioneering factor, FoxA, was shown to both promote gene expression but also to co-occupy the enhancers of silenced genes such as *cdx2* together with transcriptional repressors such as Rfx and type II nuclear hormone receptor (Watts et al., 2011). Our combinatorial TF analyses uncovered a number of novel foxd3 co-factors required for either gene priming or repression that together control NC induction and maintain NC multipotency. One of the identified putative foxd3 co-binding partners required for the pioneering activity, zbtb3, was previously shown to be critical in the early embryonic development and stem cell self-renewal by promoting Nanog expression in mice (Ye et al., 2018). Interestingly, its fly homolog GAF was shown to influence chromatin organization, including promoting nucleosome removal by associating with chromatin remodeling complexes, such as nucleosome remodeling factor or facilitates chromatin transcription (FACT) (Adkins et al., 2006). Furthermore, ZFPs were revealed as most likely foxd3 partners during early NC development. Intriguingly, BRG1, a catalytic subunit of chromatin remodeling SWI/SNF complex previously shown to interact with foxd3 (Krishnakumar et al., 2016), is known to be attracted to targeted chromatin regions via its N-terminal ZFP-interaction domain (Kadam and Emerson, 2003). Thus, our results suggest foxd3/ZFP-dependent recruitment of Brg1 to the associated enhancers that subsequently leads to nucleosome depletion and enhancer activation. Other known NC factors, such as zic and tfap2, also seem to be playing a co-pioneering role together with foxd3 in early NC development as previously shown using foxd3/tfap2 double mutant analyses (Wang et al., 2011).

In search of co-repressing partners of foxd3, we identified a putative novel NC regulator, e2f6, that potentially co-represses NC differentiation genes together with foxd3. E2F6 exerts its repressive functionality through recruitment of PRC complexes in a DNA sequence-targeted fashion (Attwooll et al., 2005; Ogawa et al., 2002; Trimarchi et al., 2001). Notably, a previous study exploiting a similar strategy to ours to uncover TF motif co-occurrences on FoxA2 binding sites, which were associated with upregulated genes in FoxA1/2 mutants, also identified E2F6 as a potential co-repressor in mouse liver cells (Iwafuchi-Doi et al., 2016).

Here, we present striking evidence that, during NC ontogeny, foxd3 may switch from permissive to repressive nucleosome/chromatin organization of NC *cis*-regulatory elements to independently control NC specification and NC differentiation events. Furthermore, we identified potential distinctive transcription co-factors at different stages of NC ontogeny, indicating possible mechanisms underlying foxd3 bimodality. Thus, our current data provide a platform for future hypothesis-driven experiments that will be crucial for deciphering the exact mechanism of foxd3 bimodality underlying NC gene regulation *in vivo*.

The beta version of the interactive ShinyApp associated with the data produced in this study and Pagoda App (Fan et al., 2016) presenting single-cell catalogs can be downloaded from https://github.com/tsslab/foxd3. The live app can also be accessed here: https://livedataoxford.shinyapps.io/FoxD3-project-TSS-Lab/.

STAR★METHODS

Detailed methods are provided in the online version of this paper and include the following:

- KEY RESOURCES TABLE
- CONTACT FOR REAGENT AND RESOURCE SHARING
- EXPERIMENTAL MODEL AND SUBJECT DETAILS
  - Zebrafish Lines
- METHOD DETAILS
  - Cell Dissociation and FAC-Sorting
  - Bulk RNA Extraction, Library Preparation and Sequencing
  - Single Cell RNA Preparation Library Preparation and Sequencing
  - *In Situ* Hybridisation
  - Generation of Avi-Tagged *foxd3* Transgenic Line
  - Foxd3 Biotin-ChIP, Library Preparation and Sequencing
  - ATAC, Library Preparation and Sequencing
  - H3K27Ac ChIP, Library Preparation and Sequencing
  - Enhancer Reporter Constructs
  - Foxd3 Ectopic Expression Assay
  - Bioinformatic Processing
  - ATAC-Seq Processing
  - H3K27Ac-ChIP Processing
  - Foxd3 Biotin-ChIP Processing
  - Transcription Factor Binding Site Identification on ATAC-Seq Peaks
  - *K*-Means Clustering
  - Differential Chromatin Accessibility Analysis
- QUANTIFICATION AND STATISTICAL ANALYSIS
- DATA AND SOFTWARE AVAILABILITY

SUPPLEMENTAL INFORMATION

Supplemental Information includes six figures and can be found with this article online at https://doi.org/10.1016/j.devcel.2018.11.009.

ACKNOWLEDGMENTS

This work was supported by MRC (G0902418), Lister Institute prize, Leverhulme Trust grant (RPG-2015-026), and March of Dimes Basil O'Connor Award to T.S.-S.; SNF Fellowship to D.G.; and Radcliffe Department of Medicine Scholarship to M.L. We thank Ivan Candido-Ferreira for his help with the combinatorial transcription factor motif analysis and for sharing his custom R and Python scripts.

AUTHOR CONTRIBUTIONS

Conceptualization, T.S.-S., M.L., and D.G.; Methodology, R.M.W., M.L., and V.C.-M.; Software, D.G., M.L., and E.R.; Validation, T.S.-S., M.L., D.G., and R.M.W.; Formal Analysis, M.L., D.G., R.M.W., S.T., and T.S.-S.; Investigation, R.M.W., M.L., V.C.-M., U.S., and T.H.-H.; Writing – Original Draft, T.S.-S., M.L.,

and D.G.; Writing – Review & Editing, all authors; Visualization, T.S.-S., M.L., D.G., and E.R.; Supervision, T.S.-S.; Funding Acquisition, T.S.-S.

DECLARATION OF INTERESTS

The authors declare no competing interests.

Received: November 7, 2017
Revised: August 15, 2018
Accepted: October 31, 2018
Published: December 3, 2018

REFERENCES

Adam, R.C., Yang, H., Rockowitz, S., Larsen, S.B., Nikolova, M., Oristian, D.S., Polak, L., Kadaja, M., Asare, A., Zheng, D., et al. (2015). Pioneer factors govern super-enhancer dynamics in stem cell plasticity and lineage choice. Nature *521*, 366–370.

Adkins, N.L., Hagerman, T.A., and Georgel, P. (2006). Gaga protein: a multi-faceted transcription factor. Biochem. Cell Biol. *84*, 559–567.

Adli, M., and Bernstein, B.E. (2011). Whole-genome chromatin profiling from limited numbers of cells using nano-ChIP-seq. Nat. Protoc. *6*, 1656–1668.

Attwooll, C., Oddi, S., Cartwright, P., Prosperini, E., Agger, K., Steensgaard, P., Wagener, C., Sardet, C., Moroni, M.C., and Helin, K. (2005). A novel repressive E2F6 complex containing the polycomb group protein, EPC1, that interacts with EZH2 in a proliferation-specific manner. J. Biol. Chem. *280*, 1199–1208.

Baggiolini, A., Varum, S., Mateos, J.M., Bettosini, D., John, N., Bonalli, M., Ziegler, U., Dimou, L., Clevers, H., Furrer, R., et al. (2015). Premigratory and migratory neural crest cells are multipotent in vivo. Cell Stem Cell *16*, 314–322.

Bailey, T.L., Johnson, J., Grant, C.E., and Noble, W.S. (2015). The MEME Suite. Nucleic Acids Res. *43*, W39–W49.

Beby, F., and Lamonerie, T. (2013). The homeobox gene Otx2 in development and disease. Exp. Eye Res. *111*, 9–16.

Bery, A., Mérot, Y., and Rétaux, S. (2016). Genes expressed in mouse cortical progenitors are enriched in Pax, Lhx, and Sox transcription factor putative binding sites. Brain Res. *1633*, 37–51.

Betancur, P., Bronner-Fraser, M., and Sauka-Spengler, T. (2010). Assembling neural crest regulatory circuits into a gene regulatory network. Annu. Rev. Cell Dev. Biol. *26*, 581–603.

Briggs, J.A., Weinreb, C., Wagner, D.E., Megason, S., Peshkin, L., Kirschner, M.W., and Klein, A.M. (2018). The dynamics of gene expression in vertebrate embryogenesis at single-cell resolution. Science *360*, https://doi.org/10.1126/science.aar5780.

Bronner-Fraser, M., and Fraser, S.E. (1988). Cell lineage analysis reveals multipotency of some avian neural crest cells. Nature *335*, 161–164.

Buenrostro, J.D., Giresi, P.G., Zaba, L.C., Chang, H.Y., and Greenleaf, W.J. (2013). Transposition of native chromatin for fast and sensitive epigenomic profiling of open chromatin, DNA-binding proteins and nucleosome position. Nat. Methods *10*, 1213–1218.

Buitrago-Delgado, E., Nordin, K., Rao, A., Geary, L., and LaBonne, C. (2015). Neurodevelopment. Shared regulatory programs suggest retention of blastula-stage potential in neural crest cells. Science *348*, 1332–1335.

Carney, T.J., Dutton, K.A., Greenhill, E., Delfino-Machín, M., Dufourcq, P., Blader, P., and Kelsh, R.N. (2006). A direct role for Sox10 in specification of neural crest-derived sensory neurons. Development *133*, 4619–4630.

Chen, T., and Dent, S.Y. (2014). Chromatin modifiers and remodellers: regulators of cellular differentiation. Nat. Rev. Genet. *15*, 93–106.

Choi, S.H., Gearhart, M.D., Cui, Z., Bosnakovski, D., Kim, M., Schennum, N., and Kyba, M. (2016). DUX4 recruits p300/CBP through its C-terminus and induces global H3K27 acetylation changes. Nucleic Acids Res. *44*, 5161–5173.

Clark, K.L., Halay, E.D., Lai, E., and Burley, S.K. (1993). Co-crystal structure of the HNF-3/fork head DNA-recognition motif resembles histone H5. Nature *364*, 412–420.

Dee, C.T., Hirst, C.S., Shih, Y.H., Tripathi, V.B., Patient, R.K., and Scotting, P.J. (2008). Sox3 regulates both neural fate and differentiation in the zebrafish ectoderm. Dev. Biol. *320*, 289–301.

Dobin, A., Davis, C.A., Schlesinger, F., Drenkow, J., Zaleski, C., Jha, S., Batut, P., Chaisson, M., and Gingeras, T.R. (2013). STAR: ultrafast universal RNA-seq aligner. Bioinformatics *29*, 15–21.

Dottori, M., Gross, M.K., Labosky, P., and Goulding, M. (2001). The winged-helix transcription factor Foxd3 suppresses interneuron differentiation and promotes neural crest cell fate. Development *128*, 4127–4138.

Fan, J., Salathia, N., Liu, R., Kaeser, G.E., Yung, Y.C., Herman, J.L., Kaper, F., Fan, J.B., Zhang, K., Chun, J., et al. (2016). Characterizing transcriptional heterogeneity through pathway and gene set overdispersion analysis. Nat. Methods *13*, 241–244.

Gaubatz, S., Wood, J.G., and Livingston, D.M. (1998). Unusual proliferation arrest and transcriptional control properties of a newly discovered E2F family member, E2F-6. Proc. Natl. Acad. Sci. USA *95*, 9190–9195.

Gilmour, D.T., Maischein, H.M., and Nüsslein-Volhard, C. (2002). Migration and function of a glial subtype in the vertebrate peripheral nervous system. Neuron *34*, 577–588.

Harris, M.L., and Erickson, C.A. (2007). Lineage specification in neural crest cell pathfinding. Dev. Dyn. *236*, 1–19.

Heinz, S., Benner, C., Spann, N., Bertolino, E., Lin, Y.C., Laslo, P., Cheng, J.X., Murre, C., Singh, H., and Glass, C.K. (2010). Simple combinations of lineage-determining transcription factors prime cis-regulatory elements required for macrophage and B cell identities. Mol. Cell *38*, 576–589.

Hochgreb-Hägele, T., and Bronner, M.E. (2013). A novel FoxD3 gene trap line reveals neural crest precursor movement and a role for FoxD3 in their specification. Dev. Biol. *374*, 1–11.

Hromas, R., Ye, H., Spinella, M., Dmitrovsky, E., Xu, D., and Costa, R.H. (1999). Genesis, a Winged Helix transcriptional repressor, has embryonic expression limited to the neural crest, and stimulates proliferation in vitro in a neural development model. Cell Tissue Res. *297*, 371–382.

Iwafuchi-Doi, M., Donahue, G., Kakumanu, A., Watts, J.A., Mahony, S., Pugh, B.F., Lee, D., Kaestner, K.H., and Zaret, K.S. (2016). The Pioneer transcription factor FoxA maintains an accessible nucleosome configuration at enhancers for tissue-specific gene activation. Mol. Cell *62*, 79–91.

Joshi, N.A., and Fass, J.N. (2011). Sickle: A Sliding-Window, Adaptive, Quality-Based Trimming Tool for FastQ files, Version 1.33 [Software], Available online at: https://github.com/najoshi/sickle.

Kadam, S., and Emerson, B.M. (2003). Transcriptional specificity of human SWI/SNF BRG1 and BRM chromatin remodeling complexes. Mol. Cell *11*, 377–389.

Kato, M., Hata, N., Banerjee, N., Futcher, B., and Zhang, M.Q. (2004). Identifying combinatorial regulation of transcription factors and binding motifs. Genome Biol. *5*, R56.

Kelsh, R.N., Dutton, K., Medlin, J., and Eisen, J.S. (2000). Expression of zebrafish fkd6 in neural crest-derived glia. Mech. Dev. *93*, 161–164.

Kerschner, J.L., Gosalia, N., Leir, S.H., and Harris, A. (2014). Chromatin remodeling mediated by the FOXA1/A2 transcription factors activates CFTR expression in intestinal epithelial cells. Epigenetics *9*, 557–565.

Kim, J.H., Lee, S.R., Li, L.H., Park, H.J., Park, J.H., Lee, K.Y., Kim, M.K., Shin, B.A., and Choi, S.Y. (2011). High cleavage efficiency of a 2A peptide derived from porcine teschovirus-1 in human cell lines, zebrafish and mice. PLoS One *6*, e18556.

Kimmel, C.B., Ballard, W.W., Kimmel, S.R., Ullmann, B., and Schilling, T.F. (1995). Stages of embryonic development of the zebrafish. Dev. Dyn. *203*, 253–310.

Kos, R., Reedy, M.V., Johnson, R.L., and Erickson, C.A. (2001). The winged-helix transcription factor FoxD3 is important for establishing the neural crest lineage and repressing melanogenesis in avian embryos. Development *128*, 1467–1479.

Krishnakumar, R., and Blelloch, R.H. (2013). Epigenetics of cellular reprogramming. Curr. Opin. Genet. Dev. *23*, 548–555.

Krishnakumar, R., Chen, A.F., Pantovich, M.G., Danial, M., Parchem, R.J., Labosky, P.A., and Blelloch, R. (2016). FOXD3 regulates pluripotent stem cell potential by simultaneously initiating and repressing enhancer activity. Cell Stem Cell *18*, 104–117.

Krispin, S., Nitzan, E., Kassem, Y., and Kalcheim, C. (2010). Evidence for a dynamic spatiotemporal fate map and early fate restrictions of premigratory avian neural crest. Development *137*, 585–595.

Kubic, J.D., Lui, J.W., Little, E.C., Ludvik, A.E., Konda, S., Salgia, R., Aplin, A.E., and Lang, D. (2015). PAX3 and FOXD3 promote CXCR4 expression in melanoma. J. Biol. Chem. *290*, 21901–21914.

Langmead, B., Trapnell, C., Pop, M., and Salzberg, S.L. (2009). Ultrafast and memory-efficient alignment of short DNA sequences to the human genome. Genome Biol. *10*, R25.

Lee, M.T., Bonneau, A.R., Takacs, C.M., Bazzini, A.A., DiVito, K.R., Fleming, E.S., and Giraldez, A.J. (2013). Nanog, Pou5f1 and SoxB1 activate zygotic gene expression during the maternal-to-zygotic transition. Nature *503*, 360–364.

Leseva, M., Santostefano, K.E., Rosenbluth, A.L., Hamazaki, T., and Terada, N. (2013). E2f6-mediated repression of the meiotic Stag3 and Smc1β genes during early embryonic development requires Ezh2 and not the de novo methyltransferase Dnmt3b. Epigenetics *8*, 873–884.

Li, W., and Cornell, R.A. (2007). Redundant activities of Tfap2a and Tfap2c are required for neural crest induction and development of other non-neural ectoderm derivatives in zebrafish embryos. Dev. Biol. *304*, 338–354.

Liao, Y., Smyth, G.K., and Shi, W. (2014). featureCounts: an efficient general purpose program for assigning sequence reads to genomic features. Bioinformatics *30*, 923–930.

Liber, D., Domaschenz, R., Holmqvist, P.H., Mazzarella, L., Georgiou, A., Leleu, M., Fisher, A.G., Labosky, P.A., and Dillon, N. (2010). Epigenetic priming of a pre-B cell-specific enhancer through binding of Sox2 and Foxd3 at the ESC stage. Cell Stem Cell *7*, 114–126.

Lister, J.A., Cooper, C., Nguyen, K., Modrell, M., Grant, K., and Raible, D.W. (2006). Zebrafish Foxd3 is required for development of a subset of neural crest derivatives. Dev. Biol. *290*, 92–104.

Liu, Y., and Labosky, P.A. (2008). Regulation of embryonic stem cell self-renewal and pluripotency by Foxd3. Stem Cells *26*, 2475–2484.

Love, M.I., Huber, W., and Anders, S. (2014). Moderated estimation of fold change and dispersion for RNA-seq data with DESeq2. Genome Biol. *15*, 550.

Manderfield, L.J., Engleka, K.A., Aghajanian, H., Gupta, M., Yang, S., Li, L., Baggs, J.E., Hogenesch, J.B., Olson, E.N., and Epstein, J.A. (2014). Pax3 and hippo signaling coordinate melanocyte gene expression in neural crest. Cell Rep. *9*, 1885–1895.

Marletaz, F., Maeso, I., Faas, L., Isaacs, H.V., and Holland, P.W. (2015). Cdx ParaHox genes acquired distinct developmental roles after gene duplication in vertebrate evolution. BMC Biol. *13*, 56.

McLean, C.Y., Bristor, D., Hiller, M., Clarke, S.L., Schaar, B.T., Lowe, C.B., Wenger, A.M., and Bejerano, G. (2010). GREAT improves functional interpretation of cis-regulatory regions. Nat. Biotechnol. *28*, 495–501.

Montero-Balaguer, M., Lang, M.R., Sachdev, S.W., Knappmeyer, C., Stewart, R.A., De La Guardia, A., Hatzopoulos, A.K., and Knapik, E.W. (2006). The mother superior mutation ablates foxd3 activity in neural crest progenitor cells and depletes neural crest derivatives in zebrafish. Dev. Dyn. *235*, 3199–3212.

Mundell, N.A., and Labosky, P.A. (2011). Neural crest stem cell multipotency requires Foxd3 to maintain neural potential and repress mesenchymal fates. Development *138*, 641–652.

Murdoch, B., DelConte, C., and García-Castro, M.I. (2012). Pax7 lineage contributions to the mammalian neural crest. PLoS One *7*, e41089.

Nitzan, E., Krispin, S., Pfaltzgraff, E.R., Klar, A., Labosky, P.A., and Kalcheim, C. (2013). A dynamic code of dorsal neural tube genes regulates the segregation between neurogenic and melanogenic neural crest cells. Development *140*, 2269–2279.

Odenthal, J., and Nüsslein-Volhard, C. (1998). fork head domain genes in zebrafish. Dev. Genes Evol. *208*, 245–258.

Ogawa, H., Ishiguro, K., Gaubatz, S., Livingston, D.M., and Nakatani, Y. (2002). A complex with chromatin modifiers that occupies E2F- and Myc-responsive genes in G0 cells. Science *296*, 1132–1136.

Ong, C.T., and Corces, V.G. (2012). Enhancers: emerging roles in cell fate specification. EMBO Rep. *13*, 423–430.

Orlando, D.A., Chen, M.W., Brown, V.E., Solanki, S., Choi, Y.J., Olson, E.R., Fritz, C.C., Bradner, J.E., and Guenther, M.G. (2014). Quantitative ChIP-seq normalization reveals global modulation of the epigenome. Cell Rep. *9*, 1163–1170.

Picelli, S., Faridani, O.R., Björklund, A.K., Winberg, G., Sagasser, S., and Sandberg, R. (2014). Full-length RNA-seq from single cells using Smart-seq2. Nat. Protoc. *9*, 171–181.

Plank, J.L., Suflita, M.T., Galindo, C.L., and Labosky, P.A. (2014). Transcriptional targets of Foxd3 in murine ES cells. Stem Cell Res. *12*, 233–240.

Pohl, B.S., and Knöchel, W. (2001). Overexpression of the transcriptional repressor FoxD3 prevents neural crest formation in Xenopus embryos. Mech. Dev. *103*, 93–106.

Powell, D.R., Hernandez-Lagunas, L., LaMonica, K., and Artinger, K.B. (2013). Prdm1a directly activates foxd3 and tfap2a during zebrafish neural crest specification. Development *140*, 3445–3455.

Rada-Iglesias, A., Bajpai, R., Prescott, S., Brugmann, S.A., Swigut, T., and Wysocka, J. (2012). Epigenomic annotation of enhancers predicts transcriptional regulators of human neural crest. Cell Stem Cell *11*, 633–648.

Ramírez, F., Ryan, D.P., Grüning, B., Bhardwaj, V., Kilpert, F., Richter, A.S., Heyne, S., Dündar, F., and Manke, T. (2016). deepTools2: a next generation web server for deep-sequencing data analysis. Nucleic Acids Res. *44*, W160–W165.

Respuela, P., Nikolić, M., Tan, M., Frommolt, P., Zhao, Y., Wysocka, J., and Rada-Iglesias, A. (2016). Foxd3 promotes exit from naive pluripotency through enhancer decommissioning and inhibits germline specification. Cell Stem Cell *18*, 118–133.

Robinson, M.D., McCarthy, D.J., and Smyth, G.K. (2010). edgeR: a Bioconductor package for differential expression analysis of digital gene expression data. Bioinformatics *26*, 139–140.

Satija, R., Farrell, J.A., Gennert, D., Schier, A.F., and Regev, A. (2015). Spatial reconstruction of single-cell gene expression data. Nat. Biotechnol. *33*, 495–502.

Sauka-Spengler, T., and Bronner-Fraser, M. (2008). A gene regulatory network orchestrates neural crest formation. Nat. Rev. Mol. Cell Biol. *9*, 557–568.

Schep, A.N., Buenrostro, J.D., Denny, S.K., Schwartz, K., Sherlock, G., and Greenleaf, W.J. (2015). Structured nucleosome fingerprints enable high-resolution mapping of chromatin architecture within regulatory regions. Genome Res. *25*, 1757–1770.

Sérandour, A.A., Avner, S., Percevault, F., Demay, F., Bizot, M., Lucchetti-Miganeh, C., Barloy-Hubler, F., Brown, M., Lupien, M., Métivier, R., et al. (2011). Epigenetic switch involved in activation of pioneer factor FOXA1-dependent enhancers. Genome Res. *21*, 555–565.

Simões-Costa, M., and Bronner, M.E. (2015). Establishing neural crest identity: a gene regulatory recipe. Development *142*, 242–257.

Soufi, A., Garcia, M.F., Jaroszewicz, A., Osman, N., Pellegrini, M., and Zaret, K.S. (2015). Pioneer transcription factors target partial DNA motifs on nucleosomes to initiate reprogramming. Cell *161*, 555–568.

Spitz, F., and Furlong, E.E. (2012). Transcription factors: from enhancer binding to developmental control. Nat. Rev. Genet. *13*, 613–626.

Stark, R., and Brown, G. (2011). Diffbind: Differential Binding Analysis of ChIP-Seq Peak Data (Bioconductor).

Stewart, R.A., Arduini, B.L., Berghmans, S., George, R.E., Kanki, J.P., Henion, P.D., and Look, A.T. (2006). Zebrafish foxd3 is selectively required for neural crest specification, migration and survival. Dev. Biol. *292*, 174–188.

Sweet, D.J. (2016). Foxd3: A repressor, an activator, or both? Cell Stem Cell *18*, 1–2.

Teng, L., Mundell, N.A., Frist, A.Y., Wang, Q., and Labosky, P.A. (2008). Requirement for Foxd3 in the maintenance of neural crest progenitors. Development *135*, 1615–1624.

Trimarchi, J.M., Fairchild, B., Wen, J., and Lees, J.A. (2001). The E2F6 transcription factor is a component of the mammalian Bmi1-containing polycomb complex. Proc. Natl. Acad. Sci. USA *98*, 1519–1524.

Trinh, L.A., Chong-Morrison, V., Gavriouchkina, D., Hochgreb-Hägele, T., Senanayake, U., Fraser, S.E., and Sauka-Spengler, T. (2017). Biotagging of specific cell populations in zebrafish reveals gene regulatory logic encoded in the nuclear transcriptome. Cell Rep. *19*, 425–440.

van Heeringen, S.J., and Veenstra, G.J. (2011). GimmeMotifs: a de novo motif prediction pipeline for ChIP-sequencing experiments. Bioinformatics *27*, 270–271.

Wang, P., Qin, J., Qin, Y., Zhu, Y., Wang, L.Y., Li, M.J., Zhang, M.Q., and Wang, J. (2015). ChIP-Array 2: Integrating multiple omics data to construct gene regulatory networks. Nucleic Acids Res. *43*, W264–W269.

Wang, W.D., Melville, D.B., Montero-Balaguer, M., Hatzopoulos, A.K., and Knapik, E.W. (2011). Tfap2a and Foxd3 regulate early steps in the development of the neural crest progenitor population. Dev. Biol. *360*, 173–185.

Watts, J.A., Zhang, C., Klein-Szanto, A.J., Kormish, J.D., Fu, J., Zhang, M.Q., and Zaret, K.S. (2011). Study of FoxA pioneer factor at silent genes reveals Rfx-repressed enhancer at Cdx2 and a potential indicator of esophageal adenocarcinoma development. PLoS Genet. *7*, e1002277.

Weirauch, M.T., Yang, A., Albu, M., Cote, A.G., Montenegro-Montero, A., Drewe, P., Najafabadi, H.S., Lambert, S.A., Mann, I., Cook, K., et al. (2014). Determination and inference of eukaryotic transcription factor sequence specificity. Cell *158*, 1431–1443.

Westerfield, M. (2000). The Zebrafish Book. A Guide for the Laboratory Use of Zebrafish (Danio rerio), Fourth Edition (University of Oregon Press).

Xu, J., Pope, S.D., Jazirehi, A.R., Attema, J.L., Papathanasiou, P., Watts, J.A., Zaret, K.S., Weissman, I.L., and Smale, S.T. (2007). Pioneer factor interactions and unmethylated CpG dinucleotides mark silent tissue-specific enhancers in embryonic stem cells. Proc. Natl. Acad. Sci. USA *104*, 12377–12382.

Xu, J., Watts, J.A., Pope, S.D., Gadue, P., Kamps, M., Plath, K., Zaret, K.S., and Smale, S.T. (2009). Transcriptional competence and the active marking of tissue-specific enhancers by defined transcription factors in embryonic and induced pluripotent stem cells. Genes Dev. *23*, 2824–2838.

Yaklichkin, S., Steiner, A.B., Lu, Q., and Kessler, D.S. (2007). FoxD3 and Grg4 physically interact to repress transcription and induce mesoderm in Xenopus. J. Biol. Chem. *282*, 2548–2557.

Ye, B., Liu, B., Yang, L., Zhu, X., Zhang, D., Wu, W., Zhu, P., Wang, Y., Wang, S., Xia, P., et al. (2018). LncKdm2b controls self-renewal of embryonic stem cells via activating expression of transcription factor Zbtb3. EMBO J. *37*, https://doi.org/10.15252/embj.201797174.

Ye, T., Krebs, A.R., Choukrallah, M.A., Keime, C., Plewniak, F., Davidson, I., and Tora, L. (2011). seqMINER: an integrated ChIP-seq data interpretation platform. Nucleic Acids Res. *39*, e35.

Yong, J.S., Intriago-Baldeón, D.P., and Lam, E.W. (2016). FOXD3 controls pluripotency through modulating enhancer activity. Stem Cell. Investig. *3*, 17.

Zaret, K.S., and Carroll, J.S. (2011). Pioneer transcription factors: establishing competence for gene expression. Genes Dev. *25*, 2227–2241.

Zhang, Y., Liu, T., Meyer, C.A., Eeckhoute, J., Johnson, D.S., Bernstein, B.E., Nusbaum, C., Myers, R.M., Brown, M., Li, W., et al. (2008). Model-based analysis of ChIP-Seq (MACS). Genome Biol. *9*, R137.

Zhu, L., Zhang, S., and Jin, Y. (2014). Foxd3 suppresses NFAT-mediated differentiation to maintain self-renewal of embryonic stem cells. EMBO Rep. *15*, 1286–1296.

STAR★METHODS

KEY RESOURCES TABLE

REAGENT or RESOURCE	SOURCE	IDENTIFIER
Antibodies		
H3K27ac antibody	Abcam	Cat#Ab4729; RRID: AB_2118291
IgG antibody	Millipore	Cat#12-370; RRID: AB_145841
Critical Commercial Assays		
RNAqueous Micro Total RNA isolation kit	Ambion	Cat#AM1931
SmartSeq2 V4 kit	Takara Clontech	Cat#634889
Nextera XT library preparation kit	Illumina	Cat#FC-131-1024
Dynabeads Protein A	Life Technologies	Cat#10006D
Dynabeads Streptavidin M-280	Invitrogen	Cat#11206D
MicropPlex Library Preparation kit	Diagenode	Cat#05010012
NextSeq ® 500/550 High Output Kit v2 (75 cycles)	Illumina	Cat#FC-404-2005
Nextera DNA kit	Illumina	Cat#FC-121-1030
NEB Next High-Fidelity 2X PCR Mas-ter Mix	New England Biolabs	Cat#M0543S
Long Range HotStart PCR kit	KAPA Biosystems	Cat#KK3501
InFusion HD Cloning kit	Clontech	Cat#638910
DIG RNA Labelling Kit	Roche	Cat#11277073910
Deposited Data		
RNA-seq data (inc. single cell)	This paper	ShinyApp GEO: GSE106676, Pagoda https://github.com/tsslab/foxd3
ChIP-seq data	This paper	ShinyApp GEO: GSE106676
ATAC-seq data	This paper	ShinyApp GEO: GSE106676
Experimental Models: Organisms/Strains		
Zebrafish Gt(foxd3-citrine)^{ct110}	Sauka-Spengler laboratory	ct110
Zebrafish Gt(foxd3-mCherry)^{ct110R}	Sauka-Spengler laboratory	ct110R
TgBAC(foxd3-Avi-2A-Citrine)^{ox161}	Sauka-Spengler laboratory	ox161
Tg(ubiq:NLS-BirA-2A-Cherry)^{ox114}	Sauka-Spengler laboratory	ox114
Recombinant DNA		
BAC clone CH211-196F13	CHORI https://bacpacresources.org	CH211-196F13
Software and Algorithms		
Sickle	Joshi and Fass (2011)	https://github.com/najoshi/sickle
STAR 2.4.2a	Dobin et al. (2013)	https://github.com/alexdobin/STAR
FeatureCounts (v1.4.6-p4)	Liao et al. (2014)	http://bioinf.wehi.edu.au/featureCounts
R v3.4.2	R Core Team	https://www.r-project.org/
DESeq2 (v.1.14.1)	Love et al. (2014)	https://bioconductor.org/packages/release/bioc/html/DESeq2.html
edgeR	Robinson et al. (2010)	https://bioconductor.org/biocLite.R
SCDE	Fan et al. (2016)	http://hms-dbmi.github.io/scde/index.html
PAGODA	Fan et al. (2016)	http://hms-dbmi.github.io/scde/index.html
Bowtie (v.1.0.0)	Langmead et al. (2009)	http://bowtie-bio.sourceforge.net/bowtie2/index.html
Bedtools (v.2.15.0)	Langmead et al. (2009)	https://github.com/arq5x/bedtools
MACS2 (v2.1.0)	Zhang et al. (2008)	https://github.com/taoliu/MACS
HOMER (v.4.4)	Heinz et al. (2010)	http://homer.ucsd.edu/homer/index.html
SeqMINER	Ye et al. (2011)	http://seqminer.genomic.codes

(*Continued on next page*)

Continued		
REAGENT or RESOURCE	SOURCE	IDENTIFIER
Deeptools (v.2.2.2)	Ramírez et al. (2016)	https://github.com/deeptools/deepTools
MEME suite	Bailey et al. (2015)	http://meme-suite.org/doc/download.html
Gimmemotifs (v.0.9.0.3)	van Heeringen and Veenstra (2011)	https://gimmemotifs.readthedocs.io/en/master/
GREAT	McLean et al. (2010)	http://great.stanford.edu/public/html/
DiffBind	Stark and Brown (2011)	https://bioconductor.org/packages/release/bioc/html/DiffBind.html

CONTACT FOR REAGENT AND RESOURCE SHARING

Further information and requests for resources and reagents should be directed to and will be fulfilled by the Lead Contact, Tatjana Sauka-Spengler (tatjana.sauka-spengler@imm.ox.ac.uk).

EXPERIMENTAL MODEL AND SUBJECT DETAILS

For this study, both females and males of transgenic and wild-type zebrafish strains were used. Animals that were bred were from 3 months old to 2 years old. Zebrafish embryos that were used for the experiments were between 8-16 hours post fertilisation.

Zebrafish Lines

Genetrap line, *Gt(foxd3-citrine)*^{ct110} was generated by (Hochgreb-Hägele and Bronner, 2013). Animals were handled in accordance to procedures authorized by the UK Home Office in accordance with UK law (Animals [Scientific Procedures] Act 1986) and the recommendations in the Guide for the Care and Use of Laboratory Animals. All vertebrate animal work was performed at the facilities of Oxford University Biomedical Services. Adult fish were maintained as described previously (Westerfield, 2000). In brief, adult fish were exposed to 12 hour light – 12 hour dark cycle (8am to 10pm light; 10pm to 8am dark), kept in a closed recirculating system water at 27-28.5°C, fed 3-4 times a day, kept at 5 fish per 1L density. Embryos were staged as described previously (Kimmel et al., 1995). In brief, embryos were staged using a dissecting stereo-microscope. 75% epiboly stage was identified by observing a distinctively thicker dorsal side and visible epiblast, hypoblast and evacuation zone. 1-2ss – by observing first/second segment furrow. 5-6ss – counting 5/6 somites, apparent optical and Kupffer's vesicles and prominent polster. 14-16ss – counting 14/16 somites, observing otic placode, v-shaped trunk somites.

METHOD DETAILS

Cell Dissociation and FAC-Sorting

Selected embryos were dissociated with collagenase (20mg/ml in 0.05% trypsin) at 30°C for 10-15mins with intermittent pipetting to achieve a single cell suspension. Cells were centrifuged at 500g for 10mins and re-suspended in Hanks buffer, passed through a 0.22μm filter and centrifuged at 750g for 10min, pelleted cells were re-suspended in ~500μl Hanks buffer. Fluorescent positive cells were sorted and collected using BD FACS-Aria Fusion.

Bulk RNA Extraction, Library Preparation and Sequencing

FACS sorted cells were washed with PBS and stored at 80°C in lysis buffer. RNA was extracted using Ambion RNAqueous Micro Total RNA isolation kit (AM1931), checked on Bioanalyser, samples with RIN>7 were used to prepare cDNA using Takara Clontech SmartSeq2 V4 kit (634889). Sequencing libraries were prepared using Illumina Nextera XT library preparation kit (FC-131-1024). 75% Epiboly-stage cell libraries (Citrine-expressing, Citrine-Cherry-expressing and cells not expressing FoxD3) were sequenced using 80 bp reads using Illumina Nextseq500 platform. 5-6ss and 8ss cell libraries expressing FoxD3 (Citrine-expressing, Citrine-Cherry-expressing) were sequenced using 50bp paired-end (PE) reads on Illumina Hiseq2000 platform, and cells not expressing FoxD3 using 80bp PE reads on Illumina Nextseq500 platform. 12ss cell libraries expressing FoxD3 (Citrine-expressing, Citrine-Cherry-expressing) were sequenced using 100bp PE reads on Illumina Hiseq2000 platform. 14ss cell libraries (Citrine-expressing, Citrine-Cherry-expressing and cells not expressing FoxD3) were sequenced using 80bp PE reads using Illumina NextSeq500 platform.

Single Cell RNA Preparation Library Preparation and Sequencing

Individual cells were collected by FACS, cDNA was generated and sequencing libraries were prepared as previously described (Picelli et al., 2014). Briefly, mRNAs were primed with oligo-dT and reverse transcribed using an LNA-containing template switching oligo. Libraries were generated from amplified cDNA by Tagmentation with Tn5. Libraries were sequenced using 50 bp single end reads for 96 cells. A 4 x10^7 dilution of ERCC spike-in control was used.

In Situ Hybridisation

In situ hybridisation was performed according to standard protocols, as described previously (Hochgreb-Hägele and Bronner, 2013). Probe synthesis was conducted with the components of the DIG RNA Labelling Kit (Roche). In brief, the digoxigenin RNA probes were of an average length of 100-200 nucleotides. Embryos were fixed 24 hours in 4% paraformaldehyde 1x PBS, manually dechorionated and dehydrated overnight in methanol at -20°C. Then the embryos were rehydrated back to 100% PBT (1x PBS, 0.1% Tween 20). Embryos were treated 10 minutes with proteinase K (10 mg/ml in PBT). The reaction was stopped by rinsing in glycine (2 mg/ml in PBT). Embryos were postfixed in 4% paraformaldehyde in 1x PBS for 20 minutes and then rinsed in PBT. The embryos were prehybridized at least 1 hour at 70°C in hybridization buffer . The hybridization was done in the same buffer containing 50 ng to 100 ng of probe overnight at 70°C. Embryos were washed and were incubated overnight at 4°C with the preabsorbed alkalinephosphatase-coupled anti-digoxigenin antiserum at a 1/5000 dilution in a PBT buffer containing 2 mg/ml BSA, 2% sheep serum. Embryos were washed 6 times for 15 minutes each in PBT at room temperature. Detection was performed in alkaline phosphatase reaction buffer, the reaction was stopped in 1x PBS.

Generation of Avi-Tagged *foxd3* Transgenic Line

Tol2-mediated BAC transgenesis, as described in (Trinh et al., 2017), was used to generate *TgBAC(foxd3- Avi-2A-Citrine)*^{ox161} transgenic line. pGEM Avi-2A-Citrine-SV40pA-FRT-Kan-FRT recombination donor construct was generated by amplifying Avi-2A-Citrine cassette using Pfu polymerase (Pfu UltraII Hoststart PCR Master Mix, Agilent Technologies) and cloning it into the donor plasmid (#89890, Addgene) using InFusion (InFusion HD Cloning kit, Clontech). The full donor cassette contains a FLAG epitope, a TEV protease recognition sequence, an in-frame 48bp Avi-Tag, a *Citrine* reporter followed by a polyA tail and a Kanamycin selection cassette flanked by flippase recognition target (FRT) sequences. *Citrine* reporter is separated from the *foxd3*-Avi gene by a viral linker, 2A, sequence that mediates ribosome skipping, thus allowing for co-expression of both components from a single transcript (Kim et al., 2011). Genomic context of the *Danio rerio* BAC clone CH211-196F13 (203kb) was used for recombineering, as it harbours the full single exon ORF of the *foxd3* gene and the upstream regions (>200kb), thus encompassing not only the *foxd3* promoter but also associated *cis*-regulatory elements. The *foxd3* gene within the BAC was fused to the Avi-tag producing C-terminally Avi-tagged foxd3 (foxd3-Avi-2A-Citrine) expressed in endogenous-like fashion. NLS-BirA zebrafish transgenic line *Tg(ubiq:NLS-BirA-2A-Cherry)*^{ox114} expresses 3xHA epitope, nuclear localisation signal (NLS) sequence fused to BirA, viral 2A sequence and a *Citrine* reporter gene under the control of ubiquitous *ubb* promoter.

Foxd3 Biotin-ChIP, Library Preparation and Sequencing

Foxd3 Biotin-ChIP was performed on 700 for 75% epiboly, 350 for 1-2ss, 320 5-6ss experimental and BirA-only, 390 for 14ss whole embryos (~128,000 cells of interest) were used for a corresponding stage foxd3 Biotin-ChIP. Embryos were manually dechorionated, cells were dissociated with 20 strokes using pestle A in isotonic nuclei extraction buffer (NEB: 0.5% NP40, 0.25% Triton X, 10 mM Tris-HCl (pH 7.5), 3 mM $CaCl_2$, 0.25 M sucrose, 1mM DTT, 0.2 mM PMSF, 1X Proteinase inhibitor (PI) in a glass homogeniser and cross-linked using 1% formaldehyde at room temperature for 10 min. Fixation was quenched with 125 mM of glycine for 5min, cross-linker was washed-out by 3x pellet washes with 1x PBS (with 1X PI, 1 mM DTT and 0.2 mM PMSF) centrifuging at 2000g for 4min at 4°C. Pellets were re-suspended in NEB. Cell nuclei were expulsed with 20 strokes using pestle B in a glass homogeniser, pelleted and washed with 1 xPBS (with 1X PI, 1 mM DTT and 0.2 mM PMSF). Nuclei were lysed in SDS lysis buffer (0.7% SDS, 10mM EDTA, 50 mM Tris-HCl (pH 7.5), 1x PI). Cross-linked chromatin was sonicated at 12A, 10x (10s ON, 30s OFF) followed by 8A, 4x (30s ON, 30s OFF). Sheared chromatin samples were pre-cleared in pre-blocked Protein G beads (Dynabeads Protein G, Life Technologies) for 1 hour at 4°C. 1/20 of biotinChIP was collected as an input fraction and stored at -80°C. Pre-cleared chromatin samples were incubated on pre-blocked streptavidin beads (Dynabeads M-280 streptavidin beads, Invitrogen) o/n at 4°C. Beads were washed with SDS Wash Buffer (2% SDS, 10mM Tris-HCl (pH 7.5), 1 mM EDTA) at room temperature, followed by 4x RIPA washes (50 mM Hepes-KOH (pH 8.0), 500 mM LiCl, 1mM EDTA, 1% NP40, 0.7% Na-Deoxycholate, 1x PI) and 1x Na-Cl TE wash (1x TE, 50mM NaCl) at 4°C. Chromatin was eluted from the beads with SDS ChIP elution buffer (50 mM Tris-HCl (pH 7.5), 10 mM EDTA, 1% SDS). Cross-linking was reversed o/n at 70°C in the thermomixer at 1300 rpm. Cellular RNA was digested with RNaseA (0.2 μg/ml) at 37°C for 1 hour, and cellular proteins were removed with Proteinase K (0.4 mg/ml) at 55°C for 2 hours. Chromatin samples were separated from the streptavidin beads and input and ChIP DNA was extracted using a standard phenol-chloroform extraction method. Libraries were prepared using MicropPlex Library Preparation v1 or v2 kit (Diagenode) (75% epiboly - 13 cycles, 1-2ss - 12 cycles, 5-6ss - 12 cycles, BirA-only - 10 cycles, 14ss - 10 cycles of amplification) and sequenced using NextSeq® 500/550 High Output Kit v2 (75 cycles) on NextSeq500 sequencing platform.

ATAC, Library Preparation and Sequencing

FACS sorted cells were lysed (10mM Tris-HCl, pH7.4, 10mM NaCl, 3mM MgCl2, 0.1% Igepal) and tagmented using Nextera DNA kit (Illumina FC-121-1030) for 30mins at 37°C. Tagmented DNA was amplified using NEB Next High-Fidelity 2X PCR Master Mix for 11 cycles. Tagmentation efficiency was assessed using Agilent Tapestation. ATAC-Rx was carried out per ATAC protocol described above with the addition of 50% extra Drosophila S2 cells as reference chromatin (Orlando et al., 2014). ATAC-seq libraries were sequenced using 40 bp PE run on Illumina NextSeq500 platform.

H3K27Ac ChIP, Library Preparation and Sequencing

FACS sorted cells were cross-linked with 1% formaldehyde. Fixation was quenched with 125 mM of glycine for 5min. Cross-linker was washed-out by 3x pellet washes with 1x PBS (with 1X PI, 1 mM DTT and 0.2 mM PMSF) centrifuging at 2000g for 4min at 4°C. Pellets were re-suspended in isotonic nuclei extraction buffer (NEB: 0.5% NP40, 0.25% Triton X, 10 mM Tris-HCl-pH 7.5, 3 mM $CaCl_2$, 0.25 M sucrose, 1mM DTT, 0.2 mM PMSF, 1X Proteinase inhibitors (PIs). Cell nuclei were expulsed with 20 strokes using pestle B in a glass homogeniser, pelleted and washed with 1 xPBS (with 1X PI, 1 mM DTT and 0.2 mM PMSF). Nuclei were lysed in SDS lysis buffer (0.7% SDS, 10mM EDTA, 50 mM Tris-HCl (pH 7.5), 1x PI). Cross-linked chromatin was sonicated at 12A, 10x (10s ON 30s OFF) followed by 8A, 4x (30s ON 30s OFF) and sonicated into 300-800bp fragments. Pre-blocked Protein A Dynabeads were pre-incubated with antibody (Abcam Ab4729) and sonicated DNA-protein complexes were applied to beads o/n at 4°C, IgG antibody (Millipore 12-370) was used as control and an input sample was taken. Samples were washed 6x with RIPA buffer (50 mM Hepes-KOH (pH 8.0), 500 mM LiCl, 1mM EDTA, 1% NP40, 0.7% Na-Deoxycholate, 1x PIs) and 1x NaCl TE wash (1x TE, 50mM NaCl) at 4°C. Chromatin was eluted from the beads with SDS ChIP elution buffer (50 mM Tris-HCl (pH 7.5), 10 mM EDTA, 1% SDS). Cross-linking was reversed o/n at 70°C in the thermomixer at 1300 rpm. Cellular RNA was digested with RNaseA (0.2 μg/ml) at 37°C for 1 hour, and cellular proteins were removed with Proteinase K (0.4 mg/ml) at 55°C for 2 hours. Samples were purified by standard phenol-chloroform extraction and ethanol precipitation. Libraries were prepared using NEBNext® Ultra DNA^{TM} library prep kit according to manufacturer's instructions. Libraries were amplified using (Adli and Bernstein, 2011) protocol for small-cell-number ChIP. H3K27Ac ChIP libraries were sequenced using 50bp PE reads using Illumina Hiseq2500 platform.

Enhancer Reporter Constructs

All enhancer inserts were generated by PCR using KAPA Long Range HotStart PCR kit (Kapa Biosystems) and cloned into the E1b:GFP:Ac/Ds vector using the InFusion kit (InFusion HD Cloning kit, Clontech). Fertilised single-cell embryos were injected with 30pg of plasmid DNA and 25pg of Ac mRNA. Injected embryos were imaged on a Zeiss780 LSM inverted confocal microscope equipped with EC Plan-Neofluar 10x/0.30 NA WD=5.2 (Zeiss) objective or using a Zeiss Axio Scope.A1 equipped with 5x/0.15 NA N-Achroplan or 10x/0.3 NA EC Plan-Neofluar objectives (Zeiss) at desired developmental stages.

Foxd3 Ectopic Expression Assay

40pg of foxd3 mRNA was injected into single cell stage heterozygous *Gt(foxd3-mCherry)*^{ct110} embryos. Whole embryos were collected at 50% epiboly for FACS analysis as described above. For the *foxd3* overexpression followed by ATAC-seq experiments 40pg of *foxd3* mRNA was injected into single cell wild-type fertilised embryos. 50% epiboly embryos were dissected to obtain cells that do not express foxd3 intrinsically: 12,000 cells were used per each experimental/control sample in triplicates. Cells were dissociated with 0.05% trypsin to a single cell suspension, centrifuged and re-suspended in Hanks buffer. Cells were lysed as above and tagmented using Nextera DNA kit (Illumina FC-121-1030) for 15 minutes at 37°C, reactions were quenched with 50mM EDTA for 30 minutes at 50°C. Tagmented DNA was amplified using NEB Next High-Fidelity 2X PCR Master Mix for 15 cycles. Tagmentation efficiency was assessed using Agilent Tapestation and libraries were sequenced using 40bp PE sequencing on Illumina NextSeq500 platform.

Bioinformatic Processing

Bulk RNA-Seq Processing

Reads were trimmed to remove low quality bases using sickle (Joshi and Fass, 2011) when necessary. Read quality was evaluated using FastQC (Barbaraham). Mapping to GRCz10/danRer10 assembly of the zebrafish genome downloaded from UCSC Genome Browser was performed using STAR2.4.2a.(2) (Dobin et al., 2013). Read counts were obtained using subread FeatureCounts(v1.4.6-p4) (Liao et al., 2014) using standard parameters using a gene model gtf derived from Ensembl annotation downloaded from UCSC genome browser. Gene model for ENSDARG00000095311 (the antisense transcript of FoxD3), was removed from gene models. Differential Expression analysis was carried out using in DESeq2 (v.1.14.1) or (v.1.18.1).

Analysis of Single-Cell RNA Sequencing

Short reads (51bp) from 96 cells were aligned to the zebrafish genome (GRCz10/danRer10 assembly) and ERCC spike-in controls using STAR (Dobin et al., 2013) with default parameters. The featureCounts (Liao et al., 2014) was then used to count the number of mapped reads to the reference gene models. Expression values were quantified as read per kilobase of transcript length per million of mapped reads (RPKM) on the basis of Ensembl gene annotation using the "rpkm" function in edgeR (Robinson et al., 2010). We used cells with higher than 100,000 mapping reads and 2,000 detected genes (RPKM>1) for the downstream analysis. With these cut-off criteria, one cell was excluded due to the low sequencing depth. We performed the principal component analysis (PCA) using the custom R script. We selected top 500 genes with the highest absolute correlation coefficient (PCA component loadings) in one of the first three components and then performed PCA and T-distributed stochastic neighbour embedding (tSNE) analyses. The heatmap was visualised on selected gene sets based on the $\log_2$ of RPKM scale using the "pheatmap" function in R. For purpose of single cell transcriptional cataloguing, the scRNA-seq data is visualised using SCDE package (http://hms-dbmi.github.io/scde/index.html) (Fan et al., 2016). Additional analysis was carried out using PAGODA R package (Fan et al., 2016). 50% epiboly demultiplexed scRNASeq data was kindly provided by R.Satija (Satija et al., 2015), and processed as described above. Only *foxd3*-expressing cells from 50% epiboly scRNA-seq dataset were used in analysis.

De Novo *Transcriptome Assembly*

Trinity (v.2.3.2) was run with default parameters on RNA-seq reads from 5-6ss Citrine and 5-6ss Citrine-Cherry after read trimming. FoxD3 ORF truncation was ascertained using blast for a full *foxd3* sequence.

ATAC-Seq Processing

Reads were trimmed for quality using sickle when necessary and mapped using Bowtie (v.1.0.0) (Langmead et al., 2009). Bigwig files were generated using an enhanced Perl script courtesy of Jim Hughes. Peak calling was performed as described previously (Buenrostro et al., 2013). Briefly, BAM files were sorted by name and paired end bed files were obtained using bedtools (v.2.15.0) bamtobed -bedpe. Reads that were not properly paired were discarded and paired reads were displaced by +4 bp and -5 bp. Reads were extended to a read length of 100bp. Peak calling was performed using MACS2 callpeak -f BED -shiftsize=100 -nomodel -slocal 1000 parameters (Zhang et al., 2008). To obtain mappable data, a synthetic 40bp-long single end fastq dataset was generated and mapped using bowtie (v.1.0.0) using –m 1 parameter. Bedgraph files were obtained using bedtools genomeCoverageBed -bg -split function.

MACS2-called peaks that overlapped with regions which in the mappable did not correspond to read size (40bp) were discarded. Identification of peaks corresponding to TSS/promoter, intergenic, intronic and TES locations was carried out using Homer (v.4.8) (Heinz et al., 2010) annotatePeaks.pl script. Only peaks present in both replicates were retained, using bedtools to intersect function to generate reference ATAC-seq ensembles for each stage. ATAC-Rx-seq was processed similarly with the exception that a combined genome of containing danRer10 and Drosophila melanogaster dm6 genomes was created and all reads were mapped to the latter. Zebrafish read counts were normalised as described previously (Orlando et al., 2014). K-means clustering of ATAC-seq signal was carried out using SeqMINER software as described (Ye et al., 2011). In brief, we used non-promoter ATAC-seq peaks form 5-6ss samples as reference points for clustering using following settings: no auto-turning, wiggle step - 15, k-means enrichment linear clustering to cluster given loci presenting similar read densities within the specified window (1500bp on each side of the reference coordinate). Nucleosome localisation was carried out using nucleoATAC suite using default parameters in peaks called at each stage. Bedtools was used to generate bigwig files and clustering of nucleoATAC bigwig signal was carried out using deepTools (v.2.2.2) using k-means clustering with 20 clusters.

For the foxd3 over-expression followed by ATAC-seq: Reads were processed as above. Duplicated reads were removed using MarkDuplicates (picard-tools/1.83). All samples were randomly down-sampled to the lowest-read containing sample (10,443,726) using samtools-1.1. Processed experimental BAM files were merged together as well as control BAM files. *K*-means clustering of ATAC-seq signal was carried out using SeqMINR software as described above (Ye et al., 2011), using 3.1 enhancer cluster ATAC-seq peaks and all pulled foxd3 Biotin-ChIP peaks as references for clustering. Averaged ATAC signal plots were generated using deepTools (v.2.2.2) on the selected *k*-means clusters.

H3K27Ac-ChIP Processing

Reads were trimmed for quality using sickle when necessary and mapped using bowtie (v.1.0.0). Bigwig files were generated using an enhanced Perl script courtesy of Jim Hughes. MACS2 was used to identify peaks using standard parameters. Only peaks present in both replicates were retained, using bedtools intersect function. *k*-means clustering of H3K27Ac signal was carried out using SeqMINER software as described (Ye et al., 2011).

Foxd3 Biotin-ChIP Processing

Foxd3 Biotin-ChIP Processing Reads were trimmed for quality using sickle when necessary and mapped using bowtie (v.1.0.0). Duplicates were removed using MarkDuplicates (picard-tools/1.83). Input reads were normalised to the same number of ChIP reads by random down-sampling using samtools-1.1 (BirA - 18,545,346, 75% epiboly - 28,663,648, 1-2ss - 15,989,800, 5-6ss - 24,270,738, 14ss - 32,322,010 unique reads). Bigwig files were generated using an enhanced Perl script courtesy of Jim Hughes. Peak calling was performed using Homer (v.4.7) (Heinz et al., 2010) findPeaks script using -size 200-minDist 1500 parameters. Peaks were annotated to a nearest expressed gene at a given developmental stage in NC cell population. GO analysis was performed on acquired gene lists in pantherdb.org using statistical overrepresentation binomial test for complete biological processes. Motif discovery and characterisation was performed using Homer screening for *de novo* motifs within given foxd3 Biotin-ChIP-seq peaks. Significantly enriched motifs were annotated manually based on Homer results and levels of gene expression in NC at a corresponding developmental stage. All possible combinations of two motifs were computed using a custom R (v. 3.2.1) script. Homer/4.7 annotatePeaks.pl script was utilised to screen all *de novo* motifs in foxd3 Biotin-ChIP-seq peaks co-occurring in windows of 500bp centred around peaks. A custom Python3 script using the Pandas package (courtesy of Ivan Candido-Ferreira) was used to calculate the frequency of 2-way motif combinations within foxd3-bound ChIP peaks. Combinations enriched at $\chi 2$ P< 5 x 10^{-3} with FDR correction for multiple hypothesis (vs testing co-motif enrichment frequencies against random DNA sequence background) were retained. Two-way networks were plotted using the ‘Circlize’ package in R.

Transcription Factor Binding Site Identification on ATAC-Seq Peaks

Initial Transcription Factor Binding Site (TFBS) enrichment analysis of known motifs was performed using Homer suite (findMotifsGenome.pl) (Heinz et al., 2010). The analysis was performed for all *k*-means clusters, using the default 200bp window centred on the ATAC-peak, and all non-promoter putative regions were used as background. Due to paucity of available zebrafish

transcription factor binding sites (TFBS), a clustering approach of known transcription factors sites was utilized. TFBS for each gene family of interest were downloaded from CIS-BP (http://cisbp.ccbr.utoronto.ca) (Weirauch et al., 2014). Binding sites were clustered using gimme suite's cluster option (v. 0.9.0.3) (van Heeringen and Veenstra, 2011). Background values for each of the clustered motifs were obtained using gimme background function. Cutoff values relative to background sequences were obtained using gimme threshold function. Binding sites were identified using gimme scan function using threshold values obtained from previous step in peaks obtained form ATAC-seq processing.

K-Means Clustering

K-means clustering was performed using the R platform (Ye et al., 2011), by applying the linear enrichment clustering approach to the normalised ATAC-seq datasets and computing the accessibility signal over the non-promoter peaks (+/- 1.5 kb from the centre) using the ensemble of peaks containing both elements common all C replicates, as well as elements common to all CC replicates as a reference. Differences in chromatin accessibility for different *k*-means clusters were quantified by plotting the normalised C and CC ATAC-seq counts for all of putative regulatory elements in cluster and calculating Pearson correlation coefficients. *K*-means clustering investigating dynamics of chromatin opening at the NC *cis*-regulatory elements was performed on 75% epiboly and bud stage ATAC-seq datasets, using called 5-6ss non-promoter ATAC peaks as a reference. Functional annotation of each *k*-means cluster was performed using the GREAT Tool (McLean et al., 2010), using whole genome as background. GREAT employs annotations of putative *cis*-regulatory elements to nearby genes and their statistical integration to infer their function. Statistical significance of associated terms was calculated using binomial and hypergeometric tests and either Bonferroni of False Discovery Rate correction.

Differential Chromatin Accessibility Analysis

The differential chromatin accessibility analysis of ATAC-seq dataset in foxd3-mutant and control conditions was performed using DiffBind package for differential binding analysis of ChIP-seq (Stark and Brown, 2011). Related plots were generated in R. Significantly differentially accessible peaks were identified using the edgeR package, using a reference ATAC-seq peak ensemble. Benjamini–Hochberg multiple testing correction of the resulting p-values was used to derive false discovery rates (FDRs) and only differentially accessible elements with an FDR<0.1 were taken in account.

QUANTIFICATION AND STATISTICAL ANALYSIS

Statistical details of experiments can be found in the figure legends, including p-values and FDR cutoffs. Specific p-values are given in the text where appropriate. Sequencing data, significant differences were defined as an adjusted *p*-value<0.05, unless otherwise noted in the appropriate Method Details sub-section. Statistical analyses were performed in Microsoft Excel or R.

DATA AND SOFTWARE AVAILABILITY

The beta version of the ShinyApp associated with the data produced in this study and Pagoda App (Fan et al., 2016) presenting single cell catalogues can be downloaded from https://github.com/tsslab/foxd3. The live app can be accessed here: https://livedataoxford.shinyapps.io/FoxD3-project-TSS-Lab/. The accession number for the sequencing data generated and reported in this paper is [GEO: GSE106676].

Developmental Cell, Volume *47*

Supplemental Information

From Pioneer to Repressor: Bimodal foxd3 Activity Dynamically Remodels Neural Crest Regulatory Landscape *In Vivo*

Martyna Lukoseviciute, Daria Gavriouchkina, Ruth M. Williams, Tatiana Hochgreb-Hagele, Upeka Senanayake, Vanessa Chong-Morrison, Supat Thongjuea, Emmanouela Repapi, Adam Mead, and Tatjana Sauka-Spengler

Figure S1

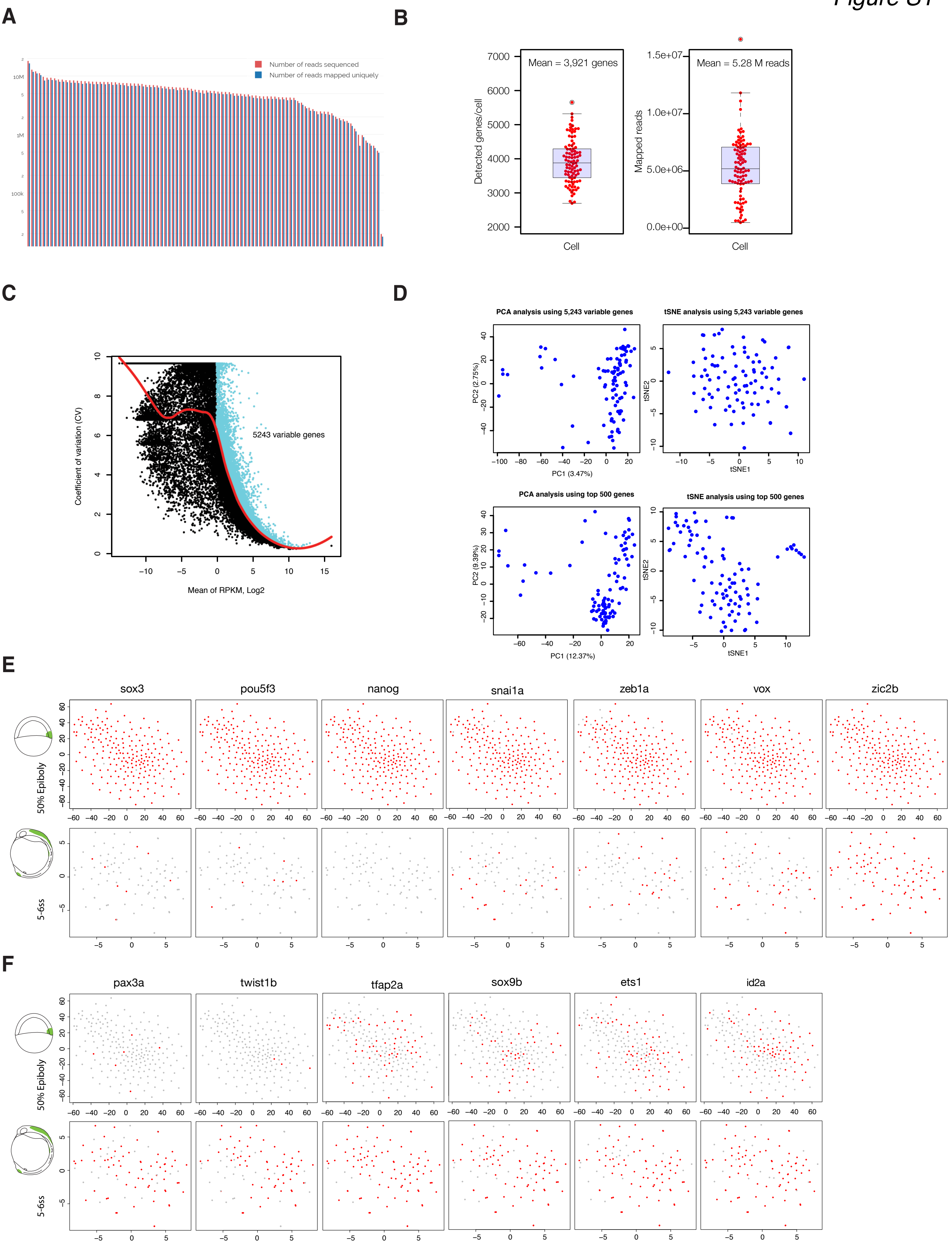
A
Number of reads sequenced
Number of reads mapped uniquely
10M
1M
100k
B
Mean = 3,921 genes
Detected genes/cell
Cell
Mean = 5.28 M reads
Mapped reads
Cell
C
5243 variable genes
Coefficient of variation (CV)
Mean of RPKM, Log2
D
PCA analysis using 5,243 variable genes
tSNE analysis using 5,243 variable genes
PC1 (3.47%)
PC2 (2.75%)
tSNE1
tSNE2
PCA analysis using top 500 genes
tSNE analysis using top 500 genes
PC1 (12.37%)
PC2 (9.39%)
E
sox3
pou5f3
nanog
snai1a
zeb1a
vox
zic2b
50% Epiboly
5-6ss
F
pax3a
twist1b
tfap2a
sox9b
ets1
id2a
50% Epiboly
5-6ss

Figure S1. Single cell transcriptome and quality control (QC) analysis of foxd3-expressing cells from 5-6ss embryos. Related to Figure 1.

(A-B) Grouped bar plot indicating number of sequenced and uniquely mapped reads shows 93/94 single cell transcriptomes are of excellent quality, with a mean of 5.24 M mapped reads and ~4,000 expressed genes mapped per cell.

(C) Identification of 5,243 genes with significantly highly variable expression across single premigratory NCs. Cyan dots indicate genes with a biological coefficient of variation (CV) of >0.50 at a 10% false discovery rate, classified as highly variable; black dots indicate all other genes; red line marks the CV threshold of 0.50 (i.e. the expected position of genes with 50% biological CV).

(D) Principal component analysis (PCA) and T-distributed stochastic neighbor embedding (tSNE) plots all 5,243 or top 500 most divergent genes identifies a small cluster of NC cells.

(E) tSNE plots for selected key pluripotency genes illustrate that while they are expressed at 50% *foxd3+* epiboly stage cells, they are not expressed or are expressed only in a few 5-6ss single *foxd3+* cells, except for *vox* and *zic2b* genes.

(F) tSNE plots for selected key NC specifier genes illustrate that while they are expressed in a majority of 5-6ss single *foxd3+* cells, they are absent or not abundant in 50% epiboly *foxd3+* cells.

Figure S2

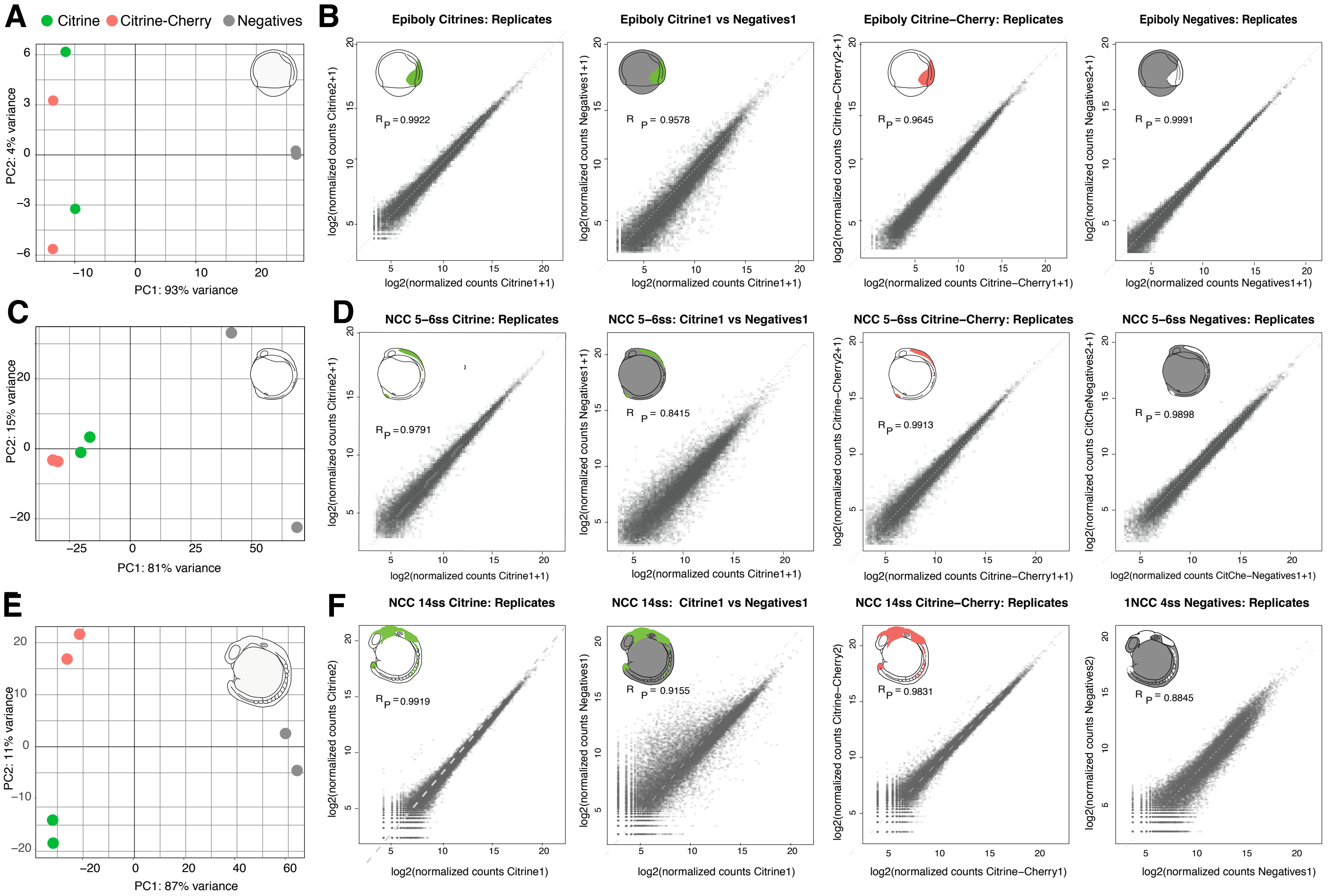
A
Citrine
Citrine-Cherry
Negatives
PC2: 4% variance
PC1: 93% variance
B
Epiboly Citrines: Replicates
Epiboly Citrine1 vs Negatives1
Epiboly Citrine–Cherry: Replicates
Epiboly Negatives: Replicates
log2(normalized counts Citrine1+1)
log2(normalized counts Citrine2+1)
log2(normalized counts Negatives1+1)
log2(normalized counts Citrine–Cherry1+1)
log2(normalized counts Citrine–Cherry2+1)
log2(normalized counts Negatives2+1)
R P = 0.9922
R P = 0.9578
R P = 0.9645
R P = 0.9991
C
PC2: 15% variance
PC1: 81% variance
D
NCC 5–6ss Citrine: Replicates
NCC 5–6ss: Citrine1 vs Negatives1
NCC 5–6ss Citrine–Cherry: Replicates
NCC 5–6ss Negatives: Replicates
log2(normalized counts CitChe–Negatives1+1)
log2(normalized counts CitCheNegatives2+1)
R P = 0.9791
R P = 0.8415
R P = 0.9913
R P = 0.9898
E
PC2: 11% variance
PC1: 87% variance
F
NCC 14ss Citrine: Replicates
NCC 14ss: Citrine1 vs Negatives1
NCC 14ss Citrine–Cherry: Replicates
1NCC 4ss Negatives: Replicates
log2(normalized counts Citrine1)
log2(normalized counts Citrine2)
log2(normalized counts Negatives1)
log2(normalized counts Citrine–Cherry1)
log2(normalized counts Citrine–Cherry2)
log2(normalized counts Negatives2)
R P = 0.9919
R P = 0.9155
R P = 0.9831
R P = 0.8845

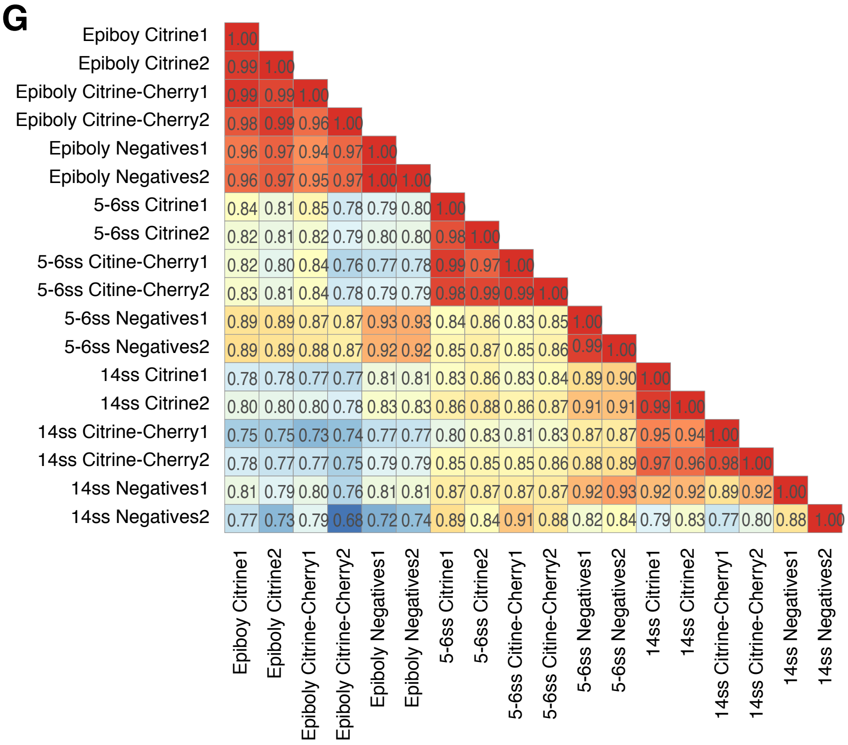
G
Epiboy Citrine1 1.00
Epiboly Citrine2 0.99 1.00
Epiboly Citrine-Cherry1 0.99 0.99 1.00
Epiboly Citrine-Cherry2 0.98 0.99 0.96 1.00
Epiboly Negatives1 0.96 0.97 0.94 0.97 1.00
Epiboly Negatives2 0.96 0.97 0.95 0.97 1.00 1.00
5-6ss Citrine1 0.84 0.81 0.85 0.78 0.79 0.80 1.00
5-6ss Citrine2 0.82 0.81 0.82 0.79 0.80 0.80 0.98 1.00
5-6ss Citine-Cherry1 0.82 0.80 0.84 0.76 0.77 0.78 0.99 0.97 1.00
5-6ss Citine-Cherry2 0.83 0.81 0.84 0.78 0.79 0.79 0.98 0.99 0.99 1.00
5-6ss Negatives1 0.89 0.89 0.87 0.87 0.93 0.93 0.84 0.86 0.83 0.85 1.00
5-6ss Negatives2 0.89 0.89 0.88 0.87 0.92 0.92 0.85 0.87 0.85 0.86 0.99 1.00
14ss Citrine1 0.78 0.78 0.77 0.77 0.81 0.81 0.83 0.86 0.83 0.84 0.89 0.90 1.00
14ss Citrine2 0.80 0.80 0.80 0.78 0.83 0.83 0.86 0.88 0.86 0.87 0.91 0.91 0.99 1.00
14ss Citrine-Cherry1 0.75 0.75 0.73 0.74 0.77 0.77 0.80 0.83 0.81 0.83 0.87 0.87 0.95 0.94 1.00
14ss Citrine-Cherry2 0.78 0.77 0.77 0.75 0.79 0.79 0.85 0.85 0.85 0.86 0.88 0.89 0.97 0.96 0.98 1.00
14ss Negatives1 0.81 0.79 0.80 0.76 0.81 0.81 0.87 0.87 0.87 0.87 0.92 0.93 0.92 0.92 0.89 0.92 1.00
14ss Negatives2 0.77 0.73 0.79 0.68 0.72 0.74 0.89 0.84 0.91 0.88 0.82 0.84 0.79 0.83 0.77 0.80 0.88 1.00
Epiboy Citrine1
Epiboly Citrine2
Epiboly Citrine-Cherry1
Epiboly Citrine-Cherry2
Epiboly Negatives1
Epiboly Negatives2
5-6ss Citrine1
5-6ss Citrine2
5-6ss Citine-Cherry1
5-6ss Citine-Cherry2
5-6ss Negatives1
5-6ss Negatives2
14ss Citrine1
14ss Citrine2
14ss Citrine-Cherry1
14ss Citrine-Cherry2
14ss Negatives1
14ss Negatives2

Figure S2. Biological replicate transcriptome comparisons confirm experimental reproducibility. Related to Figures 1 and 2.

(A, C, E) PCAs and Scatter plots **(B, D, F)** comparing *foxd3*-positive (Citrine), *foxd3*-negative and *foxd3*-mutant (Citrine_Cherry) RNA-seq samples at 3 stages of development (75% epiboly, 5-6ss, 14ss).

(G) Matrix presenting the correlation coefficients to all possible pairwise comparisons of replicates/samples.

Figure S3

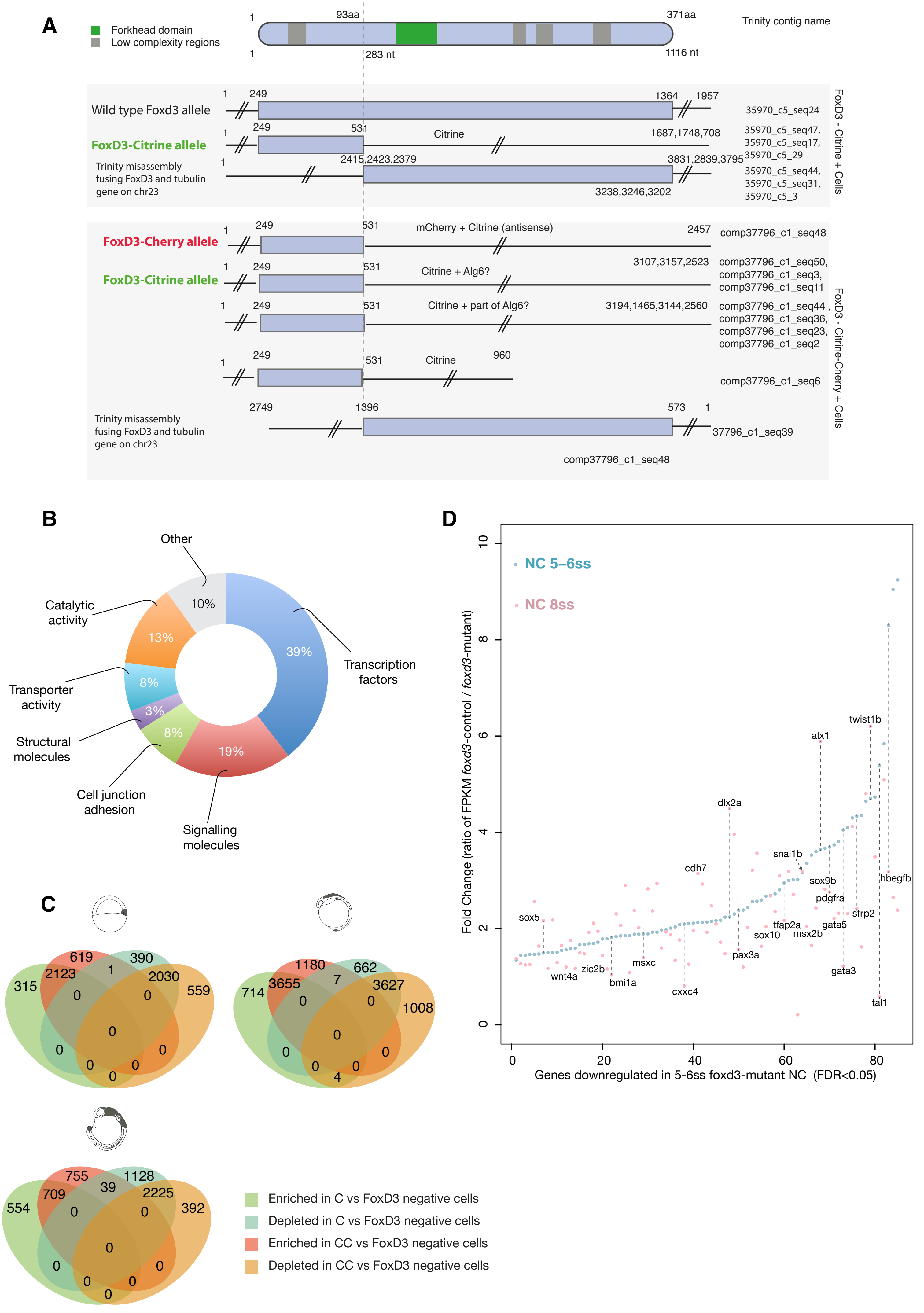
A
Forkhead domain
Low complexity regions
1
93aa
371aa
1
283 nt
1116 nt
Trinity contig name
Wild type Foxd3 allele
1 249 1364 1957
35970_c5_seq24
FoxD3-Citrine allele
1 249 531
Citrine
1687,1748,708
35970_c5_seq47.
35970_c5_seq17,
35970_c5_29
Trinity misassembly fusing FoxD3 and tubulin gene on chr23
1
2415,2423,2379
3831,2839,3795
3238,3246,3202
35970_c5_seq44.
35970_c5_seq31,
35970_c5_3
FoxD3 - Citrine + Cells
FoxD3-Cherry allele
1 249 531
mCherry + Citrine (antisense)
2457
comp37796_c1_seq48
FoxD3-Citrine allele
1 249 531
Citrine + Alg6?
3107,3157,2523
comp37796_c1_seq50,
comp37796_c1_seq3,
comp37796_c1_seq11
1 249 531
Citrine + part of Alg6?
3194,1465,3144,2560
comp37796_c1_seq44 ,
comp37796_c1_seq36,
comp37796_c1_seq23,
comp37796_c1_seq2
1 249 531
Citrine
960
comp37796_c1_seq6
Trinity misassembly fusing FoxD3 and tubulin gene on chr23
2749 1396 573 1
37796_c1_seq39
comp37796_c1_seq48
FoxD3 - Citrine-Cherry + Cells
B
Other
10%
Catalytic activity
13%
Transporter activity
8%
3%
Structural molecules
8%
Cell junction adhesion
19%
Signalling molecules
39%
Transcription factors
C
315
619
2123
390
1
2030
559
0
0
0
0
0
0
0
0
714
1180
3655
662
7
3627
1008
0
0
0
0
0
0
0
4
554
755
709
1128
39
2225
392
0
0
0
0
0
0
0
0
Enriched in C vs FoxD3 negative cells
Depleted in C vs FoxD3 negative cells
Enriched in CC vs FoxD3 negative cells
Depleted in CC vs FoxD3 negative cells
D
NC 5–6ss
NC 8ss
Fold Change (ratio of FPKM foxd3-control / foxd3-mutant)
Genes downregulated in 5-6ss foxd3-mutant NC (FDR<0.05)
0
2
4
6
8
10
0
20
40
60
80
twist1b
alx1
dlx2a
snai1b
cdh7
sox9b
hbegfb
pdgfra
sfrp2
sox5
tfap2a
gata5
sox10
msx2b
pax3a
msxc
zic2b
wnt4a
bmi1a
gata3
cxxc4
tal1

Figure S3. Transcriptomic characterisation of foxd3-citrine and foxd3-cherry mutant cells. Related to Figures 1, 2 and 3.

(A) An illustration of 5-6ss foxd3-mutant protein truncation derived from the *de novo* transcript Trinity assembly. This demonstrates that both foxd3-citrine and foxd3-cherry fusions generate a truncated foxd3 protein only coding for 93 out of 371 amino acids (truncated after 282 nucleotides of coding DNA sequence (CDS)) with no forkhead domain; and hence no protein dimerization or DNA binding is possible. All possible foxd3 transcript variations in the heterozygous foxd3-mutant fish carrying only a foxd3-citrine allele were: (1) foxd3 wt protein (2) truncated foxd3 after the 282nd nucleotide (3) misassembled trinity contigs that have incorrectly merged end of 93rd aa of foxd3 (chr6) with tubulin protein (chr23) due to the design of the FlipTrap cassette. All possible foxd3 transcript variations in the homozygous foxd3-mutant fish carrying foxd3-citrine and foxd3-cherry alleles were: (1) truncated foxd3 protein with Citrine & Alg6 (varying lengths) (2) truncated foxd3 with mCherry and Citrine (antisense) (3) the same misassembled trinity contigs as described before.

(B) The pie chart shows GO Molecular protein function distribution of the down-regulated genes in foxd3 mutants.

(C) Four-way Venn diagrams illustrating differentially expressed genes between both, genes enriched in foxd3-control (citrine - C) and homozygous foxd3-mutants (citrine, cherry - CC) over the negative cells, and depleted genes in foxd3-control (C) and homozygous foxd3-mutants (citrine, cherry - CC) over the negative cells at 75% epiboly, 5-6 somite stage (ss) and 14-16 ss embryos. Differential gene expression analysis was performed using DESeq2 v.1.18.1.

(D) Ranked scatter plot represents downregulated genes ranked from the lowest to the highest based on their differential expression fold change derived from the FPKM values in the 5-6ss foxd3-mutant vs control NC (labelled as blue dots). Pink dots represent the same set of genes (connected by dashed grey lines with blue dots) and their differential expression fold change in the 8ss foxd3-mutant vs control NC.
Since we found that the defect in expression of some, but not all, NC specification factors that were affected in pre-migratory foxd3-mutant NC is lessened, this suggests a possible partial compensation of the NC specification factors as the neural crest cells start delaminating at 8ss.

Figure S4

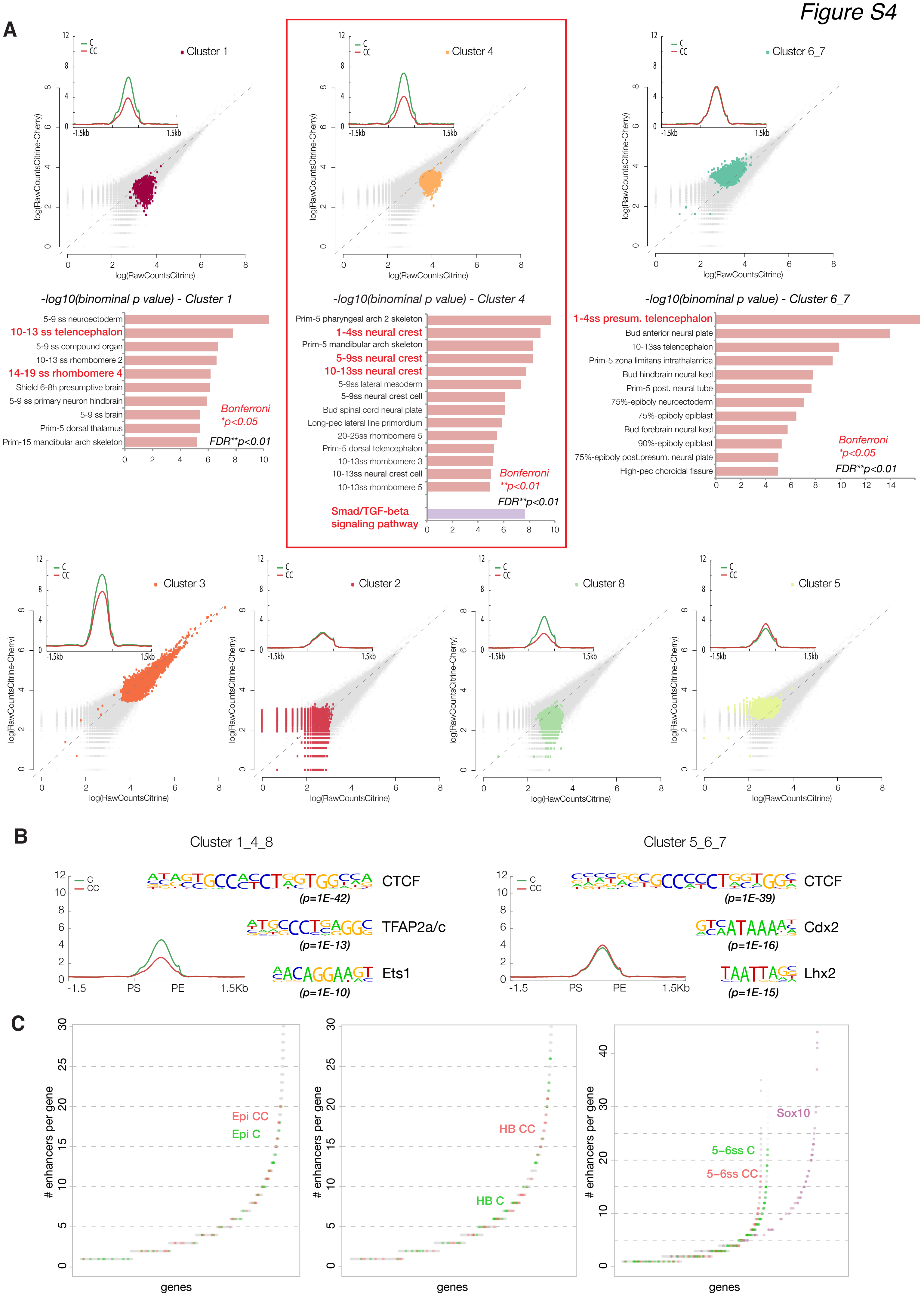
A
Cluster 1
Cluster 4
Cluster 6_7
C
CC
-1.5kb
1.5kb
log(RawCountsCitrine-Cherry)
log(RawCountsCitrine)
-log10(binominal p value) - Cluster 1
5-9 ss neuroectoderm
10-13 ss telencephalon
5-9 ss compound organ
10-13 ss rhombomere 2
14-19 ss rhombomere 4
Shield 6-8h presumptive brain
5-9 ss primary neuron hindbrain
5-9 ss brain
Prim-5 dorsal thalamus
Prim-15 mandibular arch skeleton
Bonferroni *p<0.05
FDR**p<0.01
-log10(binominal p value) - Cluster 4
Prim-5 pharyngeal arch 2 skeleton
1-4ss neural crest
Prim-5 mandibular arch skeleton
5-9ss neural crest
10-13ss neural crest
5-9ss lateral mesoderm
5-9ss neural crest cell
Bud spinal cord neural plate
Long-pec lateral line primordium
20-25ss rhombomere 5
Prim-5 dorsal telencephalon
10-13ss rhombomere 3
10-13ss neural crest cell
10-13ss rhombomere 5
Bonferroni **p<0.01
FDR**p<0.01
Smad/TGF-beta signaling pathway
-log10(binominal p value) - Cluster 6_7
1-4ss presum. telencephalon
Bud anterior neural plate
10-13ss telencephalon
Prim-5 zona limitans intrathalamica
Bud hindbrain neural keel
Prim-5 post. neural tube
75%-epiboly neuroectoderm
75%-epiboly epiblast
Bud forebrain neural keel
90%-epiboly epiblast
75%-epiboly post.presum. neural plate
High-pec choroidal fissure
Bonferroni *p<0.05
FDR**p<0.01
Cluster 3
Cluster 2
Cluster 8
Cluster 5
B
Cluster 1_4_8
Cluster 5_6_7
CTCF
(p=1E-42)
TFAP2a/c
(p=1E-13)
Ets1
(p=1E-10)
CTCF
(p=1E-39)
Cdx2
(p=1E-16)
Lhx2
(p=1E-15)
-1.5
PS
PE
1.5Kb
C
enhancers per gene
genes
Epi CC
Epi C
HB CC
HB C
Sox10
5–6ss C
5–6ss CC

Figure S4. Cluster analysis of differential chromatin accessibility from foxd3 mutant cells vs control. Related to Figure 4.

(A) Mean density maps of merged profiles and corresponding scatterplots of raw counts for all *k*-means clusters featuring putative regulatory non-promoter elements with differential accessibility (ATAC-seq signal levels) in *foxd3*-mutant (CC, Citrine/Cherry) and controls (C, Citrine) at 5-6ss. The clusters were computed by applying *k*-means clustering algorithm from the seqMINER platform. Linear enrichment clustering of normalised ATAC-seq datasets from *foxd3*-mutant and *foxd3*-control NC cells was computed genome-wide over the region covering ± 1.5 kb from the centre of each ATAC-seq peak. Peak Calling the individual replicates and retaining only the peaks present in both replicates was used to generate sets of reference peaks. The datasets were normalised by pooling the replicates for each condition and down-sampling the files to the same number of mapped reads. Clusters 1-4-8 (6,777-4,493-8,245 el.) contain lower signal element with prominent differences between mutant and controls (C>>CC), Pearson corr. R_{Cl1}=0.11; R_{Cl4}=0.16; R_{Cl8}=0.17, clusters 5-67 (8,182-3,905-6,070 el.) comprise elements of equally low signal, but with comparable accessibility (C≈CC), R_{Cl5}=0.17; R_{Cl67}=0.5 and Cluster 3 (17,390 el.) features highly accessible regions with broad ATAC-seq peak distribution that showed intermediate signal decrease in mutant (C>CC), R_{Cl3}=0.96.

Bar plots depict functional annotation of *k*-clusters 1, 4 and 67 showing specific enrichment of zebrafish gene expression ontology terms linked to brain (Clusters 1 and 67) and NC development (Cluster 4). Clusters 2, 5 and 8 did not yield any significant terms. Statistical significance calculated by both binominal and hypergeometric tests (Bonferroni ***p < 0.01*). In particular, elements from *k*-cluster 4 elements that feature moderate accessibility and clear defect in the accessibility in the *foxd3*-mutants, are specifically enriched in the zebrafish gene expression ontology terms linked to all stages of NC development under study here (1-4ss, 5-9ss and 10-13ss), as well as to the brain development. Interestingly, cluster 4 elements were also highly significantly associated to the Smad/TGFbeta signalling pathway (FDR<0.01) and assigned to genes that are normally downregulated in migrating NC and de-repressed in *foxd3*-mutant (*acvr2aa, bmp2b, bmp4, bmpr1a/b, bmpr2b, dand5, foxh1, smad2, smad3a/3b, smad6b, smurf1, tgfb3*; Bonferroni ***p<0.01*). Finally, association to molecules involved in negative regulation of neurogenesis, that were also de-repressed in *foxd3*-mutants by 14ss (*epha4a*, *musk*, *robo2*, *slit3*, *her3*, *her9*, *neurog1*, *notch3*; FDR ***p<0.01*), suggests that Cluster 4 elements could be acting in repression.

(B) Top Transcription Factor Binding Site (TFBS) motifs enriched in other *k*-means clusters of lower accessibility (Clusters 1-4-8 and 5-67). CTCF was the top enriched binding motif (p=1E-39) in. Elements from clusters 1-4-8 that display defective chromatin accessibility in *foxd3*-mutant cells were also enriched in NC motifs, in particular TFAP2a and Ets1, but not Sox10, whereas cluster 5-67 elements (no differential accessibility in foxd3 mutants) harboured a non-crest neural regulatory code (Cdx, Lhx, Hox). Thus the enhancer elements whose accessibility is dependent on foxd3 appear to display unifying features of a NC enhancer.

(C) Plots representing genes assigned to all putative regulatory elements, identified in *foxd3*-mutant (CC) and control (C) cells at 4 stages of development (Epi, 75% epiboly; HB, head reagion of the bud stage embryo 1-2ss; 5-6ss NC and sox10-expressing cells at 16ss) ranked by the number of associated elements.

Putative regulatory elements (all identified non-promoter ATAC-seq elements) identified in *foxd3*-mutant (CC) and control (C) NC were associated to the genes expressed at each corresponding stage and the assigned genes were ranked by the number of elements associated with them. Key developmental factors are most often regulated by multiple *cis*-regulatory elements, acting in concert to yield strong gene expression and defined tissue-specific patterns. Ranking plots show that a large proportion of expressed genes, considered as highly regulated loci, laid beyond the inflexion point on the ranking plot and was assigned a minimum of 3 regulatory elements. Interestingly, while the number of genes and the number of elements assigned per gene at epiboly stage did not differ, by 5-6ss we detected clear increase in expressed genes, elements and number of elements assigned per gene in control NC cells, suggesting that the defect of accessibility at distal cis-regulatory elements was clearly linked to the gene expression differences in *foxd3*-mutant (CC) NC cells. Finally, by late NC differentiation stage, the number of regulated genes and associated elements increases overall.

Figure S5

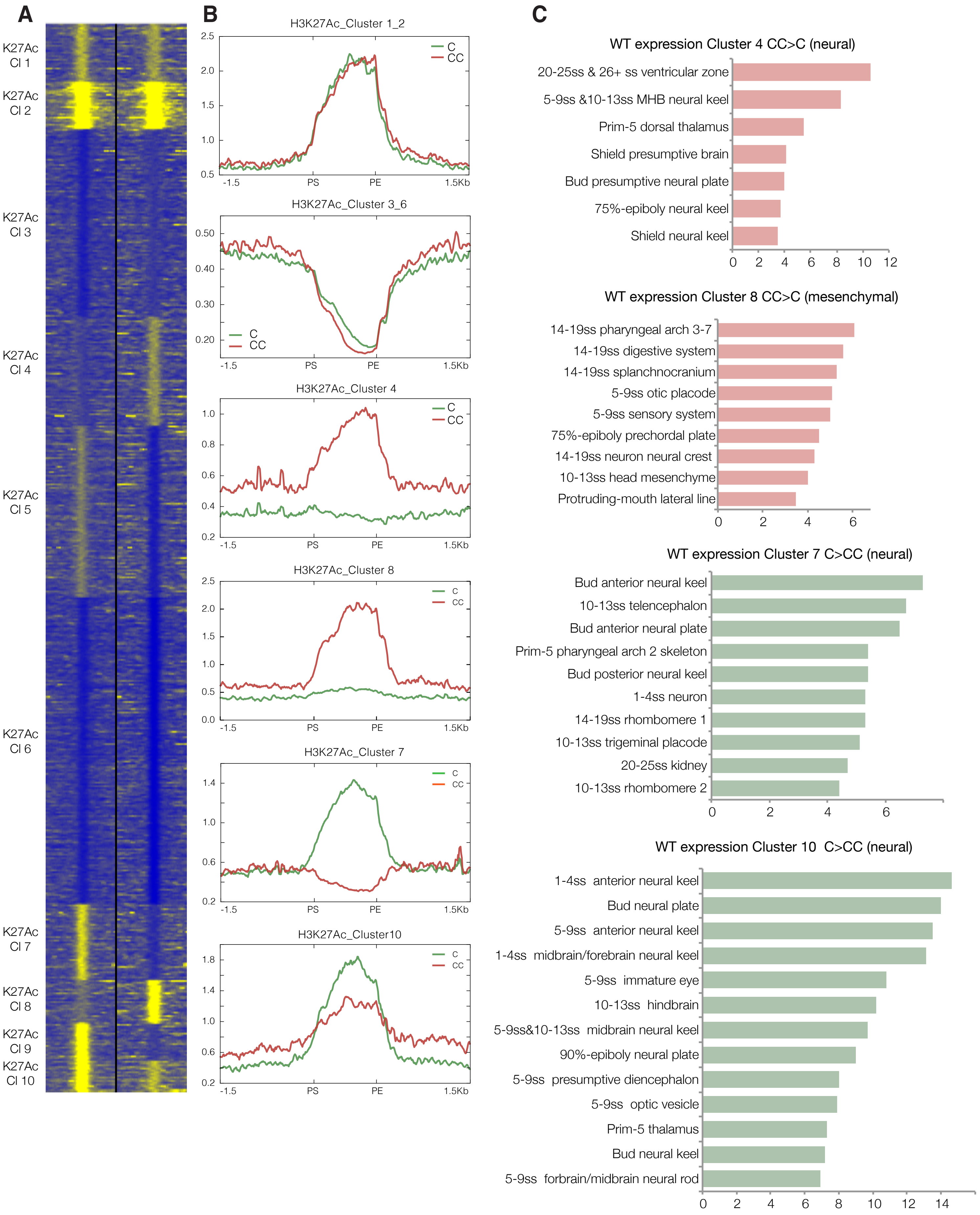

A
K27Ac Cl 1
K27Ac Cl 2
K27Ac Cl 3
K27Ac Cl 4
K27Ac Cl 5
K27Ac Cl 6
K27Ac Cl 7
K27Ac Cl 8
K27Ac Cl 9
K27Ac Cl 10
B
H3K27Ac_Cluster 1_2
H3K27Ac_Cluster 3_6
H3K27Ac_Cluster 4
H3K27Ac_Cluster 8
H3K27Ac_Cluster 7
H3K27Ac_Cluster10
C
CC
-1.5
PS
PE
1.5Kb
C
WT expression Cluster 4 CC>C (neural)
20-25ss & 26+ ss ventricular zone
5-9ss &10-13ss MHB neural keel
Prim-5 dorsal thalamus
Shield presumptive brain
Bud presumptive neural plate
75%-epiboly neural keel
Shield neural keel
WT expression Cluster 8 CC>C (mesenchymal)
14-19ss pharyngeal arch 3-7
14-19ss digestive system
14-19ss splanchnocranium
5-9ss otic placode
5-9ss sensory system
75%-epiboly prechordal plate
14-19ss neuron neural crest
10-13ss head mesenchyme
Protruding-mouth lateral line
WT expression Cluster 7 C>CC (neural)
Bud anterior neural keel
10-13ss telencephalon
Bud anterior neural plate
Prim-5 pharyngeal arch 2 skeleton
Bud posterior neural keel
1-4ss neuron
14-19ss rhombomere 1
10-13ss trigeminal placode
20-25ss kidney
10-13ss rhombomere 2
WT expression Cluster 10 C>CC (neural)
1-4ss anterior neural keel
Bud neural plate
5-9ss anterior neural keel
1-4ss midbrain/forebrain neural keel
5-9ss immature eye
10-13ss hindbrain
5-9ss&10-13ss midbrain neural keel
90%-epiboly neural plate
5-9ss presumptive diencephalon
5-9ss optic vesicle
Prim-5 thalamus
Bud neural keel
5-9ss forbrain/midbrain neural rod

Figure S5. Cluster analysis of differential non-promoter H3K27 acetylation from foxd3 mutant cells vs control. Related to Figure 6.

(A,B) Complete heatmap **(A)** depicting *k*-means linear enrichment clustering of H3K27Ac signal across non-promoter ATAC-seq peaks in foxd3-mutant and control NC at 5-6ss (*k*=10) and **(B)** associated mean density maps of merged profiles of all clusters are shown. Enrichment was computed genome-wide over the region covering ± 1.5 kb from the centre of each ATAC-seq peak, ATAC-seq reference set as described in Figure S4 was used. We identified clusters with increased H3K27Acetylation in the foxd3 mutant (C<CC, K27Ac_Clusters 4 and 8) and clusters with decreased K3K27Ac signal in foxd3 mutants (C>CC, K27AC-Clusters 5, 7, 9 and 10). **(C)** Bar plots depicting functional annotation of K27Ac_Clusters 4, 8, 7 and 10 depicting specific enrichment of zebrafish gene expression ontology terms obtained by GREAT tool are shown (Bonferroni $^{**}p < 0.01$).

Figure S6

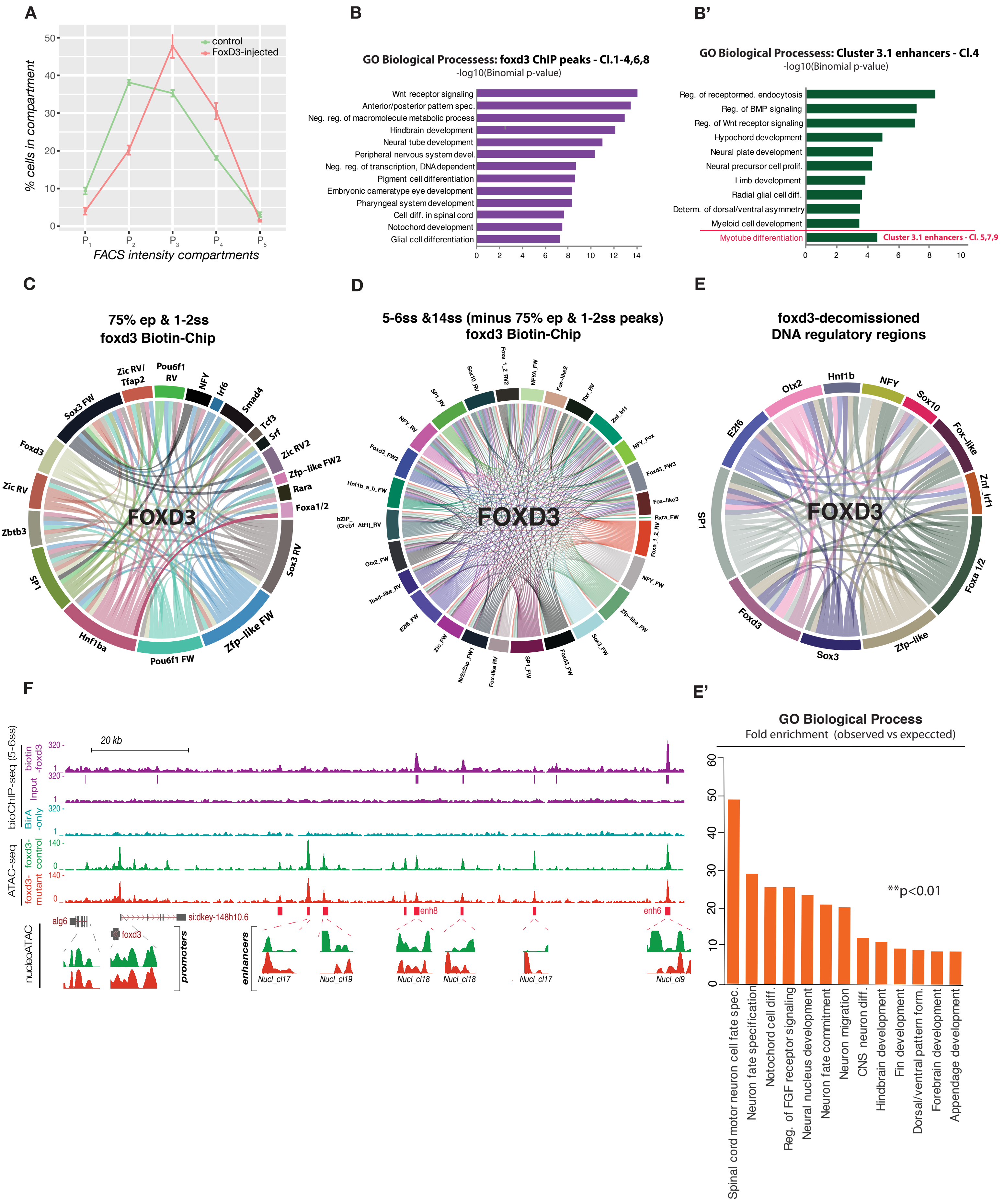
A
% cells in compartment
control
FoxD3-injected
FACS intensity compartments
B
GO Biological Processess: foxd3 ChIP peaks - Cl.1-4,6,8
-log10(Binomial p-value)
Wnt receptor signaling
Anterior/posterior pattern spec.
Neg. reg. of macromolecule metabolic process
Hindbrain development
Neural tube development
Peripheral nervous system devel.
Neg. reg. of transcription, DNA dependent
Pigment cell differentiation
Embryonic cameratype eye development
Pharyngeal system development
Cell diff. in spinal cord
Notochord development
Glial cell differentiation
B'
GO Biological Processess: Cluster 3.1 enhancers - Cl.4
-log10(Binomial p-value)
Reg. of receptormed. endocytosis
Reg. of BMP signaling
Reg. of Wnt receptor signaling
Hypochord development
Neural plate development
Neural precursor cell prolif.
Limb development
Radial glial cell diff.
Determ. of dorsal/ventral asymmetry
Myeloid cell development
Myotube differentiation
Cluster 3.1 enhancers - Cl. 5,7,9
C
75% ep & 1-2ss
foxd3 Biotin-Chip
FOXD3
D
5-6ss &14ss (minus 75% ep & 1-2ss peaks)
foxd3 Biotin-Chip
FOXD3
E
foxd3-decomissioned
DNA regulatory regions
FOXD3
F
bioChIP-seq (5-6ss)
ATAC-seq
nucleoATAC
20 kb
promoters
enhancers
E'
GO Biological Process
Fold enrichment (observed vs expeccted)
**p<0.01
Spinal cord motor neuron cell fate spec.
Neuron fate specification
Notochord cell diff.
Reg. of FGF receptor signaling
Neural nucleus development
Neuron fate commitment
Neuron migration
CNS neuron diff.
Hindbrain development
Fin development
Dorsal/ventral pattern form.
Forebrain development
Appendage development

Figure S6. Putative mechanisms of the foxd3 bimodality and potential co-interactors for NC gene regulation. Related to Figure 7.

(A) FACS graph portraying a number of foxd3-mCherry expressing cells and underlying fluorescence intensities of control (green) and foxd3 mRNA injected (pink) embryos. P1-P5 – compartments of different fluorescence levels from the lowest to the highest. The graph illustrates an overall cellular fluorescence increase upon foxd3 overexpression in zebrafish embryos.

(B,B′) Bar plots showing gene ontology (GO) biological processes derived from GREAT analysis that are associated with the regulation of DNA-elements showing increased accessibility upon foxd3 overexpression. **(B)** These regulatory sequences were shown to be directly bound by foxd3 by our biotin ChIP-seq analysis (merged 1-4,6,8 k-mean subclusters (out of eight subclusters) showing similar enhanced accessibility signature that were clustered using all foxd3 possible foxd3 ChIP-seq peaks from our data sets). **(B′)** These regulatory sequences belong to the core NC enhancer cluster 3.1 (subcluster 3.1_4 elements and merged comparable signature subclusters 3.1_5, 7, 9 (single GO term under the red line) showing enhanced accessibility; they were obtained by k-mean analysis (out of ten 3.1 subclusters)).

(C,D) Circle plots showing different significant non-combined transcription factor (TF) motif enrichment underlying the **(C)** 'early NC' foxd3 biotin ChIP-seq peaks and statistically significant 2-way combinations between these factors that are hypothesised to act together with foxd3 during its activator stage and **(D)** 'late NC' foxd3 biotin ChIP-seq peaks and statistically significant 2-way combinations between these factors that are hypothesised to act together or in competition with foxd3 during its repressive stage.

(E) Circle plot showing significant 2-way TF co-interactions on the regulatory elements that get directly decommissioned by foxd3. **(E′)** Bar plot showing gene ontology (GO) biological terms significantly enriched (**$p<0.01$) to the nearest expressed genes of the same DNA regulatory elements that get directly compacted upon foxd3 binding by the 14ss.

(F) Nucleosomal occupancy tracks (NucleoATAC) showing nucleosome positions within the regulatory elements (promoters and enhancers) in foxd3 mutant (red) and control (green) cells.
